# Supplementary material for: STIMULATE-ICP: A pragmatic, multi-centre, cluster randomised trial of an integrated care pathway with a nested, Phase III, open label, adaptive platform randomised drug trial in individuals with Long COVID: A structured protocol
Source: PLoS One. 2023 Feb 15;18(2):e0272472. doi: 10.1371/journal.pone.0272472 (PMC9931100; doi:10.1371/journal.pone.0272472)
Supplement: S1 File — (DOCX) [file pone.0272472.s001.docx]

**A pragmatic, multi-centre, cluster randomised trial of an integrated care pathway with a nested, Phase III, open label, adaptive platform randomised drug trial in individuals with Long COVID**

STIMULATE-ICP

**PROTOCOL VERSION: 2.1 (05/05/2022)**

**This protocol has regard for HRA guidance and order of content.**

# RESEARCH REFERENCE NUMBERS

| **IRAS Number:** | 1004698 |
| --- | --- |
| **EudraCT Number:** | 2021-006598-47 |
| **SPONSOR:** | University College London (UCL) |
| **SPONSOR Number:** | 143602 |
| **REC Reference Number:** | 21/SC/0416 |
| **FUNDERS:** | National Institute for Health Research  Perspectum |
| **FUNDERS Number:** | 29622 NIHR COV-LT2-0043 |

# SIGNATURE PAGE

The undersigned confirm that the following protocol has been agreed and accepted and that the Chief Investigator agrees to conduct the trial in compliance with the approved protocol and will adhere to the principles outlined in the Medicines for Human Use (Clinical Trials) Regulations 2004 (SI 2004/1031), amended regulations (SI 2006/1928) and any subsequent amendments of the clinical trial regulations, the UK Policy Framework for Health and Social Care Research 3rd edition 2017 (as amended), GCP guidelines, the UK Data Protection Act (2018), the Sponsor’s (and any other relevant) SOPs, and other regulatory requirements as amended.

I also confirm that I will make the findings of the trial publicly available through publication or other dissemination tools without any unnecessary delay and that an honest accurate and transparent account of the trial will be given; and that any discrepancies and serious breaches of GCP from the trial as planned in this protocol will be explained.

| **Chief Investigator:** |
| --- |
| Signature and Date: |
|  |
| Name (please print): |
| **Professor Amitava Banerjee** |
|  |
|  |
| **For and on behalf of the Trial Sponsor:** |
| Signature and Date: |
|  |
| Name (please print): |
|  |
| Position: |
|  |

# PROTOCOL VERSION HISTORY

| **Version Number** | **Date** | **Protocol Update Finalised By**  (insert name of person) | **Reasons for Update** |
| --- | --- | --- | --- |
| 1.0 | 10.12.2022 | Denise Forshaw | Submitted to MHRA and HRA combined review for approval |
| 1.1 | 04.01.2022 | Denise Forshaw | Updated in response to MHRA and HRA combined review letter |
| 2.0 | 07.03.2022 | Denise Forshaw | Updated for inclusion of Colchicine and Rivaroxaban to the randomisation arms of the trial and associated changes in statistical analysis, optional blood sample collections |
| 2.1 | 29.04.2022 | Denise Forshaw | Updated contents page numbering and the creatinine clearance rate had been changed from <30ml/min to <15ml/min to reflect the introduction of the Rivaroxaban and Colchicine arms |
|  |  |  |  |
|  |  |  |  |

# KEY TRIAL CONTACTS

| **Chief Investigator** | Professor Amitava Banerjee  Institute of Health Informatics University College London 222 Euston Road  London NW1 2DA |
| --- | --- |
| **Sponsor** | Joint Research Office, UCL, 4th Floor West,  250 Euston Road  London NW1 2PG Postal address: Joint Research Office, Gower Street, London WC1E 6BT |
| **Clinical Trials Unit** | Lancashire Clinical Trials Unit, University of Central Lancashire, Preston PR1 2HE |
| **Statisticians** | Dr Gordon Prescott Deputy Director of Lancashire Clinical Trials Unit, The University of Central Lancashire, Preston PR1 2HE  Dr Hakim-Moulay Dehbi University College London, Comprehensive Clinical Trials Unit, London. |
| **Principal Clinical Trial Manager** | Denise Forshaw, Deputy Director Lancashire Clinical Trials Unit, University of Central Lancashire, Preston PR1 2HE |
| **Central Laboratories** | Oxford Community Diagnostic Laboratory (Perspectum)  Perspectum,  Gemini One, 5520 John Smith Drive,  Oxford, OX4 2LL,  United Kingdom  Science Technology Platform,  High-Throughput Screening Laboratory  Francis Crick Institute  1 Midland Road, London NW1 1AT |
| **Central Pharmacy / IMP** | Study drugs to be issued by prescription from participating site pharmacy or clinic setting. An individualised plan will be put in place for each setting. |
| **Collaborators / Co-Investigators** | Professor Christina Van der Feltz-Cornelis  Professor Dame Caroline Watkins  Professor Dan Cuthbertson  Professor Elizabeth Murray  Professor Gregory Lip  Professor Hugh Montgomery  Professor Kamlesh Khunti  Professor Mark Gabbay  Professor Nefyn Williams  Professor Paula Lorgelly  Professor Sir Michael Brady  Dr Angela Green  Dr Dan Wootton  Dr Emily Attree  Dr Emma Wall  Dr Gail Allsopp  Dr Melissa Heightman  Dr Michael Crooks  Dr Michael Zandi  Dr Nisreen Alwan  Dr Toby Hillman  Dr William Strain  Mr Lyth Hishmeh |
| **Trial Management Group** | Professor Amitava Banerjee (CI)  Dr Melissa Heightman (UCLH:Co-CI)  Denise Forshaw (UCLan)  Dr Gordon Prescott (UCLan)  Senior Clinical Trials Manager (LCTU)  Data Manager (LCTU)  Sponsor Representatives (UCL) |
| **Trial Steering Committee** | Independent members;  Professor Patrick Mallon (Paddy) – St Vincent University Hospital (Dublin) - Chair  Dr Marion Mafham – Nuffield Department of Population Health University of Oxford  Ondine Sherwood – Founder of Long COVID SoS and Public and Patient Involvement (PPI) Representative  Supported by Trial Managment Group members:  Professor Amitava Banerjee (CI) (University College London)  Dr Melissa Heightman (University College London Hospitals NHS FT:Co-CI)  Denise Forshaw (University of Central Lancashire )  Dr Gordon Prescott (University of Central Lancashire)  Professor Matthew Sydes – (University College London )  University College London – Sponsor Representative |
| **Independent Data Monitoring Committee** | Dr Christopher Sutton (University of Manchester)  Professor Tim Peters (University of Bristol)  Prof Peter Langhorne (University of Glasgow)  Dr Ly-Mee Yu (University of Oxford) |

# TABLE OF CONTENTS

[RESEARCH REFERENCE NUMBERS 2](#_Toc103248714)

[SIGNATURE PAGE 3](#_Toc103248715)

[PROTOCOL VERSION HISTORY 4](#_Toc103248716)

[KEY TRIAL CONTACTS 5](#_Toc103248717)

[i. TABLE OF CONTENTS 8](#_Toc103248718)

[ii. LIST OF ABBREVIATIONS 11](#_Toc103248719)

[iii. TRIAL SUMMARY 13](#_Toc103248720)

[iv. KEY ROLES AND RESPONISIBILITIES 18](#_Toc103248721)

[v. KEY WORDS 19](#_Toc103248722)

[1. BACKGROUND 20](#_Toc103248723)

[2. RATIONALE 22](#_Toc103248724)

[2.1. Further Rationale for the Trial and Interventions 23](#_Toc103248725)

[2.2. Rationale for the Use of Drugs in STIMULATE-ICP Trial and Underlying Data 23](#_Toc103248726)

[2.3. Predicted Efficacy of the Drugs in Long COVID 24](#_Toc103248727)

[3. RISK ASSESSMENT AND MANAGEMENT OF RISKS 27](#_Toc103248728)

[3.1. Risks Associated with Patient Population in the Trial 27](#_Toc103248729)

[3.2. Risks Associated with non-IMP (Intervention) Component of the Trial 27](#_Toc103248730)

[3.3. Safety Profile of the Trial IMPs 28](#_Toc103248731)

[4. OBJECTIVES AND OUTCOME MEASURES/ENDPOINTS 32](#_Toc103248732)

[4.1. Primary Objectives 32](#_Toc103248733)

[4.2. Secondary Objectives 32](#_Toc103248734)

[4.3. Primary Outcome Measure 32](#_Toc103248735)

[4.4. Secondary Outcome Measures 32](#_Toc103248736)

[4.5. Exploratory Outcome Measures / Endpoints 34](#_Toc103248737)

[5. TRIAL DESIGN 34](#_Toc103248738)

[5.1. Definitions 34](#_Toc103248739)

[5.2. Justification for Cluster Randomisation 35](#_Toc103248741)

[5.3. Design, Implementation and Evaluation of Integrated Care 35](#_Toc103248742)

[5.4. Trial setting 39](#_Toc103248743)

[6. INVESTIGATIONAL MEDICINAL PRODUCTS 39](#_Toc103248744)

[6.1. Name and Description of IMP(s) 39](#_Toc103248745)

[6.2. Formulation, Packaging and labelling of the IMPs 40](#_Toc103248746)

[6.3. Source of IMP, Manufacture and Distribution 40](#_Toc103248747)

[6.4. Storage and Handling of IMP(s) at Site 40](#_Toc103248748)

[6.5. Accountability of IMP(s) 40](#_Toc103248749)

[6.6. Regulatory Status of the IMP(s) 41](#_Toc103248750)

[6.7. Dosage Schedules 41](#_Toc103248751)

[6.8. Drug Interactions and Specific Contraindications 41](#_Toc103248752)

[6.9. Consideration of Dose Modification 46](#_Toc103248756)

[6.10. Concomitant Medications 46](#_Toc103248757)

[7. TRIAL PROCEDURES 46](#_Toc103248758)

[7.1. Selection of Participants 46](#_Toc103248759)

[7.2. Participant Eligibility Criteria 46](#_Toc103248760)

[7.3. Participant Identification 49](#_Toc103248761)

[7.4. Recruitment of Trial Participants 49](#_Toc103248762)

[8. REGISTRATION / RANDOMISATION PROCEDURES 53](#_Toc103248763)

[8.1. Patient Registration 53](#_Toc103248764)

[8.2. Cluster Randomisation for Usual Care service uplift 53](#_Toc103248765)

[8.3. Drug Trial Randomisation 53](#_Toc103248766)

[8.4. Blinding 54](#_Toc103248767)

[9. TRIAL ASSESSMENTS 54](#_Toc103248768)

[9.1. Eligibility Assessments 54](#_Toc103248769)

[9.2. Baseline Visit 55](#_Toc103248770)

[9.3. Assessment Visit (12 Weeks Assessment) 57](#_Toc103248771)

[9.4. Follow-up Assessment (24 Weeks) Visit 58](#_Toc103248772)

[9.5. Laboratory Procedures 59](#_Toc103248773)

[9.6. Schedule of Assessments 62](#_Toc103248774)

[9.7. Post-Trial Treatment 64](#_Toc103248775)

[10. DISCONTINUATION / EARLY CESSATION OF TREATMENT AND “STOPPING RULES” 65](#_Toc103248776)

[10.1. Replacements 65](#_Toc103248777)

[10.2. Cessation of Treatment and Withdrawal 65](#_Toc103248778)

[10.3. Lost to Follow-up 66](#_Toc103248779)

[11. SAFETY REPORTING / PHARMACOVIGILANCE 66](#_Toc103248780)

[11.1. Definitions 66](#_Toc103248781)

[11.2. Recording and reporting of Adverse Events 68](#_Toc103248782)

[11.3. Assessing Adverse Events 68](#_Toc103248783)

[11.4. Assessing Serious Adverse Events and Serious Adverse Reactions (nested drug trial participants only) 69](#_Toc103248784)

[11.5. Serious Adverse Events Which Do Not Require Reporting to Sponsor 70](#_Toc103248785)

[11.6. SUSAR Reporting 70](#_Toc103248786)

[11.7. Adverse Event Recording and Processing Flow Chart 72](#_Toc103248789)

[11.8. Notification of deaths (nested drug trial participants only) 73](#_Toc103248790)

[11.9. Pregnancy reporting (nested drug trial participants only) 73](#_Toc103248791)

[11.10. Overdose Reporting 73](#_Toc103248792)

[11.11. Loratadine Overdose 74](#_Toc103248793)

[11.12. Famotidine Overdose 74](#_Toc103248794)

[11.13. Colchicine Overdose 74](#_Toc103248795)

[11.14. Rivaroxaban Overdose 75](#_Toc103248796)

[11.15. New Safety Findings 76](#_Toc103248797)

[11.16. Urgent Safety Measures 76](#_Toc103248798)

[11.17. Development of Safety Update Reports 76](#_Toc103248799)

[11.18. Responsibilities 76](#_Toc103248800)

[12. DATA MANAGEMENT AND QUALITY ASSURANCE 77](#_Toc103248801)

[13. DATA COLLECTION AND SOURCE DOCUMENT IDENTIFICATION 78](#_Toc103248802)

[13.1. Data Collection from Coverscan™ 79](#_Toc103248803)

[13.2. Data Collection from Long COVID Clinics 79](#_Toc103248804)

[13.3. Linked National Data Resource (Healthcare Systems Data) 79](#_Toc103248805)

[13.4. Completing Case Report Forms 79](#_Toc103248806)

[13.5. Data Handling and Analysis 80](#_Toc103248807)

[13.6. Access to the final trial dataset 80](#_Toc103248808)

[14. STATISTICAL CONSIDERATIONS 80](#_Toc103248809)

[14.1. Sample Size Calculation – Main study 80](#_Toc103248810)

[14.2. Sample Size Calculation – Nested Drug Trial 81](#_Toc103248811)

[14.3. Planned Recruitment Rate 81](#_Toc103248812)

[14.4. Randomisation Methods 82](#_Toc103248813)

[14.5. Statistical Analysis Plan 82](#_Toc103248814)

[14.6. Summary of Baseline Data and Flow of Participants 82](#_Toc103248815)

[14.7. Primary Outcome Analysis 82](#_Toc103248816)

[14.8. Secondary Outcome Analysis 83](#_Toc103248817)

[14.9. Sensitivity and other planned analysis 84](#_Toc103248818)

[14.10. Interim Analysis 84](#_Toc103248819)

[15. END OF TRIAL 84](#_Toc103248820)

[16. RECORD KEEPING AND ARCHIVING 85](#_Toc103248821)

[17. OVERSIGHT COMMITTEES 85](#_Toc103248822)

[17.1. Trial Management Group (TMG) 85](#_Toc103248823)

[17.2. Trial Steering Committee (TSC) 86](#_Toc103248824)

[17.3. Independent Data Monitoring Committee (IDMC) 86](#_Toc103248825)

[17.4. Stopping Rules 86](#_Toc103248826)

[18. MONITORING, AUDIT, AND INSPECTION 87](#_Toc103248827)

[18.1. Direct Access to Source Data 87](#_Toc103248828)

[19. ETHICS AND REGULATORY REQUIREMENT REPORTING 87](#_Toc103248829)

[19.1. Peer Review 88](#_Toc103248830)

[**19.2.** **Public and Patient Involvement** 88](#_Toc103248831)

[**19.3.** **Regulatory Compliance** 88](#_Toc103248832)

[**19.4.** **Data Protection and Participant Confidentiality** 90](#_Toc103248833)

[**19.5.** **Financial and other competing interests for the Chief Investigator & PIs at each site** 91](#_Toc103248834)

[**19.6.** **Insurance and Indemnity** 92](#_Toc103248835)

[20. PUBLICATION & DISSEMINATION 92](#_Toc103248836)

[20.1. Publication Policy 92](#_Toc103248837)

[20.2. Open Access and Data Sharing 93](#_Toc103248838)

[20.3. Authorship Eligibility 93](#_Toc103248839)

[21. REFERENCES 95](#_Toc103248840)

[APPENDICES 99](#_Toc103248841)

[Appendix I 99](#_Toc103248842)

[Appendix II 111](#_Toc103248843)

# LIST OF ABBREVIATIONS

AE Adverse Event

AR Adverse Reaction

BD Twice Daily

CA Competent Authority

CI Chief Investigator

CRF Case Report Form

CTA Clinical Trial Authorisation

CTIMP Clinical Trial of Investigational Medicinal Product

CTU Clinical Trials Unit

DHSC Department of Health & Social Care

DI Designated Individual

DSUR Development Safety Update Report

EC European Commission

EMA European Medicines Agency

EQ-5D-5L Euroqol 5 level instrument (5 questions on mobility, self-care, usual activities, pain or discomfort and anxiety or depression)

EU European Union

EUCTD European Clinical Trials Directive

EudraCT European Clinical Trials Database

EudraVigilance European database for Pharmacovigilance

GAD-7 Generalised Anxiety Disorder-7 questionnaire

GAfREC Governance Arrangements for NHS (National Health Service)
 Research Ethics

GCP Good Clinical Practice

GP General Practitioner

HRA Health Research Authority

IC Integrated Care

ICP Integrated Care Pathway

ICF Informed Consent Form

ICH International Council on Harmonisation of technical requirements for registration of pharmaceuticals for human use

IDMC Independent Data Monitoring Committee

IMP Investigational Medicinal Product

IMPD Investigational Medicinal Product Dossier

IPR Individual Patient Randomisation

ISF Investigator Site File

ISRCTN International Standard Randomised Controlled Trials Number

MHRA Medicines and Healthcare products Regulatory Agency

MWSAS Modified Work and Social Adjustment Scale

NHS R&D National Health Service Research & Development

OD Once Daily

PCN Primary Care Network

PCS Post COVID Syndrome

PDQ Perceived Deficit Questionnaire

PHQ-9 The Primary Care Evaluation of Mental Disorders Patient Health Questionnaire

PI Principal Investigator

PIS Participant Information Sheet

QA Quality Assurance

QC Quality Control

QP Qualified Person

RCT Randomised Control Trial

REC Research Ethics Committee

RSI Reference Safety Information

SAE Serious Adverse Event

SAR Serious Adverse Reaction

SDV Source Data Verification

SF12 Short Form Questionnaire 12 questions

SOP Standard Operating Procedure

SmPC Summary of Product Characteristics

SUSAR Suspected Unexpected Serious Adverse Reaction

TMF Trial Master File

TMG Trial Management Group

TSC Trial Steering Committee

UC Usual Care or Standard Care

UCLH University College London NHS Foundation Trust

USM Urgent Safety Measure

WOCBP Women of Childbearing Potential

WP Work Package

WSAS Work and Social Adjustment Scale

# TRIAL SUMMARY

| **NIHR Project Title** | **Symptoms, Trajectory, Inequalities and Management: Understanding Long-COVID to Address and Transform Existing Integrated Care Pathways** |
| --- | --- |
| **Trial Title** | A pragmatic, multi-centre, cluster-randomised trial of an integrated care pathway with a nested Phase III, open label, adaptive platform, randomised drug trial in individuals with Long COVID. |
| **Short Title** | STIMULATE-ICP |
| **Sponsor Protocol Number** | 143602 |
| **Active IMPs** | Famotidine, Loratadine, Colchicine and Rivaroxaban |
| **Formulation, Dose, Route of Administration** | Famotidine (40mg) once daily by mouth + Loratadine (10mg) tablets once daily by mouth, or Colchicine (500mcg) tablet twice daily by mouth, or Rivaroxaban (10mg) tablet once daily by mouth |
| **Trial Participants** | Individuals with Long COVID referred to participating Long COVID clinic, presenting at first referral (in person or virtual) |
| **Evaluable Sample Size** | Maximum 4,520 in the nested drug trial |
| **Estimated Total Trial Duration** | 24 months |
| **Estimated Trial Duration per Participant** | 24 Weeks (data collection will continue to 12 months via NHS digital) |
| **Trial Objectives** | Primary:  To evaluate: “integrated care” with combinations of multi-organ MRI (Coverscan™) and clinical decision support; and digitally enabled, community rehabilitation: Living with COVID Recovery™ versus “usual care” [Usual Investigations; and self-management website].  Secondary:  To evaluate:   1. Clinical efficacy of the individual components of the integrated care pathway including potential therapies. 2. Effect of the ICP on mean measured health related quality of life, mental health (including depression), work and social adjustment, physical function, organ impairment and healthcare utilisation through patient reported outcomes. 3. To characterise the pathophysiology and trajectory of long COVID |
| **Trial Design** | Pragmatic, cluster-randomised trial with nested, Phase III, open‑label, adaptive platform drug trial. (using a platform approach, new drugs may be added to the protocol, pending approval from NIHR and Department of Health and Social Care (DHSC)). |
| **Trial Methods** | 1. In a pragmatic, cluster (primary care network-level)-randomised trial, we will evaluate integrated care pathway combinations with early, supported investigation (multi-organ MRI with Coverscan™ and clinical decision support) and enhanced rehabilitation (Living with COVID Recovery™), compared with usual care (Usual Investigations and Your COVID Recovery™ self-management), recruiting adults with suspected Long COVID at referral.      1. In a nested, patient-level, adaptive platform drug trial, we will investigate famotidine plus loratadine in combination, or Colchicine, or Rivaroxaban, versus standard of care, with the option to test further drugs using an adaptive platform.   The primary outcome measure is Fatigue Assessment Scale at 12 weeks.   1. Long COVID is a new disease or syndrome and mechanisms of disease and potential mechanisms of new treatments need to be analysed. Baseline blood samples will be taken from all participants in the study to allow our team to look at the underlying disease processes. This will involve standard clinical tests (e.g. haematology and biochemistry) as well as looking at research areas such as genomics. Samples will be stored to develop a biobank for use by the broader global research community in the largest study of long COVID patients to-date. |
| **Clinical Phase** | Phase III |
| **Inclusion & Exclusion Criteria** | **Participant Inclusion Criteria for ALL Participants**   1. Participants capable of giving informed consent. 2. Age 18 years and above 3. Clinical Parameters; persistent signs and symptoms for a period of 4 weeks or longer in duration post-COVID-19 infection (either by test result or symptomology). Presenting at their first referral first visit to a participating Long COVID clinic pathway. 4. Able to read or understand English or have a relative/family member able to read/understand English to facilitate participation (essential for patient reported outcome measures at follow-up time points and virtual contact).   5. Not enrolled in any other interventional study where study intervention/activities may affect outcome measures (patients enrolled in purely observational studies can be included)  **Additional Participant Inclusion Criteria for the nested, platform randomised drug trial**  (to be eligible participants must meet **all above criteria** and all those below)  *Note: Potential participants with drug-specific contraindications for any arm, including interactions of pre-prescribed essential medication will be consented for data collection but will be excluded from the drug study.*   1. Females of childbearing potential must be willing to use an acceptable, effective method of contraception during treatment with the IMP and for 30 days after the last dose. Such methods include: 2. combined (oestrogen and progestogen containing) hormonal contraception: 3. oral 4. intravaginal 5. transdermal 6. progestogen-only hormonal contraception 7. oral 8. injectable 9. implantable 10. intrauterine device (IUD) 11. intrauterine hormone-releasing system (IUS) 12. bilateral tubal occlusion 13. vasectomised partner 14. male or female condom with spermicide 15. cap, diaphragm or sponge with spermicide sexual abstinence (true abstinence)   7. Male participants must be willing to use condom during IMP treatment and for a further 90 days after the last dose of trial IMP to protect their female partner becoming pregnant.  8. Patients on pre-existing treatments for the same drug classes MUST undergo a 7-day washout period before being randomised.  *(Patients will be assessed, and if safe to exclude that medication for 7 days, they will be asked if they would be willing to undergo a washout period of at least 7 days before being randomised.)*  **Participant Exclusion Criteria for ALL Participants**   1. Previously hospitalised for COVID-19 infection. 2. Previously referred to a long COVID clinic.   **Exclusion criteria for nested, adaptive randomised drug trial**  3. Females who are pregnant, planning pregnancy or breastfeeding  4. Known hypersensitivity to any of the study drugs or their excipients  5. Currently taking any of the following drugs:  Probenecid, Sucrafate, Isocarboxazid, Phenylzine, Tranylcypromine of any other CNS depressant (such as diphenhydramine, dextromethorphan, or pseudoephedrine)(Contraindications to famotidine/loratadine)  Amiodarone, Aprepitant, Atanazavir, Atorvostatin, Azithromycin, Bezafibrate, Ciclosporin, Ciprofibrate, Clarithromycin, Cobicistat, Croztibib, Darunavir, Diltiazem, Dronedarone, Eliglustat, Erythromycin, Fenobibrate, Fluconazole, Fluvastatin, Fosamprenavir, Gemfibrozil, Idelalisib, Imatibib, Isavuconazole, Itraconazole, Ketoconazole, Letermovir, Lopinavir, Netupitant, Nilotinib, Posaconazole, Pravastatin, Ranolazine, Ritonavir, Rosuvastatin, Simvastatin, Tipranavir, Velpatasvir, Vemurafenib, Venetoclax, Verapamil, Voriconazole *(Contraindications to colchicine)*  Acalabrutinib, Aceclofenac, Acenocoumarol, Alprostadil, Alteplase, Argatroban, Aspirin, Axitinib, Beniparin, Benzydamine, Bevacizumab, Bismuth, Bivalirudin, Bosutinib, Bromfenac, Cabozantinib, Cangrelor, Caplacizumab, Celecoxib, Cilostazol, Clopidogrel, Cobimetinib, Dabigatran, Dalteparin, Danaparoid, Dasatinib, Dexkeptorofen, Diclofenac, Dipyridamole, Enoxaparin, Epoprostenol, Eptifibatide, Etodolac, Etoricoxib, Flurbiprofen, Heparin, Ibrutanib, Ibuprofen, Iloprost, Imatinib, Indomethacin, Inotersen, Ketoprofen, Ketorolac, Levatinib, Mefenamic acid, Meloxicam, Nabumetone, Naproxen, Nicotinic acid, Nintenanib, Parecoxib, Pazopanib, Phenazone, Phenindione, Piroxicam, Ponatinib, Prasugrel, Regorafenib, Ruxolitinib, Sorafenib, Streptokinase, Sulindac, Sunitinib, Tenecteplase, Tenoxicam, Tiaprofenic acid, Ticagrelor, Tinzaparin, Tirofiban, Tolfenamic acid, Trametinib, Traztuzumab emtansine, Trprostinil, Urokinase, Volanesorsen, Warfarin (Contraindications to Rivaroxaban)  6. No history or presenting symptomology suggestive of Renal failure/insufficiency (eGFR <15ml minute) on the basis of blood investigations (eGFR) within the last 6 months and clinical assessment  7. Severe liver dysfunction on the basis of blood investigations within the last 6 months (liver function and coagulation) and clinical assessment |
| **Statistical Methodology and Analysis** | Primary Outcome – Effectiveness: Fatigue at 12 Weeks (measured with the Fatigue Assessment Scale) analysed on an intention-to-treat basis. An analysis of covariance will be employed to estimate the effects of Coverscan^TM^, digitally enabled community rehabilitation, and their interaction. |

# KEY ROLES AND RESPONISIBILITIES

**SPONSOR**: University College London (UCL) will act as Sponsor for this trial. The sponsor is responsible for ensuring that before the trial begins arrangements are in place for the research team to access resources and support to deliver the research as proposed, and allocate responsibilities for the management, monitoring and reporting of the research. The Sponsor also must be satisfied that there is agreement on appropriate arrangements to record, report and review significant developments as the research proceeds, and approve any modifications to the study design. The Sponsor will maintain oversight of the trial, be involved in all research contract negotiations, and sit on the Trial Management Group. UCL will provide insurance for the clinical trial and undertake to ensure that the above trial is conducted in accordance with the Medicines for Human Use (Clinical Trials) Regulations 2004 (as amended), the UK Policy Framework for Health and Social Care Research 3^rd^ edition 2017 (as amended) and all applicable regulatory requirements. The Sponsor has delegated some of its responsibilities to the Lancashire Clinical Trials Unit.

**Lancashire Clinical Trials Unit** is managing the trial setup, submission and will oversee regulatory approvals for all aspects of the trial and manage the conduct of the STIMULATE-ICP trial in conjunction and or on behalf of the Sponsor. Lancashire Clinical Trials Unit is responsible for the maintenance of the trial related documentation and archiving. Research Agreements and standard service level agreements will be established with all relevant organisations and stored at Lancashire Clinical Trials Unit (CTU) and Joint Research Office (Sponsor) at UCL.

**FUNDER:** The funder is the entity that will provide the funds (financial support) for the conduction of the study. Funders are expected to provide assistance to any enquiry, audit or investigation related to the funded work.

**CHIEF INVESTIGATOR (CI):** The person who takes overall responsibility for the design, conduct and reporting of the trial. If the trial involves researchers, clinicians, and academic personnel at more than once site, the CI takes on the primary responsibility for the suitability of an investigator at any particular site.

**PRINCIPAL INVESTIGATOR (PI):** Individually or as leader of the researchers at a site; ensuring that the study is conducted as per the approved STIMULATE-ICP protocol, and report/notify the relevant parties – this includes the CI of any breaches or incidents related to the trial.

# KEY WORDS

|  | Long COVID, diagnostic, epidemiology, informatics, Health Service Research, Clinical Trials, Coronavirus, Integrated Care Pathway, Long Term Condition, Work Packages |
| --- | --- |

# BACKGROUND

Long COVID (1) spans multiple healthcare challenges, particularly how to deliver sustainable, high-quality care for multimorbidity and long-term conditions (LTC). There are inequalities in health, access to healthcare; incomplete pathways across community and hospital care; inadequate research translation to practice; and insufficient resources. Integrated care pathways (ICP) are structured multidisciplinary care plans of essential steps in care of specific conditions (2). An effective and deliverable ICP for Long COVID could reduce direct and indirect pandemic effects and improve LTC care beyond COVID-19 and potentially be cost effective. Long COVID services were initiated whilst research is still defining the disease. Guidelines are developing in absence of evidence. An ICP approach offers coordination across specialties, across investigation, treatment, and rehabilitation, as well as opportunities for real-time iterative improvements in service design and delivery. ICPs are of increasing relevance in many LTCs. ICPs can improve quality and access to care, patient experience and satisfaction, while reducing complications and non-elective admission rates (3-5). Research and trials of ICPs should incorporate design elements “likely to maximise effectiveness” (5) and engagement of patients, clinicians and health systems to improve healthcare performance (6).

Long COVID is a syndrome, not a single condition. The “syndemic” of COVID-19 (convergence of an infectious disease, under-treated non-communicable diseases, and geographical, and economic determinants of health (7) necessitates ICP strategies, which could inform other LTCs. Predictors of poor acute COVID-19 outcomes are established (8), but effective treatments for Long COVID are not yet defined (9, 10).

The NIHR-funded STIMULATE-ICP project consists of three inter-connected, cross-linked **Work Packages,** which are led and managed by a multidisciplinary team across different organisations from academia, health services and industry.

The work packages are designed to be time-sensitive, iterative, adaptive and effective, utilising learning health systems (health science, clinical evidence, and care) (11), implementing science, (11-13) and complex interventions (14).

Figure 1 is illustrative of the inter-connection between the different work packages for context purposes only.

This protocol details work package 2 which is a pragmatic, multi-centre, cluster-randomised, Phase III trial of an integrated care pathway with a nested, adaptive, open label, randomised drug trial in individuals with Long COVID.

**
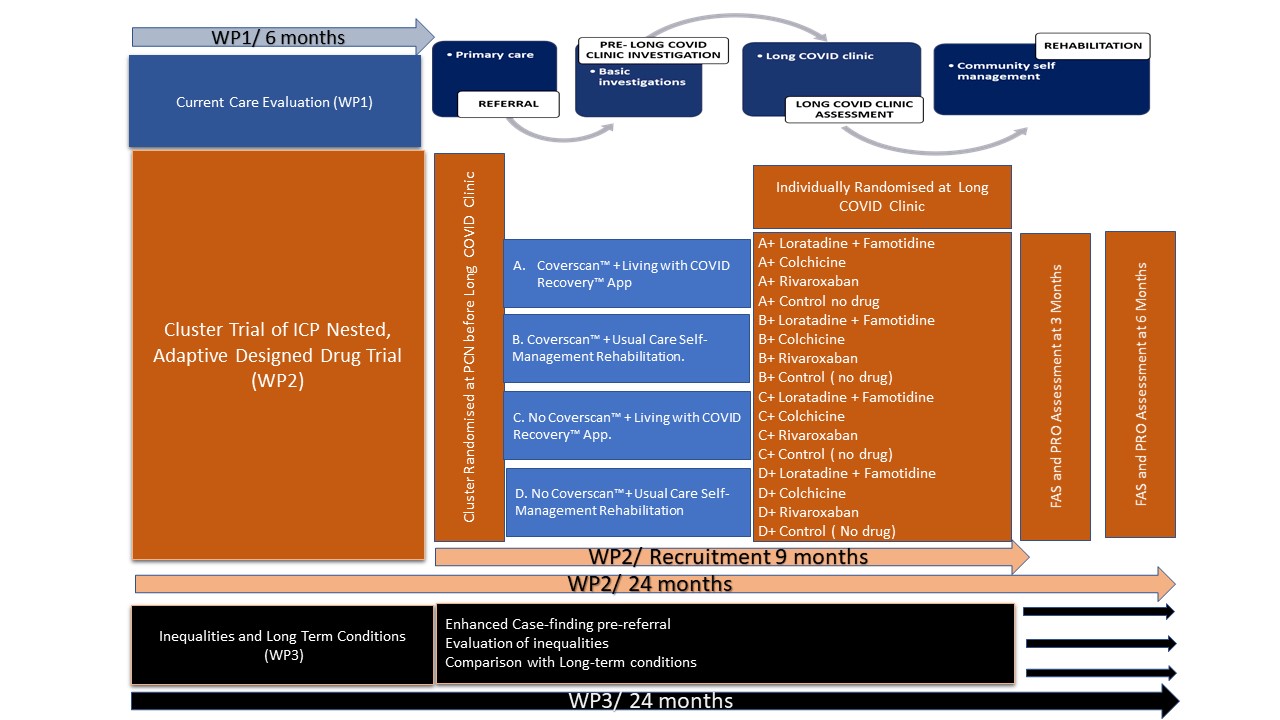
Figure 1: The Schema for STIMULATE-ICP Grant award showing the interconnections between this STIMULATE_ICP Trial and other work packages**

# RATIONALE

There is variation in access to referral and care, and poor patient experience for Long COVID (1). With stretched resources during the pandemic, the need for rationalisation of investigation and rehabilitation across diseases is paramount. In Long COVID, where up to 70% of patients show evidence of mild impairment in one or more organs, (15) an early supported investigation strategy may direct management, as well as better defining the syndrome, with relevance to other LTCs. Despite the primary care setting, there is no evidence-based treatment or rehabilitation. There have been few clinical trials to date, particularly in non-hospitalised individuals, or in the context of overall care pathways. As Long COVID clinics are implemented, trials of ICPs can inform diagnosis, care, public health, policy planning, resource allocation and budgeting.

In the UCLH Long COVID clinic (the first and largest in the UK), community-referred patients were more symptomatic, more likely to have psychological impact, and less likely to be fit for work than patient’s post-hospitalisation. Any ethnic and socioeconomic disparities in referrals, assessment, and rehabilitation (16) need to be understood at a system level and compared with other LTCs, to provide lessons beyond COVID-19, which has worsened pre-existing inequities (7). Pragmatic implementation of effective and equitable interventions is urgently needed to reduce inequalities (17).

Long COVID is estimated to have a substantial population burden, with 2 million people affected in the UK. Even in non-hospitalised individuals, up to 14% of people with COVID-19 may have persistent symptoms (3), suggesting future major healthcare and economic burden (4, 18). We are challenged “to design research that integrates reported lived experience needs with clinical models of care” (6). Guidelines recommend referral to Long COVID clinics if symptoms persist for 4-12 weeks (10). However, neither the syndrome nor effective care is defined (19). Despite roll-out of 83 centrally funded Long COVID clinics, ICPs are not coordinated and the most effective ICP remains unknown because elements of the pathway and their delivery are lacking in evidence. Patients and public want “recognition, research and rehabilitation” simultaneously (14), as well as timely tests and treatment, which may be more deliverable via an ICP.

Real world data needs to be analysed to understand the trajectory of Long COVID, the nature of “usual care” and the effectiveness of current care pathways. These data have already informed our choice of interventions for diagnosis and rehabilitation for a cluster-randomised trial.

Coverscan™ is a multi-organ MRI assessment with exceptional use authorisation by the MHRA for research and clinical use at present (the context of use may change, and this may require protocol amendments to update the context of use) and needs to be evaluated at scale, where there is currently significant variation in investigations pre-Long COVID clinic. Self-management has been the mainstay of rehabilitation in usual care, but more comprehensive, digitally enabled programmes need to be quickly evaluated, such as Living with COVID Recovery^™^. Rather than studying specific components in isolation, given the scale of Long COVID and the urgency of the public health burden, integrated pathways need to be evaluated.

In addition to the components of the ICP, we need a robust evidence-base to inform drug therapies for effective treatment of Long COVID. Some medications are being widely used among patients and health professionals without evidence base.

As a pragmatic trial design based in routine care, it is important to analyse data pre-trial (WP1), data from the trial and demographic and socio-economic data to monitor inequalities (WP3) in the intervention, as well as considering applicability to other LTCs.

## Further Rationale for the Trial and Interventions

Patient surveys, interviews with patients, health professionals and policymakers, and data from UCLH clinic suggest a lack of coordination of care and missed opportunities to improve investigation and rehabilitation. An example of this is repetitive investigations in different modalities for the same organ, and different scans at different times for different organs. One of the Integrated Care components that we will assess is Coverscan™ (15) which is a non-CE marked medical device given MHRA-authorisation for exceptional use in research and clinical care, and has been used in 500 patients. It aligns with national policy for community diagnostics (NHS Diagnostic Hubs (34)). Interviews with clinicians, patients and researchers show support for Coverscan™ to be used as an early investigation strategy, to be evaluated in routine care, and to help stratify treatment.

The second component of Integrated Care to be assessed is “Living with COVID Recovery™” which is an NIHR-funded digital health intervention (NIHR132243), initiated in August 2020 (36), providing videos, information and rehabilitation treatments under guidance of a rehabilitation clinician. The “Living with COVID Recovery™ App is a class I, CE Marked Medical Device. Over 400 patients in 15 of the 83 Long COVID clinics (mean age: 54 years, range 23–78; 50% from ethnic minorities), have used the app to-date. Patients perform seven actions per week such as weight recording, patient reported outcome measures (PROMs), completion of GAD-2, MRC Dyspnoea score or exercise tracking, with half creating and tracking chosen goals. A physiotherapist needs an hour to review each patient, managing 70 individuals per month. Clinicians find it efficient and patient-centred.

## Rationale for the Use of Drugs in STIMULATE-ICP Trial and Underlying Data

To date, little to no treatment data exists for Long COVID; understanding of the pathophysiological mechanisms of disease is poor, and few well-designed clinical trials are currently registered. UCLH hosts the first, and largest of the NHS Long COVID clinics. In a cohort of >1000 patients living with Long COVID at UCLH, the most common reported symptoms are fatigue, shortness of breath and reduced exercise tolerance, similar to reports from other centres (16, 20). Median Fatigue Assessment Scores (FAS) at presentation are >30 in >60% of Long COVID patients (normal range FAS <20) irrespective of time since symptom onset. FAS scores are significantly higher than post-hospitalised patients, similar to other reports (21).

Proposed mechanisms underlying Long COVID symptom clusters include mast cell activation, microcirculatory deficiency caused by microthrombi, and mitochondrial insufficiency. Preliminary observations by our UCLH team suggest Long COVID patients have evidence of persistent inflammation and/or microcirculatory insufficiency, resulting in poor aerobic muscle capacity and profound fatigue. The nested STIMULATE-ICP trial interventions support existing hypotheses and are based on data from UCLH Long COVID clinic patients.

We hypothesise that drug treatments in Long COVID may significantly improve symptoms and physical function, through different mechanisms of action. We have designed an adaptive, platform clinical trial (22-25) within STIMULATE-ICP, to robustly test efficacy of drugs in Long COVID. We will start with Loratadine plus Famotidine, Colchicine and Rivaroxaban and continue to add/remove drugs according to emerging preliminary data in Long COVID and as approved by the Independent Data Monitoring Committee (IDMC), Trial Steering Committee (TSC) and the Funder (NIHR). It is likely that Long COVID represents a heterogenous group of different pathological responses to primary mild to moderate COVID-19 infection, with unknown predisposition. However, since the underlying cause(s) of Long COVID are unknown, we have chosen to test these drugs across all participants with Long COVID, irrespective of their individual symptoms or investigation findings, as we cannot currently determine which approach is optimal to treat the hypothesised underlying inflammatory process. This approach will minimise selection bias and allow multiple secondary analyses to test for efficacy in particular sub-groups of patients, based on symptom clusters or imaging findings.

The large sample size will enable us to undertake multiple pre-specified “symptom phenotypes” analyses based on both Coverscan™ findings and symptom clusters to determine if these sub-groups do benefit from specific drugs or not.

Estimating the individual effect size of these drugs on fatigue is complex, as extrapolating preliminary data to the larger patient cohort is difficult. We hypothesise that Long COVID patients who experience symptoms may gain most benefit from the trial drugs, but in the absence of detailed pathophysiological data for Long COVID, we are open to possibility of differential effects of these drugs in different sub-groups.

Alternative treatment arms may be included/changed during the trial at pre-specified points on the recommendation of the IDMC and TSC, based on advice and involvement from the COVID-19 UK Government Therapeutics Task Force at the Department of Health & Social Care.

## Predicted Efficacy of the Drugs in Long COVID

Long COVID is a new condition, without consensus on the underlying pathophysiology. While a myriad of symptoms is reported by long COVID patients, fatigue is the most frequent symptom in our experience of over 1000 Long COVID patients, and is most commonly reported as the symptom that most limits quality of life (21, 26). Among other symptoms reported are chest pain, cognitive slowing, reported commonly as ‘brain fog’, dyspnoea, headache, dizziness, palpitations, and sleep disturbances.

The drugs selected for use in the STIMULATE-ICP trial are based on emerging data from our service and other Long COVID clinics, patient groups and emerging, early clinical trials.

Using an adaptive platform approach, new drugs may be added to the protocol, following discussions and approval from the NIHR specialist advisory group for Long COVID, through amendment to this protocol.

### Loratadine and Famotidine (H_1_ + H_2_ Receptor Blockade)

Loratadine and Famotidine are both histamine receptor antagonists. In combination they inhibit both the H_1_ and H_2_ receptors. Famotidine is an effective competitive H_2_ receptor antagonist. It reduces the concentration and amount of acid and pepsin of the gastric juices. The effect of oral administration is rapid, long lasting when used at the recommended dosage and it is effective with relatively low concentration in the blood. The duration of its effect, plasma concentration and secretion in the urine are dose-dependent. Famotidine is licensed as an over the counter (OTC) treatment for dyspepsia/gastric ulceration. Loratadine is also an OTC treatment licensed for treatment of mild allergic symptoms including seasonal rhinitis.

Patients with Long COVID are hypothesised to have a persistent inflammatory process that may drive the symptoms of fatigue and myalgia. In a small minority of patients, more specific symptoms suggestive of mast-cell activation, including rashes, diarrhoea and flushing may be present. Histamine receptor antagonists are commonly used to treat mast-cell activation in other conditions.

Mixed reports of efficacy of histamine receptor antagonists in acute COVID-19, particularly reduction in oxygen requirements and possibly mortality, have led to ongoing clinical trials of these treatments in acute COVID-19 (27). The suggested mechanisms of action in acute COVID-19 are reduction in inflammation due to suppression of T-cell mediated cytokine release, however, available data are very limited. In an observational cohort study, 49 patients with Long COVID were compared to matched asymptomatic fully recovered COVID-19 patients without Long COVID. Long COVID patients were offered treatment with combined histamine H1/H2 blockade, using H1 (Loratadine 10 mg once daily or Fexofenadine 180 mg twice daily (not being used in this study) and H_2_ (Famotidine 40 mg once daily or Nizatidine 300 mg once daily (not being used in this study)) for a minimum of 4 weeks (28) , based on data from acute COVID-19 suggesting histamine receptor antagonist therapy improved symptoms (29, 30). In this preliminary observational study, patients with physician-diagnosed Long COVID reported a 48% reduction in symptom burden after 4 weeks of combined histamine receptor antagonist therapy, compared to baseline. When compared to recovered non-hospitalised patients, patients with Long COVID had significantly lower circulating CD4 but not CD8 effector memory cells, suggesting that this T cell subset may be involved in Long COVID pathology (28). These data, however, have significant limitations, and the findings require testing in a formal clinical trial.

In this study we will be using famotidine 40mg once daily in combination with loratadine 10mg OD, ensuring combined histamine receptor antagonist activity across the H_1_ and H_2_ receptors. Both drugs are being used within their licensed dose and are safe to be co-administered.

### Colchicine

Colchicine inhibits cellular transport and mitosis by binding to tubulin and preventing its polymerisation as part of the cytoskeleton transport system. Colchicine has a short half-life of 9-12 hours and is prescribed as a BD (twice daily) dosing regimen. Standard doses for acute gout range from 500mcg BD to 2mg BD, depending on the dose response. Colchicine has a wide range of anti-inflammatory effects, including inhibition of certain inflammasomes (cytosolic pattern recognition receptor systems that are activated in response to detection of pathogens in the cytosol) (31), (32). Evidence shows that inflammasomes are activated in COVID-19, and the degree of activation is correlated with disease severity ( (33)).

Colchicine has been shown to have cardiovascular benefit in patients with coronary artery disease and pericarditis at a dose of 500mcg BD. Its primary mechanism of action is reduction of serositis, inflammation of membranes around joints and viscera. Patients with Long COVID frequently complain of symptoms suggestive of serositis, either atypical chest pain that may indicate pericarditis, costochondritis or pleural inflammation, or joint pain in the absence of clinically evident inflammation. Disease severity in Long COVID correlates with myocardial damage on Coverscan™ (15). Of the non-hospitalised patients with Long COVID at UCLH, 20% report chest pain and or palpitations, 60% report shortness of breath. Standard investigations are frequently normal: prolonged rhythm monitoring typically shows resting sinus tachycardia, chest pain is generally atypical with predominantly normal echocardiogram, Troponin T and ECG. In a prospective cohort of over 50 patients (median age 43, 69% female) reporting persistent chest pain underwent cardiac MRI (CMR). 26% had evidence of myocarditis-pattern late gadolinium enhancement and/or evidence of abnormalities, (meeting diagnostic criteria for myocarditis (34). T1/T2 abnormalities were seen in 29%. Long COVID patients with myocarditis contrasted to patients admitted to hospital with moderate/severe respiratory COVID (mean age 64, 66% male). However, the prevalence of myocarditis is strikingly similar (35). To date, we have treated in excess of 200 Long COVID patients with abnormal CMR empirically with colchicine 500mcg BD, 9/10 reported significant symptomatic improvement within 3 months.

### Rivaroxaban (Low Dose Anticoagulation)

Rivaroxaban is an oral factor Xa inhibitor, inhibiting the clotting cascade. Patients with Long COVID who complain of marked exertional fatigue with abnormalities on the 6-minute walk test, have evidence of microvascular anaerobic respiration, despite normal oxygenation in peripheral blood. This suggests that while adequate haemoglobin-bound oxygen is present in blood, the dissociated oxygen is unavailable to large muscles on increased aerobic demand during exercise. Abnormalities of the clotting cascade are a marked feature of acute Covid-19, including microvascular thrombi (36). Thrombi in acute Covid-19 are strongly associated with abnormally elevated von-Willebrand Factor (VWF): ag/ADAMTS 13 ratio (37). Recent data are showing longer term risk of VTE with Long COVID. We extended the measurement of VWF Ag/ADAMTS 13 levels to the UCLH community Long COVID patient cohort (Scully and Heightman, 2021. Unpublished data.) Of 272 patients in Long COVID clinic describing extreme lethargy, headaches and poor exercise tolerance, 81/272 (30%) had an abnormal VWF Ag/ADAMTS 13 ratio of >1.5. Elevated VWF Ag/ADAMTS 13 ratio strongly associated with impaired exercise capacity on a 6-minute walk test: 1.5 compared to 1.1 in patients with normal exercise capacity (p<0.001).

A further 28 patients had blood analysed by flow chamber assay, which measures VWF, platelet binding and in-vitro thrombus formation in real time. 8/28 (29%) had clot formation by 5 minutes. In a small feasibility project, 5 patients were initiated on low dose aspirin (LDA) 75mg daily, selected based on symptom severity and elevated VWF(Ag):ADAMTS13 ratio. In 3/5 patients, improvement in symptoms and VWF(Ag):ADAMTS13 ratio was observed. The remaining two stopped aspirin due to upcoming procedure and bruising. Patients B and C were analysed on the flow chamber pre- and post-aspirin usage with marked improvement in surface coverage from 100% to 29% and 9% respectively on LDA, reporting corresponding symptom improvement.

Evidence of microvascular thrombi in some patients has led to the hypothesis that that endothelial dysfunction due to multiple microvascular thrombi in large muscles may significantly contribute to reduced aerobic capacity and symptoms of fatigue. Low-dose anticoagulation is a safe approach to test this hypothesis, by measuring fatigue as the primary outcome, we will determine if this approach indicates the presence of microvascular thrombi in Long COVID patients. Aspirin was poorly tolerated when tested in our pilot study due to gastro-intestinal side effects. We have included rivaroxaban 10mg once daily in the STIMULATE-ICP trial instead of aspirin due to improved safety profile, easy comparability with other clinical trials using factor Xa inhibitors such as apixaban (HEAL-COVID) and proven efficacy data on prophylaxis of intravascular thrombi. The rivaroxaban regime of 10mg OD proposed in STIMULATE-ICP is a prophylaxis dose, which aims to effectively prevent generation of further microvascular thrombi, while minimising bleeding side effects. This prophylaxis dose is therapeutically equivalent to low-dose aspirin tested in our preliminary cohort.

# RISK ASSESSMENT AND MANAGEMENT OF RISKS

## Risks Associated with Patient Population in the Trial

Patients with Long COVID Syndrome generally have poor prognosis for underlying health status, with few medical co-morbidities. Multi-morbid patients, or those with severe complications from their initial COVID illness (including pulmonary fibrosis) are at risk of increased adverse reactions to the study drugs.

Severely ill patients will be closely monitored by their treating physicians and the protocol accepts the requirement for unscheduled visits according to the clinical needs of the patients. Adverse events and biochemical abnormalities will be documented on a continuous basis the reporting procedure will be followed according to the Protocol and the Sponsor (UCL) SOP.

The research team will aim to recruit largely from the local population in the 6-10 designated geographical areas, to enable participants to travel to the clinics easily accessible to them. The recruitment period has allowed for these factors to be considered. Severely ill patients travelling from long distances may not be able to adhere to the scheduled visit but remain in the trial for the purpose of follow-up and remote assessments. A small percentage may withdraw. The trial team anticipate recruiting approximately 100 patients per month per area. The recruitment, the eligibility criteria and concomitant medications will be monitored and documented regularly (at least every 4 weeks in the first 24 Weeks of the trial).

In some areas, due to the design of the clinics, recruitment may need to be centralised to support eligibility screening, consenting, collection and processing of research bloods, and delivery of Living with COVID Recovery ^TM^ digital App. A full risk assessment will be carried out for this trial; mitigations will be provided before the centres are activated and patients are recruited as part of the Sponsorship Green Light approval.

## Risks Associated with non-IMP (Intervention) Component of the Trial

**Coverscan**™ is a multi-organ MRI scan for Long COVID. The scan has received Emergency Use Authorisation from the MHRA for the indication of investigation of Long COVID (23). The benefit of Coverscan™ is that it will provide a multi-organ assessment in individuals with Long COVID, which may help to map the trajectory of disease and to predict progression, recovery and/or response to potential treatments or rehabilitation.

The table below summarises the risks and mitigations of all non-IMP tests and/or procedures above Pre-ICP standard of care that are being performed:

**Table 1: Summary of the risks and mitigations of all non-IMP tests and /or procedures**

| Intervention | Potential risk | Risk Management |
| --- | --- | --- |
| Coverscan™: MRI is a safe and non-invasive technique with minimal risk to the patient. It does not involve ionising radiation or contrast media injection. | In body ferromagnetic objects or implanted devices can be damaged by the MRI magnet or can cause patient injury. | On receipt of clinic referral, staff will screen the patient record for any obvious exclusion factors. Participants will receive an appointment letter outlining any exclusions and be asked to contact staff if they have any queries. Before entering the scanner room, all participants will be screened for any exclusion factors by the scanning radiographer and patients will confirm the assessment in writing. |
|  | MRI can be noisy. | Patients will be provided with headphones and earplugs to reduce noise. |
|  | Patients are required to remain still in a confined space and may feel anxious or the scan may have to be stopped due to patients becoming claustrophobic. | Participants will always be in communication with the scanning radiographer and may choose to end the scan at any time. |
|  | While the risk is minimal, given the target population’s post-infection circulating antibodies and the general immunisation status, the scanner room is a confined space and there is a risk of COVID-19 infection. | Adequate cleaning protocols between patient scans will minimise any risk of transmission and symptom screening will be done prior to scanning. |
|  | Participants will be asked to not eat or drink for 3 hours prior to their MRI scans, but will be able to drink and eat after the scan. | To minimise any risk of infection, participants will be reminded to come equipped with a snack to consume after their MRI scan. |
| Living with COVID Recovery™ | No known potential risks. | Any reports as to adverse effects of using the app, will be monitored and reported to the site and TMG and Living with C0VID Recovery™ manufacturer |

## Safety Profile of the Trial IMPs

All the drugs used in this trial are licenced by the UK regulator (MHRA) for their therapeutic indications but not for Long COVID Syndrome. In accordance with the MRC/DH/MHRA Joint Project Risk-adapted Approaches to the Management of Clinical Trials of Investigational Medicinal Products, this trial is categorised as **Type B = Somewhat higher than the risk of standard medical care.**

Patient medical records and past medical history will be examined for comorbidities that may interfere with the pharmacodynamic and bioavailability of the trial drugs. The exclusion of certain medical conditions will be implemented as part of the eligibility criteria to protect the safety of the participants. The enrolment of participants will be based on the inclusion and exclusion criteria, taking into account comorbidities, outcome of physical examination, laboratory investigations, functional tests and current drug prescriptions as part of standard care.

Screening of medical history, comorbidities and recorded clinic and GP ordered test results will mitigate against the risks posed by these drugs and reduce the burden of unwanted side effects of the trial IMPs.

Prior to randomisation all participants will have their clinic referral and clinic notes checked for comorbidities and concomitant medications to exclude conditions that might cause overdose or side effects relating to higher bioavailability or reduced clearance of the drugs. Their existing medications will be checked for contraindications to each of the drugs on the trial. Their pre-existing laboratory results will be examined for abnormalities that might potentiate the effects of the IMPs or their side effects. Important safety measures are Full Blood Count (FBC) within last 12 months, and no history or presenting symptoms to suggest renal or hepatic impairment. Any abnormalities of tests or suggestive symptomology will need to be considered carefully by the site PI and treating clinician (if different) and definitive tests ordered as appropriate before considering the participant for inclusion in the drug trial. Patients will be given a wallet card containing an emergency contact number and the details of the trial drugs in case urgent medical attention is needed. Common side effects and contact details of the trial team will be included in the Participant Information Sheet (PIS). Patients experiencing adverse reactions will be sign posted and advised to seek medical help from their General Practitioner (GP)s, or treating physicians if they are available, and will be treated as per Standard of Care treatment available on NHS.

### Safety Profile of Loratadine

Loratadine is a long-acting, non-sedating tricyclic antihistamine (piperidine derivative) with selective antagonistic properties to peripheral histamine H_1_-receptors. Loratadine selectively inhibits H_1_-receptors primarily located on respiratory smooth muscle cells, vascular endothelial cells, the gastrointestinal tract, and immune cells. Loratadine has a long-established safety record and is widely available over the counter as well as by prescription.

At the recommended dose of 10mg daily, adverse reactions with Loratadine were reported in 2% of patients in excess of those treated with placebo. The most frequent adverse reactions reported were somnolence (1.2%), headache (0.6%), increased appetite (0.5%) and insomnia (0.1%), according to the SmPC: Clarityn Allergy (loratadine) 10mg Tablets (Bayer plc).

Loratadine should be administered with caution and at reduced doses in patients with severe liver impairment because they may have reduced clearance of loratadine. Therefore, patients with severe liver impairment will be excluded from the trial.

Participants, before entering the trial and before issued with a new prescription for the trial drugs, will be screened for contraindicated medications, in particular for concomitant medications with other CNS depressants such as diphenhydramine, dextromethorphan, or pseudoephedrine as these may cause dose-related sedation. Patients will be advised to use caution when driving or operating heavy machinery, as it may cause drowsiness. Patients with Chronic Obstructive Pulmonary Diseases may experience flare-up of the condition due to its anticholinergic effects.

### Safety Profile of Famotidine

Famotidine is a competitive histamine H_2_-receptor antagonist that binds to the histamine receptors located on the basolateral membrane of the parietal cell in the stomach, effectively blocking histamine actions. It decreases the production of stomach acid and is used in the treatment of acid-related gastrointestinal conditions. Famotidine is available both by prescription and over the counter.

Most common adverse effects include agitation in less than 1%, headache (5%), dizziness (1%), diarrhoea (2%), and constipation (1%), according to the SmPC: SmPC Famotidine 40mg Tablets (Tillomed Laboratories Ltd).

Famotidine is secreted mainly via the kidneys, caution should be exercised when treating patients with renal insufficiency. Patients with creatinine clearance (eGFR) below 15 ml/min will be excluded from the study.

Patients will be informed to notify the research team and their treating physician if they experience frequent chest pain and frequent wheezing (atrioventricular block is very rare but serious side effect), unexplained weight loss, stomach pain, heartburn for a duration exceeding more than 12 weeks (Famotidine could mask the symptoms of underlying malignancy, therefore, underlying malignancy must be excluded), heartburn with light-headedness, sweating, or dizziness. These symptoms will stop with discontinuation of the trial drug.

### Safety Profile of Colchicine

**Low-dose colchicine** has been used safely for many years for the prevention of inflammatory complications of Familial Mediterranean Fever and Bechet’s disease. In contrast to the higher doses of colchicine used for treatment of acute gout, low dose (0.5mg to 1mg/day) colchicine used in recent cardiovascular trials (CORE) [64] shows excellent safety profile and the frequency of side effects were less than 2%.

According to the SmPC: SmPC Colchicine Tablets BP 500 mcg (Wockhardt UK Ltd), the side effects associated with using Colchicine are highly dose dependent, infrequent, and usually stops following reductions in the dosage or complete cessation of the treatment. Of note, Colchicine is genotoxic (in vitro and in vivo) and teratogenic in animal studies, and contraindicated in pregnant females. However, a 2018 metanalysis of 4 trials concluded that in pregnant and breastfeeding women prescribed colchicine, no increased incidence of foetal malformations or miscarriage was noted (38). All participants of child-bearing potential enrolled in STIMULATE-ICP will be required to use an acceptable, effective method of contraception (see section 7.2 and Appendix II for definition of acceptable effective methods of contraception for this trial) for up to 30 days after treatment discontinuation and females of childbearing potential will undertake a pregnancy test prior to enrolment . Male participants will be required to use a condoms to protect their female partner becoming pregnant for up to 90 days after last dose of IMP .

Colchicine is significantly excreted in urine in healthy subjects. Clearance of colchicine is decreased in patients with impaired renal function, therefore patients with a creatinine clearance (eGFR) below 15 ml/min will be excluded from the study.

Grapefruit juice may increase plasma levels of colchicine. Grapefruit juice should therefore not be taken together with colchicine.

### Safety Profile of Rivaroxaban

Patients receiving Rivaroxaban do not require regular monitoring at the recommended dose of 10mg per day. Rivaroxaban is highly protein-bound. Participants randomised to the drug trial will have a blood test for platelet count, activated Partial Prothrombin time, Prothrombin time and bleeding time.

Rivaroxaban is an anticoagulant, and as such the most common complication associated with Rivaroxaban treatment is bleeding. Risk factors for bleeding include bacterial endocarditis, underlying congenital or acquired bleeding disorders, vascular retinopathy, thrombocytopenia, recent procedure/surgery, stroke, neuraxial procedures, uncontrolled hypertension, renal impairment (patients with a creatinine clearance (eGFR) below 15ml/min will be excluded from the study), recent major bleeding, concomitant use of other drugs that affect haemostasis, and advanced age. Clinical evaluation is necessary for any unexplained decrease in haemoglobin or blood pressure.

Other complications are associated with central nervous system, skin, gastrointestinal and neuromuscular systems are rare with frequencies ranging from 1 to 3%:

A small increased risk of thrombosis is present with premature cessation of Rivaroxaban so, when discontinuing the drug for reasons besides pathological bleeding or completing the course of therapy, the addition of alternative anticoagulant therapy should merit consideration by the treating physician.

Patients on this arm of the trial will be informed to seek help if they experience unexplained weight loss, feeling faint or high blood pressure. The participants would be able to contact the trial team for advice who will sign post them to the nearest emergency department if urgent medical attention is required.

In addition, reproductive toxicity and embryo-foetal toxicity has been shown in animal studies of rivaroxaban which is contraindicated in pregnant females. All participants of child-bearing potential enrolled in STIMULATE-ICP will be required to use acceptable effective method of contraception (see section 7.2 and Appendix II for definitions of acceptable effective contraceptive methods) and females of childbearing potential will undertake a pregnancy test prior to enrolment.

**Section 6.8** provides full detail on drug interactions, specific contraindications and mitigations related to the trial IMPs.

**Appendix I** provides a summary of risks associated with the drugs used in the trial and management of the risks. The full list of adverse reactions associated with the above-mentioned IMPs are provided in the appropriate SmPCs.

# OBJECTIVES AND OUTCOME MEASURES/ENDPOINTS

## Primary Objectives

The overall primary objective is to evaluate the effect of “integrated care” with combinations of multi-organ MRI (Coverscan™) and clinical decision support, and digitally enabled community rehabilitation (Living with COVID Recovery™) versus “usual care” [Usual Investigations; and Your COVID Recovery™ self-management website] on Fatigue Assessment Score (FAS) at 12 Weeks from the enrolment date.

## Secondary Objectives

The secondary objectives include:

1. Clinical efficacy of the individual components of the integrated care pathway, including potential therapies.
2. Effect of the ICP on mean measured health related quality of life, mental health (including depression), work and social adjustment, physical function, organ impairment and healthcare utilisation through patient reported outcomes.
3. To characterise the pathophysiology and trajectory of long COVID

## Primary Outcome Measure

Fatigue is the dominant symptom in 60% of patients and Long COVID clinics are being commissioned to provide rehabilitation for 12 weeks or less, therefore, a 12-week outcome is a pragmatic primary outcome measure.

Fatigue Assessment Scale is a 10-item, validated questionnaire in patients with chronic diseases. Therefore, it is a pragmatic primary end point for the STIMULATE-ICP trial. Five questions reflect physical fatigue and five questions (questions 3 and 6-9) reflect mental fatigue. The total score ranges from 10 to 50.

## Secondary Outcome Measures

Secondary outcomes will include:

1. Health related quality of life (EQ-5D-5L)
2. Mental health (GAD-7)
3. Depression (PHQ-9)
4. Medical Research Council Dyspnoea Score
5. Perceived Deficit Questionnaire (PDQ-5)
6. Work and Social Adjustment Scale (WSAS) [ Question 4 from Productivity Cost Questionnaire (iPCQ) for absenteeism and Question 8 from iPCQ for presenteeism added]
7. Short Form Questionnaire (SF-12)
8. Cognitive Failure Questionnaire (CFQ) if a patient scores 3 or more on PDQ5 (patients receive an email to complete this questionnaire online via a secure password and patient ID number)
9. Organ impairment and healthcare utilisation
10. Cost-effectiveness of ICP
11. Process outcomes for different ICP components
12. Blood investigations (e.g., genomic, proteomic, metabolomic/ lipidomic, functional T cell and live virus neutralisation assays and endocrine analyses)
13. Linked electronic health record data to monitor healthcare utilisation and outcomes.

A summary of the objectives and outcome measures are presented in Table 2 below:

**Table 2: Summary of the primary and secondary objectives and outcome measures**

| **Objectives** | **Outcome Measures** | **Timepoint(s) of evaluation of this outcome measure** |
| --- | --- | --- |
| **Primary Objective** Evaluation of the effect of “integrated care” with combinations of multi-organ MRI (Coverscan™) and clinical decision support, and digitally enabled community rehabilitation (Living with COVID Recovery™) versus “usual care” [Usual Investigations; and Your COVID Recovery ^TM^ self-management website] on Fatigue Assessment Score at 12 Weeks from the date of enrolment in the trial. | FAS | Baseline, 12 and 24 weeks |
| **Secondary Objectives** The overall clinical efficacy of the individual components of the integrated care pathway including potential therapies.   1. Effect of the ICP on mean measured health related quality of life, mental health (including depression), work and social adjustment, physical function, organ impairment and healthcare utilisation, through patient reported outcomes. 2. To determine if pre-specified sub-groups of patients, either grouped by clinical symptom cluster or imaging findings benefit from either early investigation with COVERSCAN™, IMPs or rehabilitation. | 1. Health related quality of life (EQ-5D-5L) 2. Mental health (GAD-7) 3. Depression (PHQ-9) 4. MRC Dyspnoea Score 5. Perceived Deficit Questionnaire (PDQ-5) 6. Work and Social Adjustment Scale (WSAS) [ Question 4 from Productivity Cost Questionnaire (iPCQ) for absenteeism and Question 8 from iPCQ for presenteeism added] 7. Short Form Questionnaire (SF-12) 8. Cognitive Failure Questionnaire (CFQ) if a patient scores 3 or more on PDQ5 (patients receive an email to complete this questionnaire online via a secure password and patient ID number) 9. Organ impairment and healthcare utilisation 10. Cost-effectiveness of ICP 11. Process outcomes for different ICP components | Baseline, 12 and 24 weeks |
| 1. To characterise the pathophysiology and trajectory of long COVID | Blood investigations (e.g., genomic, proteomic, metabolomic/ lipidomic, functional T cell and live virus neutralisation assays and endocrine analysis) | Baseline |
|  | 1. Linked electronic health record data to monitor healthcare utilisation and outcomes. | 12 months |

## Exploratory Outcome Measures / Endpoints

There is ongoing research iteratively developing a core outcome data set for clinical and research use in individuals with Long COVID. For example, based on a NIHR-funded research programme (Long COVID Core Outcome Set, LC-COS) which conducted an extensive, international Delphi process in over 1500 patients and health professionals internationally, we have included consensus physiological/clinical, life impact and recovery domains in our trial. We will continue to work with LC-COS in their work to iteratively and pragmatically develop core outcomes for Long COVID as our trial progresses [unpublished communication https://www.comet-initiative.org/Studies/Details/1847 .

# TRIAL DESIGN

The STIMULATE-ICP trial is a pragmatic, multi-centre, cluster-randomised trial of an integrated care pathway with a nested, Phase III, open label, adaptive platform randomised drug trial in individuals with Long COVID

The STIMULATE-ICP trial is testing the hypothesis that an Integrated Care Pathway for Long COVID is more effective than Usual Care at improving the most common symptom of fatigue.

Currently, patients with signs and symptoms of Long COVID undergo usual investigations by their primary care physicians before they are referred to Long COVID clinics where they undergo further investigations and clinical decision support.

## Definitions

STIMULATE-ICP trial is a pragmatic, multi-centre, cluster-randomised trial of an integrated care pathway with a nested, open label, adaptive platform randomised drug trial in individuals with Long COVID.

“Integrated Care” is defined as:

- 1. Community based multi-organ MRI (Coverscan™) and clinical decision support; and
  2. Enhanced community based, digitally enabled, rehabilitation “Living with COVID Recovery™” App

“Usual Care” is defined as:

- 1. Usual investigations (includes blood tests, ECG, Chest X ray and Exercise Tolerance Test); and
  2. Self-management rehabilitation with associated online resources; (Your COVID Recovery™) for rehabilitation; https:\\www.yourcovidrecovery.nhs.uk

In this trial, two integrated care pathway interventions will be cluster randomised at the Primary Care Network (PCN) level, the two innovations are Coverscan™ and Living with COVID Recovery™.

This is a pragmatic randomised clinical trial (RCT) to compare new ICPs to usual care and we need to ensure that any research activities do not impact/change workload for already over-stretched NHS resources, whilst ensuring equity of access to patient groups.

## Justification for Cluster Randomisation

If using Individual Patient Randomisation (IPR), provision of information for the scan, time to consider, and consent would need to take place at the General Practitioner (GP) level. Given the current strain on services this is not feasible and could potentially increase waiting times for other practice patients, affect number of patients approached and recruitment would be unlikely to achieve sample size required within the trial timeline. Similarly, if IPR is used for Living with COVID Recovery^™^ at the GP level the same barriers to recruitment occur.

IPR would mean patients have a 50/50 chance of receiving a scan and a 50/50 chance of being allocated to Living with COVID Recovery™. Cluster randomising at the PCN level will have the same allocation of access across the total number of patients referred to the participating clinic so that equipoise exists at the clinic level. Clustering will use demographic and socio-economic data available for each PCN to ensure equity of access across deprivation indexes within GP codes. Clustering at the PCN level allows Coverscan™ and/or Living with COVID Recovery™ to be provided as an uplift to previous care in allocated PCNs. It will become integrated care within that PCN and, as such, individual patient consent will not be sought (access to and collection of Coverscan™ and Living with COVID Recovery™ app data for research purposes will be by individual patient consent on entering into the study).

Cluster randomisation using a two-by-two factorial design will give us 4 groups:

1. Coverscan™ + Living with COVID Recovery™ App.
2. Coverscan™ + Usual Care Self-Management Rehabilitation.
3. No Coverscan™ + Living with COVID Recovery™ App.
4. No Coverscan™+ Usual Care Self- Management Rehabilitation.

Patients within c and d cluster of (No Coverscan™) are receiving basic investigation as standard of care (see section 5.1) with either Integrated care of the digital community-based Living with COVID Recovery^TM^ App (c) or with community based self-management with the online resources (Your COVID Recovery^TM^) which is a NHS supported online resources for patients recovering from COVID-19.

## Design, Implementation and Evaluation of Integrated Care

This trial aims to investigate two components of care:

### To Evaluate “Integrated Care” versus “Usual Care”

“Integrated Care” involves:

1. Multi-organ MRI (Coverscan™) and clinical decision support; and/or
2. Enabling a community-based rehabilitation using a digital Application Platform (Living with COVID Recovery™).

“Usual Care” involves:

1. Usual Investigations; and
2. Self- management website with associated online resources (*Your COVID Recovery™)* for rehabilitation (39).

A four-arm, two-by- two factorial, cluster-randomised, controlled pragmatic trial of ICPs for Long COVID which is *adaptive* to local resources and needs, *reactive* to changing pandemic and policy environments and *iterative*, developed upon ongoing results; plus an adaptive platform open label, individual patient randomised clinical trial to evaluate 12 Weeks treatment of four potential therapies: H_1_/H_2_ receptor blockade with Loratadine 10mg once daily plus Famotidine 40 mg once daily or Colchicine 500mcg twice daily or Rivaroxaban 10mg once daily vs usual care (4 arms).

Eligible patients will be approached to consent to a **nested, adaptive platform randomised drug trial.** All patients will be sent an invitation letter to the trial with the trial Participant Information Sheet and Consent Form prior to their Long COVID Clinic appointment. The patients may contact the trial team prior to their Long COVID clinic appointment or at the clinic appointment or up to 7-10 days* after their clinic appointment to participate. Consent will be taken at any of these time points but randomisation to the nested, adaptive platform randomised drug trial will only occur at the clinic appointment or in the 7-10 days after the clinic appointment. No randomisation will occur until eligibility for the nested drug trial has been confirmed by the clinical assessment and patient history. Patients who are ineligible or opt not to participate in the drug trial will be asked to consider consent for data collection or data collection plus blood samples and donation to biobank.

* We will not enrol participants for the drug arm after this time frame, so as to align the taking of IMP as far as possible with the 12-week clinic pathway and the 12-week follow-up. 12-week follow-up is based on date of first clinic appointment and commencement of clinical pathway

Eligible patients will be randomised to one of the following 4 arms:

1. Famotidine + Loratadine + usual care
2. Colchicine + usual care
3. Rivaroxaban + usual care
4. Usual care (No Drug)

All patients will be assessed at 12 Weeks and 24 Weeks for Fatigue Assessment Scale.

Other drugs may be added as additional or replacement arms for new patients at predetermined time points during the trial (6 months and 12 months), dependent on interim analysis of trial data with consideration given to updated recommendations from the NIHR advisory board.

Through a cluster randomised trial and a nested adaptive drug trial, evaluation of the Integrated Care versus Usual Care will be conducted by applying validated methods for investigating complex interventions and health services.

**Figure 2: STIMULATE-ICP Flow Chart**


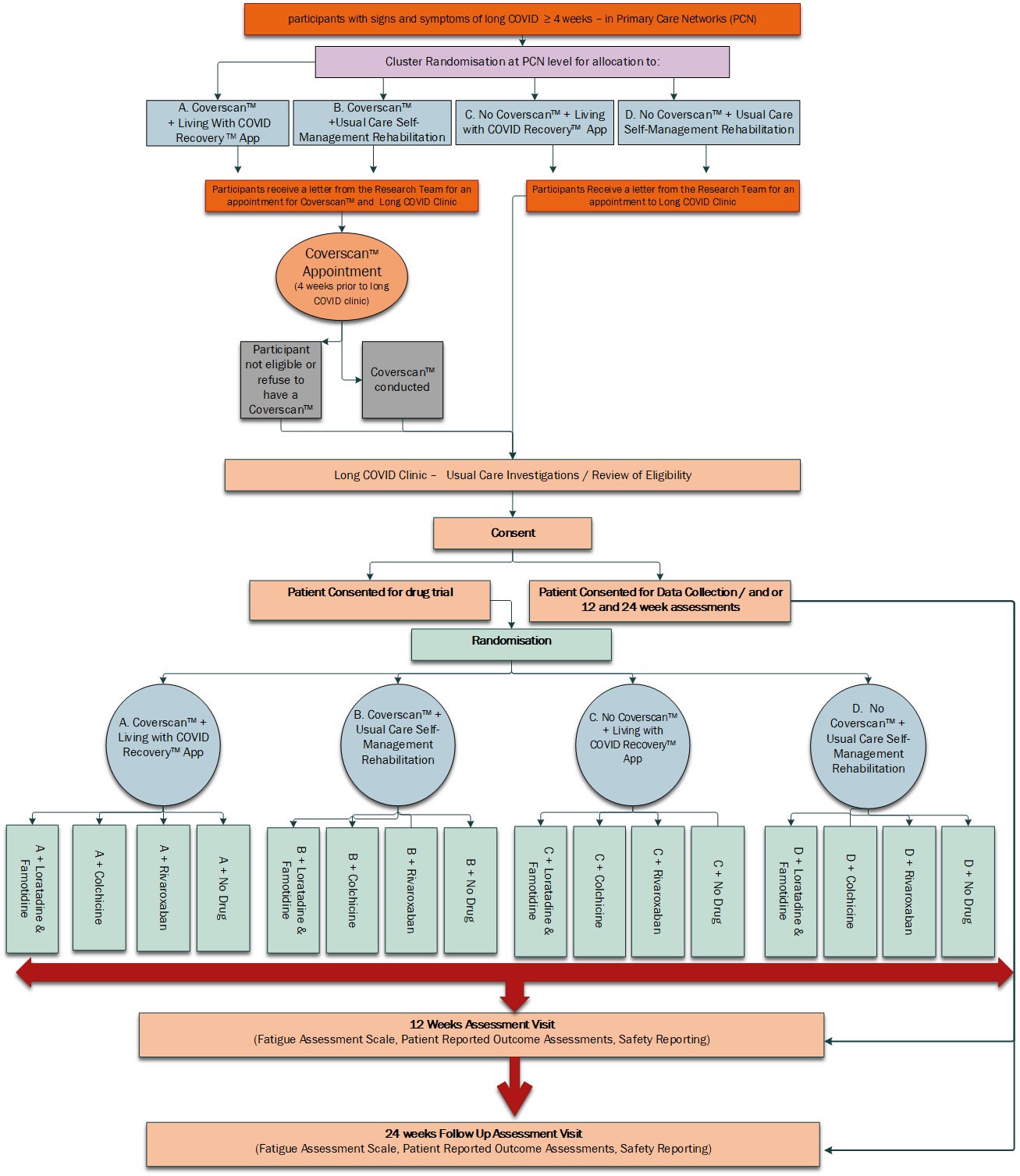


##

## Trial setting

STIMULATE-ICP is a multi-centre trial involving a combination of Primary Care Networks and Long COVID Clinics based in 6-10 different geographical areas of England. The trial is managed by Lancashire Clinical Trials Unit (LCTU) on behalf of the Sponsor. The spread of the sites will be purposeful to encompass geographic and demographically diverse areas. We anticipate 6 -10 sites will be needed dependant on throughput and size of area served.

Lancashire CTU (on behalf of the Sponsor) will ensure all relevant approvals and support is in place prior to activation of study sites.

# INVESTIGATIONAL MEDICINAL PRODUCTS

The four IMPs to be used for the STIMULATE-ICP trial are licensed for therapeutic use in other conditions and will be used off licence in this study. All aspects of supply, storage and management will be in accordance with standard local policy and practice for prescription medications.

## Name and Description of IMP(s)

### Loratadine 10mg tablets taken once daily by mouth

**Therapeutic Properties and Licensed indications of Loratadine**

Loratadine (10mg tablets) is indicated for the symptomatic treatment of allergic rhinitis and chronic idiopathic urticaria. In STIMULATE-ICP, Loratadine will be used outside of its licensed indication but within the licensed dose.

### Famotidine 40 mg tablet taken once daily by mouth

**Therapeutic Properties and Licensed indications of Famotidine**

Famotidine is licensed for treatment of duodenal and benign gastric ulcers which have been confirmed by radiological or endoscopic examination, Zollinger-Ellison syndrome, reflux oesophagitis confirmed by endoscopy, including curative treatment of erosion or ulcer associated with reflux oesophagitis. In STIMULATE-ICP, Famotidine will be used outside of its licensed indication but within the licensed dose.

### Colchicine 500mcg tablet taken twice daily by mouth

**Therapeutic Properties and licensed indications of Colchicine**

Colchicine is licensed and has been widely used for treatment of acute gout and prophylaxis of gout attack during therapy initiation with allopurinol and uricosuric drugs. In STIMULATE-ICP, Colchicine will be used outside of its licensed indication but within the licensed dose.

### Rivaroxaban film coated 10mg tablet taken once daily by mouth

**Therapeutic Properties and licensed indications of Rivaroxaban:**

Rivaroxaban is licensed for Prevention of venous thromboembolism (VTE) in adult patients undergoing elective hip or knee replacement surgery, treatment of deep vein thrombosis, pulmonary embolism (PE) and prevention of recurrent Deep Vein Thrombosis and PE in adults, and stroke prevention in atrial fibrillation. In STIMULATE-ICP, Rivaroxaban will be used outside of its licensed indication but within the licensed prophylaxis dose.

## Formulation, Packaging and labelling of the IMPs

Loratadine , Famotidine, Colchicine and Rivaroxaban will be supplied centrally (details provided in the summary of drug arrangements).. IMPs will be supplied to participating sites in their original packaging and repackaging will not be required.

The IMPs are currently exempt from Annex 13 compliant labels following advice from the MHRA.

## Source of IMP, Manufacture and Distribution

All trial IMPs; Loratadine, Famotidine, Colchicine and Rivaroxaban are licensed by the MHRA and the Sponsor of the trial will procure the IMPs via DOH (Therapeutic Task Force) procurement. All aspects of drug supply and management will be in accordance with regulatory guidelines and standard local policy and practice for prescription medications.

## Storage and Handling of IMP(s) at Site

Sufficient supply of study IMPs will be stored at the site pharmacy or within the clinics at the Investigator site until they are dispensed for participant’s use. Details of reordering and resupply of IMPs are detailed in the Summary of Drug Arrangements.

. They will be stored as per SmPC. The clinic site/pharmacy will be responsible for the storage and dispensing of the study drug, recording of batch numbers, expiry log and temperature log following local standard procedures and instructions provided in the IMP SmPC.

Destruction will be performed at study sites by study staff, after verification of returned tablets. For patients attending virtual clinics or if the patient does not return to the clinic in person for the 12-week assessment, the patient is advised to return the remaining tablets to the nearest pharmacy for destruction and accountability is performed over the phone.

There are no special requirements for disposal.

The Trial IMPs supplied to Investigator sites are for trial participants only and are to be used only within the context of the trial.

## Accountability of IMP(s)

Participants randomised to drug arm will have their allocation entered into their clinical record and their GP will be informed of allocation. Details of the date of prescription, batch number (all prescriptions will consist of 12-week supply of each IMP), and who issued by, will be recorded in the prescription/clinic log spreadsheet held in the Pharmacy file of the ISF.

At the follow-up time point the participants will be asked to bring back any unused medication for counting. If medications are unused, the reasons for non-usage will be documented and the accountability will be assessed. For follow-up over the phone or online, participants will be asked to count any remaining medications and asked for clarification in relation to the return of any remaining medications to their local pharmacy for destruction. Compliance will be noted on the study CRFs and prescription/clinic log spreadsheet.

## Regulatory Status of the IMP(s)

All drugs used in this trial are licenced for their therapeutic indications. Famotidine and Loratadine are available over the counter. None are currently licensed for the treatment of Long COVID by the UK regulator (MHRA).

## Dosage Schedules

- Loratadine: 10mg tablet orally once a day swallowed whole with water every morning ***PLUS***
- Famotidine: 40mg tablet orally once a day swallowed with water every morning.

Or

- Colchicine: 500mcg tablet orally twice a day swallowed with water every morning.

Or

- Rivaroxaban: 10 mg tablet orally once a day swallowed with water every morning.

IMPs are taken for 12 weeks/84 days.

## Drug Interactions and Specific Contraindications

The appropriate SmPCs provide the full list of the drug interactions and contraindications for all of the trial IMPs and their degrees of severity. A summary of the list of Contraindications and a detailed list of all potential drug-drug interactions are listed in Table 3 below. None of the trial drugs have any documented interaction with any oral or injectable contraceptive agent. Of note, co-prescription of drugs in the classes of serotonin re-uptake inhibitors (SSRIs) and serotonin-norepinephrine re-uptake inhibitors (SNRIs) is permitted in the trial. The risk of increased bleeding, as stated in the SmPC, does not apply to the prophylactic dose of Rivaroxaban 10mg used. Co-prescription of SSRIs/SNRIs with Rivaroxaban 10mg does not affect platelet function and thus does not increase the bleeding risk.

**Table 3** shows the summary of drug-specific interactions for drugs used in the trial and the associated risk management. For the latest information about drug interactions, British National Formulary (BNF) and SmPCs should be referred to for full details of all contraindications to each drug. The current drug contra-indications in the BNF (accessed September 2021) for each of the drugs in this trial are shown below.

**Table 3:** **List of Specific Drug Interactions and contraindications/exclusions with the trial IMPs**

| **IMPS** | **Specific Interactions / exclusions** | **Risk Level / Class** | **Risk Management** |
| --- | --- | --- | --- |
| **Famotidine** | Probenecid | Probenecid inhibits renal tubular secretion of famotidine and can cause a 50% increase in plasma levels of famotidine. | Patients to be excluded from drug trial if taking probenecid |
|  | Sucralfate | Sucralfate inhibits absorption of famotidine. | Patients to be excluded from drug trial if taking Sucralfate |
| **Loratadine** | CNS depressants (such as diphenhydramine, dextromethorphan, pseudoephedrine) | May cause dose-related sedation when administered together | Patients on these drugs will be excluded from the drug trial |
|  | Isocarboxazid  Phenelzine  Tranylcypromine | These drugs may increase the risk of antimuscarinic adverse effects with Loratadine. | Patients taking these drugs at time of screening will be excluded from the drug trial. |
| **Colchicine** | CYP3A4 and systemic treatment with azole-antimycotics or HIV protease inhibitors(Aprepitant, Atanazavir, Clarithromycin, Cobicistat, Crizotinib, Darunavir, Diltiazem, Dronedarone, Erythromycin, Fluconazole, Fosamprenavir, Fosamprenavir, Idelalisib, Imatinib, Isavuconazole, Itraconazole, Ketoconazole, Letermovir, Lopinavir, Netupitant, Nilotinib, Posaconazole, ritonavir, Tipranavir, Verapamil, Voriconazole | May cause induction of increased Colchicine levels beyond therapeutic window, increasing the risk of toxicity and agranulocytosis | Patients on these drugs will be excluded from the drug trial |
|  | Amiodarone, Azithromycin, Ciclosporin, lapatinib, Ranolazine, Vemurafenib | P-glycoprotein inhibitors, increase exposure to Colchicine | Patients on these drugs will be excluded from the drug trial |
|  | Atorvastatin, Bezafibrate, Ciprofibrate, Fenofibrate, Fluvastatin, Gemfibrozil, Pravastatin, Rosuvastatin, Simvastatin, Eliglustat | Colchicine increases the risk of rhabdomyolysis | Patients on these drugs will be excluded from the drug trial |
|  | Creatinine clearance <15ml/minute | Increase exposure to Colchicine | Patients with these conditions will be excluded from the drug trial |
| Rivaroxaban | Active bleeding; antiphospholipid syndrome (increased risk of recurrent thrombotic events); malignant neoplasms at high risk of bleeding; oesophageal varices; recent brain surgery; recent gastro-intestinal ulcer; recent intracranial haemorrhage; recent ophthalmic surgery; recent spine surgery; vascular aneurysm, uncontrolled severe hypertension. Existing anti-platelet therapy following stroke/myocardial infarction | significant risk of major bleeding; | Patients on these drugs will be excluded from the drug trial |
|  | Acalabrutinib, Aceclofenac, Acenocoumarol, Alprostadil, Alteplase, Argatroban, Aspirin, Axitinib, Beniparin, Benzydamine, Bevacizumab, Bismuth, Bivalirudin, Bosutinib, Bromfenac, Cabozantinib, Cangrelor, Caplacizumab, Celecoxib, Cilostazol, Clopidogrel, Cobimetinib, Dabigatran, Dalteparin, Danaparoid, Dasatinib, Dexkeptorofen, Diclofenac, Dipyridamole, Enoxaparin, Epoprostenol, Eptifibatide, Etodolac, Etoricoxib, Flurbiprofen, Heparin, Ibrutanib, Ibuprofen, Iloprost, Imatinib, Indometacin, Inotersen, ketoprofen, Ketorolac, Levatinib, Mefenamic Acid, Meloxicam, Nabumetone, Naproxen, Nicotinic Acid, Nintenanib, Parecoxib, Pazopanib, Phenazone, Phenindione, Piroxicam, Ponatinib, Prasugrel, Regorafenib, Ruxolitinib, Sorafenib, Streptokinase, Sulindac, Sunitinib, Tenecteplase, Tenoxicam, Tiaprofenic Acid, Tecagrelor, Tinzaparin, Tirofiban, Tolfenamic Acid, Trametinib, Traztuzumab Emtansine, Trprostinil, Urokinase, Volanesorsen, Warfarin | concurrent use with these drugs increases the risk of bleeding | Patients on these drugs will be excluded from the drug trial |
|  | Amiodarone, Anagrelide, Cobicistat, Darunavir, Dronedarone, Erythromycin, Fosamprenavir, Itraconazole, Ivacaftor, Ketoconazole, Lopinavir, Neratinib, Nevirapine, Olaparib, Osimertinib, Posaconazole, Ritonavir, Tipranavir, Vandetanib, Vemurafenib, Venetoclax, Voriconazole |  |  |
|  | Atazanavir | CyP3A4 inhibitors increase exposure to Rivaroxaban | Patients on these drugs will be excluded from the drug trial |
|  | Severe hepatic impairment | Increase exposure to Rivaroxaban | Patients with these conditions will be excluded from the drug trial |
|  | Creatinine clearance <15ml/minute | Increase exposure to Rivaroxaban | Patients with these conditions will be excluded from the drug trial |

## Consideration of Dose Modification

Given the design and short intervention period within this study, dose modification will not be used except in cases where a short period of contraindicated antibiotic use is required. For these cases a short cessation of the study drug will be accepted and recorded. Once antibiotic treatment has ceased participant may restart study drug 48 hours after cessation of antibiotic treatment.

## Concomitant Medications

The participants medical records will be accessed by the trial team at the Long COVID Clinic to confirm eligibility (once the participant has consented), all concomitant medications will be recorded and assessed for contraindications to study drugs. The patient will also be asked to confirm if the list is complete, are they taking other prescribed medications from either primary or secondary care clinicians. They will also be asked for details of any over the counter medications, herbal supplements, vitamins, minerals homeopathic or” leisure drugs” e.g. cannabis.

# TRIAL PROCEDURES

## Selection of Participants

Participants will be 18 years or older, with symptoms of Long COVID for a period longer than 4 weeks, based on clinician’s diagnosis, referred to participating Long COVID clinics. The site-specific trial teams are embedded within the Long COVID clinics and will identify potential participants from the clinic list of new referrals. All new referrals will be sent the trial information sheet and contact details for the research team prior to attendance at their first appointment included in the appointment notification letter. This will give participants time to consider taking part prior to their appointment. In instances where the potential participant has not received information then information will be provided at time of approach or clinic visit. The patient will be given as much time as they feel they need to make a decision regarding participation and may consent on that day if they wish or return at a later time point within the 7-10 days post clinic appointment as per patient choice

## Participant Eligibility Criteria

**Participant Inclusion Criteria for ALL Participants**

1. Participants capable of giving informed consent.

2. Age 18 years and above

3. Clinical Parameters; persistent signs and symptoms for a period of 4 weeks or longer in duration post-COVID-19 infection (either by test result or symptomology). Presenting at their first referral first visit to a participating Long COVID clinic pathway.

4. Able to read or understand English or have a relative/family member able to read/understand English to facilitate participation (essential for patient reported outcome measures at follow-up time points and virtual contact).

5. Not enrolled in any other interventional study where study intervention/activities may affect outcome measures (patients enrolled in purely observational studies can be included)

**Additional Participant Inclusion Criteria for the nested, platform randomised drug trial**

(to be eligible participants must meet **all above criteria** and all those below)

*Note: Potential participants with drug-specific contraindications for any arm, including interactions of pre-prescribed essential medication will be consented for data collection but will be excluded from the drug study.*

1. Females of childbearing potential (see definition below) must be willing to use at least an acceptable effective method of contraception during the treatment with IMP and for a further 30 days after the last dose. (34).

Such methods include (:

a. combined (oestrogen and progestogen containing) hormonal contraception:

- 1. oral
  2. intravaginal
  3. transdermal

b. progestogen-only hormonal contraception

1. oral
2. injectable
3. implantable

c. intrauterine device (IUD)

d. intrauterine hormone-releasing system (IUS)

e. bilateral tubal occlusion

f. vasectomised partner

g. male or female condom with spermicide

h. cap, diaphragm or sponge with spermicide i. sexual abstinence; only true abstinence is acceptable i.e. when this is in line with the preferred and usual lifestyle of the participant). (Periodic abstinence, declaration of abstinence during exposure to IMP and withdrawal are not accepted methods of contraception).

Definition of females of childbearing potential:

For the purpose of this trial, a female is considered of childbearing potential i.e. fertile following menarche and until becoming post-menopausal unless permanently sterile. Permanent sterilisation methods include hysterectomy, bilateral salpingectomy, and bilateral oophorectomy.

A post-menopausal state is defined as no menses for 12 months without alternative medical cause.

2. Male Participants must be willing to use condom during IMP treatment to protect their female partner becoming pregnant and for a further 90 days after the last dose.

3. Patients on pre-existing treatments for the same drug classes MUST undergo a 7-day washout period

before being randomised.

*(Patients will be assessed, and if safe to do so, exclude that medication for 7 days, asked if they would be willing to undergo a washout period of at least 7 days before being randomised.)*

**Participant Exclusion Criteria for ALL Participants**

1. Previously hospitalised for COVID-19 infection.

2. Previously referred to a long COVID clinic.

**Exclusion criteria for nested, adaptive randomised drug trial**

3. Females who are pregnant, planning pregnancy or breastfeeding

4. Known hypersensitivity to any of the study drugs or their excipients

5. Currently taking any of the following drugs:

Probenecid, Sucrafate, Isocarboxazid, Phenylzine, Tranylcypromine of any other CNS depressant (such as diphenhydramine, dextromethorphan, or pseudoephedrine) *(Contraindications to famotidine/loratadine)*

Amiodarone, Aprepitant, Atanazavir, Atorvostatin, Azithromycin, Bezafibrate, Ciclosporin, Ciprofibrate, Clarithromycin, Cobicistat, Croztibib, Darunavir, Diltiazem, Dronedarone, Eliglustat, Erythromycin, Fenobibrate, Fluconazole, Fluvastatin, Fosamprenavir, Gemfibrozil, Idelalisib, Imatibib, Isavuconazole, Itraconazole, Ketoconazole, Letermovir, Lopinavir, Netupitant, Nilotinib, Posaconazole, Pravastatin, Ranolazine, Ritonavir, Rosuvastatin, Simvastatin, Tipranavir, Velpatasvir, Vemurafenib, Venetoclax, Verapamil, Voriconazole *(Contraindications to colchicine)*

Acalabrutinib, Aceclofenac, Acenocoumarol, Alprostadil, Alteplase, Argatroban, Aspirin, Axitinib, Beniparin, Benzydamine, Bevacizumab, Bismuth, Bivalirudin, Bosutinib, Bromfenac, Cabozantinib, Cangrelor, Caplacizumab, Celecoxib, Cilostazol, Clopidogrel, Cobimetinib, Dabigatran, Dalteparin, Danaparoid, Dasatinib, Dexkeptorofen, Diclofenac, Dipyridamole, Enoxaparin, Epoprostenol, Eptifibatide, Etodolac, Etoricoxib, Flurbiprofen, Heparin, Ibrutanib, Ibuprofen, Iloprost, Imatinib, Indomethacin, Inotersen, Ketoprofen, Ketorolac, Levatinib, Mefenamic acid, Meloxicam, Nabumetone, Naproxen, Nicotinic acid, Nintenanib, Parecoxib, Pazopanib, Phenazone, Phenindione, Piroxicam, Ponatinib, Prasugrel, Regorafenib, Ruxolitinib, Sorafenib, Streptokinase, Sulindac, Sunitinib, Tenecteplase, Tenoxicam, Tiaprofenic acid, Ticagrelor, Tinzaparin, Tirofiban, Tolfenamic acid, Trametinib, Traztuzumab emtansine, Trprostinil, Urokinase, Volanesorsen, Warfarin *(Contraindications to Rivaroxaban)*

6. Renal failure/insufficiency (eGFR<15ml/minute) on the basis of blood investigations (eGFR) within the last 6 months and clinical assessment

7. Severe liver dysfunction on the basis of blood investigations within the last 6 months (liver function and coagulation) and clinical assessment

## Participant Identification

The participants are identified by referral to a participating Long COVID clinic. All patients scheduled for their first clinic appointment are eligible for participation within the study.

All newly referred patients will be sent a clinic appointment letter which will also contain a study invitation letter and a study information leaflet. The invitation will ask patients to consider taking part and provide contact details of the site study team. The patient can contact the team prior to their clinic appointment and ask any questions they may have and, if willing, make an appointment for eligibility assessment and consent prior to clinic, at the time of clinic or in the 7 -10 days after their clinic appointment. Only consent for data collection and blood collection can be taken prior to clinic. Randomisation into the drug study can only occur after confirmation of eligibility by the clinic team and the trial team

Potential participants will also be given information about the study by their treating physician at the Long COVID clinic appointment.

Eligibility for the drug arm must be confirmed by the PI or suitably delegated person as per delegation log, following results of clinical assessments, to confirm the participant meets all the inclusion criteria and none of the exclusion criteria.

## Recruitment of Trial Participants

It is estimated that there are potentially 30,000 adults with Long COVID across our initial 6 areas. However, referrals may be limited by clinic capacity and GP referral trends. We estimated that it is feasible to consent 4520 patients into the drug study through the referral of these patients to the Long COVID Clinics. Total participant recruitment is expected to be more than this to accommodate those that are ineligible for the drug arm and those that choose not to participate in the drug arm to consent to data collection and blood sample collection.

PCNs within the areas served by the individual clinic will be cluster randomised to receive as usual care:

1. Coverscan™ + Living with COVID Recovery™ App.
2. Coverscan™ + Usual Care Self-Management Rehabilitation.
3. No Coverscan™ + Living with COVID Recovery™ App.
4. No Coverscan™+ Usual Care Self- Management Rehabilitation.

Site-specific research practitioners embedded in the Long COVID Clinics will identify new referrals and, if from within a PCN with Coverscan™ as usual care will arrange a scan appointment 4 weeks prior to the first Long COVID clinic appointment. This appointment is usual care within this PCN and will follow the same pathway as other clinical appointments.

Similarly, patients referred from PCNs allocated to “Living with COVID recovery™” app will be identified and referred to the physiotherapist/s assigned to delivery of this app, who will contact participants directly (as usual care). An appointment with the participant may be required prior to the Long COVID clinic to discuss the App. wherever possible or within 7 -10 days of the first Long COVID clinic appointment.

Some patients interested in participating in the trial may have “brain fog” and, therefore, may require more time to consider taking part. We anticipate a gap of around 4-6 weeks between referral by the GP and patient’s attendance to the appointment at the Long COVID clinic. Wherever possible, patients will be sent details of the study (the options to participate in all or part of the study) within the first appointment letter. This will include the study details, directions to the study website and contact details of both the research team based at their clinic and Lancashire CTU. If they wish to know more about the study prior to their first clinic appointment, then they can contact the team. We will also include details of any participation timeline i.e. eligibility cannot be determined for the drug study prior to the first appointment, consent for all arms should be no later than 7-10 days after the first appointment, and not all patients will be eligible. In instances where the potential participant has not received information then information will be provided at time of approach or clinic visit. The patient will be given as much time as they feel they need to make a decision regarding participation and may consent on that day if they wish or return at a later time point within the 7-10 days post clinic appointment as per patient choice

We will explain the value of data collection from all participants for the cluster trial and contribution to the blood sample collection in particular, to engage with those participants who may not be eligible for the drug trial but can make a valuable contribution to the study and its outcomes.

For patients where English may not be their first language, the information will contain details of how they can take part assisted by an English-speaking relative/friend.

### Informed Consent

The Principal Investigator (PI) retains overall responsibility for the conduct of research at their site. They must ensure that any person delegated responsibility for any study activities is duly authorised, trained, and competent in the principles of Good Clinical Practice (GCP) and the Declaration of Helsinki. If delegation of consent is acceptable, then details should be provided.

The participant remains free to withdraw at any time from the trial without giving reasons and without prejudicing his/her further treatment and must be provided with a contact point where he/she may obtain further information about the trial. Data and samples collected up to the point of withdrawal will be used after withdrawal if the participant has consented for this. Any intention to utilise such data shall be included and outlined in the consent form. It will be made clear that, if data has been anonymised and aggregated, it will not be possible to identify individual data for withdrawal. It will also not be possible to identify any one person from such data.

Participants will be notified that their identifiable data (Name, date of birth, NHS Number, and contact details) contact be shared with LCTU to enable contact for follow-up within the study, and for well-being reporting if needed.

This trial will only recruit participants who are able to consent. If a participant later becomes incapacitated, the participant shall be withdrawn from the trial and no further data will be collected or any trial investigation or assessments conducted. It is not expected that this would be disease progression within this group. Assessments relating to safety issues arising from participation will continue until the trial ends but will be the remit of the patient`s clinician.

Participants allocated to the cluster with the Living with COVID Recovery™ digital app will be informed that data related to their symptoms, physical and mental health, heart rate and physical activities, through the use of the app will be used to inform their care plan by the Multidisciplinary Team at the Long COVID clinics. The App forms part of their usual care and is part of the clinical record: data from consented patients will be used by the trial team to assess the use of the app (level of engagement) and use of content-specific areas within the app.

### Informed Consent Procedure

Patients should be given sufficient time after receiving the trial Participant Information Sheet and Informed Consent Form to consider and discuss participation in the trial with friends and family (adequate time). The person taking consent will be GCP trained, suitably qualified and experienced, and will have been delegated this duty by the CI/ PI on the study delegation log.

We anticipate that patients will receive study information in their Long COVID clinic appointment letter so will have had ample time to read this, go to the study website or contact the research team for information. Patients may consent at the time of clinic or if they wish to have more time to consider, in the 7 to 10 days following this first appointment. In instances where the potential participant has not received information prior to clinic then information will be provided at time of clinic visit. The patient will be given as much time as they feel they need to make a decision regarding participation and may consent on that day if they wish or return at a later time point within the 7-10 days post clinic appointment as per patient choice.

All the data collected has to cover the same treatment pathway and trial assessments timeline as other participants so patients may not enter the drug study after this timepoint to ensure that their 12-week assessment is current to their clinic pathway and the administration of IMP (if allocated to that arm). This is also relevant to the “before” and “after” data assessments in the data collection.

At the time of the Long COVID clinic appointment, a member of the research team will be present at clinic (if face to face) for queries and information giving prior to the participant signing the consent form. For virtual clinics patients will be initially screened over the telephone and then asked to attend in person for full eligibility checking if wishing to participate in the drug study or give research and biobank bloods. Participants on contraindicated medications requiring 7 day wash out period on screening would be consented and randomised after this period following full eligibility checking. It is not possible to randomise patients to the nested drug trial until after the first clinic assessment and confirmation of eligibility. However, patients with known contraindications to the drug study or those that do not wish to participate in the drug study may be consented to data collection or data collection + blood samples at that visit.

Trial sites will use a proactive approach (in keeping with COVID restrictions, vulnerability/fatigue, and equity of access for this group) to contact those patients who have not already expressed an opinion or been asked to participate, to ascertain interest in taking part.

Patients who are too fatigued to attend in person or who are attending a virtual clinic will be given the opportunity to take part in data collection by verbal consent. For patients who express this preference, a telephone consent form (which only the research team at the participating site study will complete) and a copy will be sent by post or email to the patient for their reference. This will also include another copy or e-copy of the participant information sheet for those who have not received one, or who have asked for another copy for their records.

After having time to consider, a member of the site study team will contact them to ask if they would like to participate. The staff member will read out each consent statement and ask the patient if they agree to each one. The staff member will initial and date all the statements. This consent form will be counter signed by the PI. This will be considered full consent given by the participant. They will not be asked to complete and/or return a separate consent form. Copies of this consent will be treated as other consents with a copy sent to the patient (either postal or email as per patient choice), a copy in the site file and a copy in the medical records.

Consent for blood samples and the drug study will not be available in these formats and will continue to be face-to-face consent only.

For those able to attend and sign in person, a copy of the signed, informed consent form will be given to the participant. The original signed form will be retained in the trial file at site, and a copy placed in the medical notes.

The study will use utilise a suite of consent forms to reflect the different options available to participants. The eligibility check form will enable staff to identify which consent form is suitable for each participant.

Consent form options:

1. Data collection (face to face)

2. Data collection and research blood sample donation (face to face)

3. Data collection and drug arm (face to face)

4. Data collection, drug arm and research blood sample donation (face to face)

5. Data collection (telephone)

The PIS and consent form will be reviewed and updated as necessary throughout the trial (e.g. where new safety information becomes available) and participants will be re-consented as appropriate.

As part of the consent procedure, all participants in all sites who have consented for bloods will be asked to confirm if they would be willing to be contacted at the 12 or 24 week time point for further blood sampling if early analysis suggests emerging evidence of utility.

Consent for further future contact is also asked for, to enable sharing of information to participate in other STIMULATE-ICP activities generated by early findings and other studies in relation to COVID-19 and /or long COVID. Contact will be for up to one year after study closure unless at time of contact participant declined to be contacted again.

# REGISTRATION / RANDOMISATION PROCEDURES

## Patient Registration

All patients consenting to the trial will be registered on the trial enrolment log and given a trial ID number which is a 6-digit number. The first two digits will refer to the Long COVID centre and the last four digits will be unique to the participant. Each centre will be pre-allocated with Participant ID numbers on the trial database. Records of each enrolment log will be kept in the Investigator Site File and a copy will be kept in the Trial Master File (TMF).

Enrolment date is the date of randomisation for participants in the drug arms of the trial, or the date of consent for participants in data collection only and data collection + bloods

## Cluster Randomisation for Usual Care service uplift

Up to 226 Primary Care Network (PCN) sites will be allocated using blocked stratified randomisation using block size of 4 into one of the four 2x2 factorial allocations labelled as a, b, c, d (see below) in ratio 1:1:1:1:

1. Coverscan™ + Living with COVID Recovery™ App.
2. Coverscan™ + Usual Care Self-Management Rehabilitation.
3. No Coverscan™ + Living with COVID Recovery™ App.
4. No Coverscan™+ Usual Care Self- Management Rehabilitation.

Randomisation lists will be created by the study statisticians based at UCL and the Lancashire CTU.

To form the strata, PCNs linked to each clinic site will be ordered first by descending size of patient population and then defined by a single cut off value as large PCNs or small PCNs. Then large PCNs will be sorted by descending deprivation and small PCNs by ascending deprivation and defined by a single cut off value as more or less deprived. Within the four strata of PCNs: large, less deprived; large, more deprived; small, more deprived; and small, less deprived, treatment pathways will be allocated using randomised blocks of size 4. This will reduce bias and ensure that there is balance across the pathways by deprivation and population size within each clinic site.

## Drug Trial Randomisation

Participant randomisation will be undertaken using an online system provided by the company Sealed Envelope™.

Coordinated registration and allocation of participant trial numbers will be required to enrol participants. Blocked randomisation will be used to allocate study participants to one of the treatment groups, stratified by clinic, gender and treatment pathway. The randomisation will be implemented through the online randomisation portal, supplied by Sealed Envelope™ Ltd.

Participants will be assigned to treatment groups through consecutive allocation of subject numbers, and the use of a Trial Subject Enrolment Log which will be stored on the Sealed Envelope™ system.

## Blinding

There will be no blinding for the drug arm of this study: this is an open label study. As this a pragmatic trial with currently unconfirmed drugs being introduced later, for practical reasons it has not been feasible to pursue encapsulation or placebos for multiple drugs. The majority of the key outcomes are patient-reported outcomes and so there is little potential for outcome assessors to introduce bias by knowing cluster or drug allocation. The number of interim analyses in the trials makes blinding of the statisticians impractical.

# TRIAL ASSESSMENTS

## Eligibility Assessments

Patients who have received the study information prior to clinic attendance may contact the study team to confirm eligibility prior to their appointment. If wishing to take part for data collection only, they will have the option to consent in person at clinic or to consent over the telephone (see 7.4.2). All patients will be screened using the eligibility criteria.

For patients who indicate that they want to take part for data collection plus blood sampling/biobank, consent will be face-to-face.

For Patients who indicate that they want to take part in the drug arm or drug arm plus blood sampling and biobank, they will be screened for any contraindication for the drug arm to establish eligibility. For the drug arm, all eligibility and exclusion criteria will be assessed using; blood tests done at the time of referral, GP referral letter content, presenting symptomology, patient discussion and Long COVID clinic notes. If any concerns are noted at clinic then further blood tests may be ordered, the research team will assess these results to confirm eligibility. Eligibility must be confirmed no later than 10 days post clinic appointment. If found to be ineligible, then patients will be excluded from the drug arm and invited to continue for data collection and bloods only.

The following procedures/ test results must be available to confirm eligibility for the nested drug trial:

- Participant full medical history (at Long COVID clinic visit)
- Concomitant medication review (at Long COVID clinic visit)
- Physical Examination (at Long COVID clinic visit)
- Urine pregnancy test within 7 days prior to the first dose of IMP (in women of childbearing potential)
- eGFR (within 6 months)
- Liver function test (within 6 months)
- FBC (within 6 months)
- Coagulation screening (within 6 months)

All Participants, once consented, regardless of participatory arm, will be invited to complete assessments at baseline, 12 and 24 weeks.

## Baseline Visit

At the baseline visit, the patient will be given a copy of the CRF questionnaire to complete at the clinic or to take home and to be contacted by a member of the trial team the following day to complete over the phone. During the visit the following will be recorded:

1. Patient’s Demographics
2. Patient’s full medical history from January 2020 (when COVID was recognised as circulating in the UK) including comorbidities and all COVID-related illnesses, treatments, and vaccinations.
3. Patient’s history of concomitant medication including current, pre and post COVID-19 and over the counter medications
4. Physical Examination, including weight, height, oral temperature, resting pulse, and blood pressure

**Laboratory Tests:**

Routine clinical blood tests performed by the General Practice doctors, and as a result of clinic assessment at the point of referral, will be collected as part of trial data. Blood tests will differ in different clinics based on differences in usual care and capacity and capability to provide within that service. Patients who consent for research blood sampling and for further research will have blood taken and blood tests outlined below will be undertaken at a central laboratory using a batched analysis approach at a later time point. It is not expected that the results of these bloods will directly impact on individual patient usual care during the 12-week clinic pathway.

Approximately 60mls of blood (will be taken at baseline visit in sites that have capacity and capability for further research (translational sub-studies) and biobanking (see section 9.5 for details): In the London site approximately 500 participants will be asked if they would be willing to give further blood (30mls) at 12 weeks for comparative analysis.

As part of the consent procedure all participants in all sites who have consented for bloods will be asked to confirm if they would be willing to be contacted at the 12 or 24 week time point for further blood sampling if early analysis suggests emerging evidence of utility.

**Functional Tests:**

Standard investigations differ from clinic to clinic and the study will not require a standardised testing strategy as that would impact on its aim of assessing usual care; but will collect any, and all data related to individual consented participants tests and investigations (either ordered by the GP at time of referral or ordered as a result of Long COVID clinic appointment). This will include all data related to Coverscan™ , which will be provided as an uplift to usual care in randomised areas.

Examples of functional tests in some clinics are:

1. Chest X Ray
2. High Resolution Computerised Tomography (CT) scan of the Chest
3. CT Pulmonary Angiogram
4. Pulmonary Function Test
5. 6-minute walk test
6. 1-minute sit to stand test
7. Functional Exhaled Nitric Oxide (FeNO) test
8. Echocardiogram (ECHO)
9. Electrocardiogram (ECG) if the patient had cardiac symptoms
10. Holter monitor of the heart
11. Cardiovascular Magnetic Resonance Scan (CMR)
12. Stress Electrocardiogram
13. Magnetic Resonance Imaging (MRI) scan of Brain
14. Tilt Table Test
15. Coverscan™

**Baseline process**

The following process applies to all participants consenting for inclusion in the trial. The Research team will action the following:

- 1. Consent form: Ensure 4 copies of the consent form have been obtained; one copy retained in the patient’s medical record, one copy given to the patient, one copy is stored in the Investigator Site File (ISF) and one copy sent to Lancashire CTU.
  2. Study number allocation: All participants will be allocated a study number via sealed envelope. The date of entry into sealed envelope and the generation of a study number will be day 1 for participants.
  3. Randomisation: The system will then ask the researcher if the participant is willing to enter into the drug study. Once confirmed, the system will then randomise the participant to either drug or usual care. An automated email will be sent to the trial site, with Lancashire CTU confirming participant ID number and allocation arm. If the Participant has consented only for the data collection, there will be a “not randomised” allocation and a Participant ID number.
  4. Tracker database: The research team using encrypted individual password remote access should then enter the participant onto the participant tracker database (held centrally at the Lancashire CTU) and enter participants personal contact details, address, email, in order to contact the participant for follow-up data collection. This participant tracker will not contain any clinical or research data but acts as a contact list for follow-up purposes and GP contact safety reporting. Individual sites will only be able to see data relating to their own participants.
  5. IMP: If the patient is participating in the drug trial, a prescription or the IMP will be issued by the PI or a designated member of the research team and the first dose taken by the participant at the clinic (if feasible depending on the location of the recruiting site at the participating centres).
  6. Wallet card: The participant will be given a Participant Wallet card with the trial registration number with the title and name of the trial drug (if applicable), the name of the PI, emergency contact number of the Research Nurse or the participating site team in case of emergency or hospital admissions.
  7. Research bloods and biobank samples 60 mls (as per Laboratory tests)

**Functional and Patient Reported Outcome Assessments**

All participants must have given consent before the following Patient Reported Outcome Questionnaires and functional tests are carried out:

1. Fatigue Assessment Score(FAS)
2. EuroQol Research Foundation Health related quality of life (EQ-5D-5L)
3. Mental health (GAD-7)
4. Medical Research Council Dyspnoea Score
5. Public Health Questionnaire - Depression (PHQ-9)
6. Perceived Deficit Questionnaire (PDQ-5)
7. Work and Social Adjustment Scale (WSAS) [ Question 4 from Productivity Cost Questionnaire (iPCQ) for absenteeism and Question 8 from iPCQ for presenteeism added]
8. Short Form Questionnaire (SF-12)
9. Cognitive Failure Questionnaire (CFQ) if a patient scores 3 or more on PDQ5 (patients receive an email to complete this questionnaire online via a secure password and patient ID number)
10. Functional abilities and physical function using pedometer monitoring/wearables data
11. Organ impairment and healthcare utilisation
12. Cost-effectiveness of ICP
13. Process outcomes for different ICP components

## Assessment Visit (12 Weeks Assessment)

The following Patient Reported outcome measures and activities will be collected at the 12-week timepoint (+ 7 days) to record the primary and secondary outcome measures. This may not be possible in all patients due to their level of fatigue. Participants will be supported by the flexibility of data collection processes (below) to contribute as much as they are able.

For patients recruited from the London site 500 participants will be invited to return to gift a 30 mls blood sample following completion of their drug treatment for analysis comparison.

Patient reported outcome measures will be collected either at the Long COVID clinics, by post, entered by participants themselves via secure electronic link or over the phone as per participant choice. Any non-patient reported data will be extracted from the clinical record by the site-specific research staff and input on to the eCRF database:

1. Fatigue Assessment Score
2. IMP accountability (over the phone or in person at the clinic)
3. 6-minute walk test (if performed at baseline visit and where possible undertaken at follow-up)
4. 1-minute Sit to Stand test (if performed at baseline visit and where possible undertaken at follow-up)
5. Medical Research Council (MRC) dyspnoea score
6. Modified Work and Social Adjustment Scale (WSAS) [Q4 from iPCQ for absenteeism and Q8 from iPCQ for presenteeism added]
7. General Anxiety Disorder Questionnaire- 7 (GAD-7)
8. The Primary Care Evaluation of Mental Disorders Patient Health Questionnaire (PHQ-9)
9. EQ-5D-5L (EUROQOL-5 domain- 5 level)
10. Perceived Deficit Questionnaire (PDQ-5)
11. 12-item Short Form Survey (SF12)
12. Cognitive Failure Questionnaire (CFQ) if a patient scores 3 or more on PDQ5 (patients receive an email to complete this questionnaire online via a secure password and patient ID number)
13. Adverse Event review (over the phone or in person in clinic) – Patient completed eCRFs or paper questionnaires will be reported back to site PIs by Lancashire CTU, for review and follow-up of any potential AEs reported by patients
14. Concomitant medication review (over the phone or in person at the clinic)

Patient’s details including mobile phone number will be confirmed and documented in the patient’s record for the purpose of sending a reminder digital text approximately 7 days before the next assessment appointment.

## Follow-up Assessment (24 Weeks) Visit

All patients will be followed up for 24 Weeks (+ 7 days) from the enrolment date.

These assessments will be carried out by staff based at Lancashire CTU. Participants will be given the choice of completing questionnaires online using an individualised secure link, by postal return or via telephone for participants requiring support. In the event of receiving an incomplete outcome assessment, the patient will be contacted by phone within 7 days of the team receiving the form (e-form or paper) and complete the missing information over the phone with the patient. The data will be entered into the trial database by a member of staff at Lancashire Clinical Trials Unit.

The following Patient Reported Outcome measures and functional assessments are recorded at the 24 weeks follow up visit:

1. Fatigue Assessment Score
2. IMP accountability (over the phone or in person at the clinic)
3. 6-minute walk test (if performed at baseline visit and where possible undertaken at follow-up)
4. 1-minute Sit to Stand test (if performed at baseline visit and where possible undertaken at follow-up)
5. MRC dyspnoea score
6. Modified Work and Social Adjustment Scale (WSAS) [Q4 from iPCQ for absenteeism and Q8 from iPCQ for presenteeism added]
7. General Anxiety Disorder Questionnaire- 7 (GAD-7)
8. The Primary Care Evaluation of Mental Disorders Patient Health Questionnaire (PHQ-9)
9. EQ-5D-5L (EUROQOL-5 domain- 5 level)
10. Perceived Deficit Questionnaire (PDQ-5)
11. 12-item Short Form Survey (SF12)
12. Cognitive Failure Questionnaire (CFQ), if a patient scores 3 or more on PDQ5 (patients receive an email to complete this questionnaire online via a secure password and patient ID number)
13. Functional ability and Fidelity of delivery of Treatment as Usual and Living with COVID Recovery ^TM^
14. Adverse Event review (over the phone or in person in clinic) for participants on the nested drug trial and participants expressing suicidal ideation on the patient reported outcome questionnaires – Patient completed eCRFs or paper questionnaires will be reported back to site PIs by Lancashire CTU, for review and follow-up of any potential AEs reported by patients. AEs will be reported up to 28 days following the last dose of the trial drugs.
15. Concomitant medication review (over the phone or in person at the clinic) for participants on the nested drug trial only. Concomitant medications will be reported up to 28 days following the last dose of the trial drugs.

The outcome from these questionnaires constitutes the secondary end points for the trial.

## Laboratory Procedures

It is the responsibility of the trial site to ensure that samples are appropriately labelled in accordance with the trial procedures to comply with the Data Protection Act 2018. Biological samples collected from participants as part of this trial will be transported, stored, accessed and processed in accordance with national legislation relating to the use and storage of human tissue for research purposes and such activities shall at least meet the requirements as set out in the 2004 Human Tissue Act and the 2006 Human Tissue (Scotland) Act.

### Local analysis of Clinical Samples

Samples of blood for clinical routine care and to assess eligibility for the drug are taken by the treating physician at the Long COVID clinics (baseline visit) if clinically indicated.

Bloods that may need to be taken for eligibility purposes if not available with the specified timelines (see section 9.1). These may include:

- LFT
- FBC
- eGFR
- Urine Pregnancy test
- Coagulation screening including (platelet count, activated partial prothrombin time, Prothrombin time and bleeding time)

The data from these analyses will inform the eligibility and the outcome of this trial. Clinics will follow their own local policies and procedures relating to the management of samples at NHS sites.

### Central Analysis and Storage of Clinical Samples for Research

For those participants consenting for research bloods a total of 60 mls (90 mls in total for participants at the London Site ) will be taken. Blood will either be taken at clinic; for those clinics able to support the taking and processing of the samples or at a centralised facility within the area served by the clinic. The data from these analyses will inform the outcome of this trial and future research.

Research blood samples will be labelled with trial ID number, date and time taken. If the sample requires preparation locally by the site staff, the procedure will be outlined in a trial specific laboratory manual for necessary preparation and separation of different components at site, before storage and transfer, via courier ,to the Oxford Community Diagnostic Laboratory (Perspectum), biobank and onwards to corresponding laboratories for further analysis.

In the case of UCLH patient samples for Functional T-cell and live-virus neutralisation antibodies assay, they will be sent direct from the UCLH site to the Francis Crick Institute for analysis**.**

#### **9.5.2.2 Samples for Future Research and Biobanking Storage at Oxford Community Diagnostic Laboratory (Perspectum)**

Approximately 5mL of the research blood taken will be for biobanking at Perspectum and use in future ethically approved research studies research regarding the pathophysiology and mechanism of long COVID, where separate ethical approval may be required. This blood will be stored in an HTA registered biobank at Perspectum’s central laboratory and UCL will remain the custodian.

#### **9.5.2.3 Samples for Sub-Study Analysis at Central Laboratories**

Approximately 60 mls of the blood sample will be used for the analyses set out below and relate to the secondary endpoint of the trial. The frozen components of the blood samples will be stored at Perspectum’s central laboratory and sent on to sub-contracted laboratories for testing. In the case of UCLH patient samples for Functional T-cell and live-virus neutralisation antibodies assay, they will be sent direct from the UCLH site to the Francis Crick Institute for analysis**.**

Guided by clinical practice, patient lived experience and latest scientific hypotheses, the following analyses will be performed in blood samples of some participants, extending to the whole cohort only if there is a clear scientific rationale. Any samples not used within these sub-studies will be retained within the biobank at Perspectum for future research use. All samples will be sent to Perspectum and stored until analysis by third party laboratories:

1. **Genomics analysis:** one sample per patient will be taken for initial genome-wide and focused gene (using a long list of immune-regulated genes) analyses. They will be performed using standard protocols adjusting for any population structure. Models will incorporate clinical and environmental determinants of disease severity.
2. **Proteomics analysis** one sample per patient. Proteomics will be assessed by proximity extension assay enabling over 1400 proteins to be rapidly analysed. The assay uses oligonucleotide-labelled antibody pairs allowing for pair-wise binding to target proteins.
3. **Metabolomics and Lipidomics**; one sample per patient : A combination of Liquid Chromatography with tandem mass spectrometry (LC-MS/MS) based metabolomics and lipidomics will be performed based on a targeted analysis of over 200 metabolites of core metabolism, including acyl-carnitines, acyl-CoAs, amino acids, glycolysis and TCA intermediates and nucleotides using a Thermo Quantiva triple quadrupole mass spectrometer and lipidomics by open-profiling UHPLC-MS/MS using a Thermo Elite Orbitrap interfaced with an Advion Nanomate to allow direct nanoinfusion to detect over 600 annotated lipids.
4. **Functional T-cell and live-virus neutralisation antibodies** in participants recruited to the London trial site. Two samples per patient will be collected (approximately 30mL). Serum and peripheral blood mono-nuclear cells (PBMCs) will be isolated on arrival at the FCI. Cells will be stained and analysed using mass-cytometry and neutralising antibodies quantified in the live-virus neutralisation assay.
5. **Endocrine investigation** (thyroid, hypothalamo-pituitary- gonadal and hypothalamo-pituitary- adrenal axes) will be included for those with suggestive symptoms associated with thyroiditis, autoimmune hypothyroidism and adrenal impairment for detailed phenotyping to define potential pathophysiology involved in ongoing organ-specific or physiological abnormalities for example endocrine disturbances explaining diverse nonspecific symptoms, including fatigue, hypothermia and dysmenorrhoea.

## Schedule of Assessments

**Table 4** **Schedule of Assessments**

|  | STIMULATE-ICP Trial Schedule of Assessments | | | | | | | | | | |
| --- | --- | --- | --- | --- | --- | --- | --- | --- | --- | --- | --- |
|  | Procedures | | Referral to LCC and /or Coverscan^TM*^ | | First Long COVID Clinic Visit | | Baseline/ Visit | | Treatment / Assessments | | Follow Up / Assessments |
| Visiting window |  | |  | | Day 0 | | No later than 7-10 days post clinic* | | 12 weeks (+7days)*** | | 24 weeks (+7days)**** |
| Visits No |  | |  | | **0** | | **1** | | **2** | | **3** |
|  | **Informed consent** | |  | | **X*** | |  | |  | |  |
| Patient's History | **Demographics** | |  | |  | | **X** | |  | |  |
|  | **Medical history** | |  | |  | | **X** | |  | |  |
|  | **Concomitant Medications** | |  | |  | | **X** | |  | |  |
| Physical examination | **Height, Weight, Temperature** | |  | |  | | **X** | |  | |  |
|  | **Resting Pulse and BP** | |  | |  | | **X** | |  | |  |
| Laboratory Tests^1^ | **FBC** | |  | |  | | **X** | |  | |  |
|  | **LFT** | |  | |  | | **X** | |  | |  |
|  | **eGFR** |  | |  | | **X** | |  | |  | |
|  | **Coagulation screening** |  | |  | | **X** | |  | |  | |
|  | **Urine pregnancy test^5^** | |  | |  | | **X** | |  | |  |
|  | **COVID-19 Antibody** | |  | |  | | **X** | |  | |  |
| Sample Collections | **samples of blood for biobank storage at Perspectum, Genomics, proteomics, metabolomic & Lipidemic, functional T cell and live virus Neutralisation antibody, Endocrine analysis** | |  | |  | | **X** | | **X ^6^** | |  |
| Cardiovascular | **(ECG, ECHO, Holter Monitor, Stress Electrocardiogram)^1^** | |  | |  | | **X** | |  | |  |
| Pulmonary | **CXR or CMR, CT Pulmonary angiogram^1^** | |  | |  | | **X** | |  | |  |
|  | **6 Minute Walk Test, 1-minute Sit to Stand test,** | |  | |  | | **X** | | **x** | | **x** |
|  | **PFT (FeNo)^1^** | |  | |  | | **x** | |  | |  |
| CNS | **MRI of brain^1^** | |  | |  | | **X** | |  | |  |
|  | **Tilt Table Test** | |  | |  | | **X** | |  | |  |
| Whole Body | **Coverscan^TM*^  **** | | **X** | |  | |  | |  | |  |
| Inclusion and Exclusion | **Eligibility assessment** | |  | |  | | **X** | |  | |  |
|  | **Randomisation iv** | |  | |  | | **X** | |  | |  |
| IMP | **Dispensing of trial IMP ^3^** | |  | |  | | **X** | |  | |  |
|  | **Accountability** | |  | |  | |  | | **X** | |  |
| Patient Reported Outcome Assessments | **FAS Assessment** | |  | |  | | **X** | | **X** | | **X** |
|  | **MRC Dyspnoea Score** | |  | |  | | **X** | | **X** | | **X** |
|  | **General Anxiety Disorder Questionnaire- 7 (GAD-7)** | |  | |  | | **X** | | **X** | | **X** |
|  | **The Primary Care Evaluation of Mental Disorders Patient Health Questionnaire (PHQ-9)** | |  | |  | | **X** | | **X** | | **X** |
|  | **EQ-5D-5L (EUROQOL-5 domain- 5 level)** | |  | |  | | **X** | | **X** | | **X** |
|  | **Perceived Deficit Questionnaire) PDQ-5** | |  | |  | | **X** | | **X** | | **X** |
|  | **SF12** | |  | |  | | **X** | | **X** | | **X** |
|  | **Cognitive Failure Questionnaire (CFQ) ^4^** | |  | |  | | **X** | | **X** | | **X** |
|  | **Modified Work and Social Adjustment Scale (WSAS)** | |  | |  | | **X** | | **X** | | **X** |
| Safety Assessments | **Adverse Event Reporting** | |  | |  | |  | | **X** | | **X**^b^ |
| Concomitant medication | **Concomitant medication review** | |  | |  | |  | | **X** | | **X^7^** |

- Consent for data collection only may take place before first clinic visit and up to 10 days post clinic. Consent for drug arm must be at clinic visit (to assess eligibility based on clinic record) or in the 7 days post clinic. This is to align the administration on IMP with follow-up data collection and clinic pathway. Consent for research blood to be taken at either time point as per patient choice.

******Coverscan as usual care for allocated clusters

*******: Assessment 1 is 12 weeks from baseline visit date (date of randomisation or first dose of study IMP) or consent date at baseline visit (if only consented to data collection)

********: Follow up assessment 24 weeks from baseline visit date (date of randomisation or first dose of study IMP) or consent date at baseline visit (if only consented to data collection)

1: If requested by the treating physician at Long COVID Clinics as part of their routine care

2: Only if recruited from a site randomised to Coverscan™ cluster

3: If randomised to receive the trial drug

4: if the participant scores >3 on PDQ5 questionnaire

5: For female participants of childbearing potential randomised to drug arm of the trial

6: blood samples collected for functional T cell and live virus neuralisation assays from London Participants only at baseline and12 weeks. All other participating centres will collect blood for external laboratory analysis once at baseline visit. Permission will be sort from all participants to recontact to participate in further testing due to any changes to the study on the basis of emerging evidence/results

7. concomitant medications will be reviewed at 12 and 24 weeks visit is only for participants o the nested drug trial

b. Adverse events will be collected at the 24 week visit for participants on the nested drug trial only.

Randomisation to the drug arm can only be after clinic attendance. Eligibility will be ascertained; the participant will be consented face to face and randomised at the same visit.

## Post-Trial Treatment

There are no arrangements for the supply of the study drugs following the end of the 12-week treatment and assessment for research purposes. However, if in the opinion of the treating physician the treatment will benefit patients, it is in their discretion to continue with the treatment for as long as clinically indicated as per local trust policy. The supply of the drugs for long term use is at the discretion of the treating physician.

# DISCONTINUATION / EARLY CESSATION OF TREATMENT AND “STOPPING RULES”

## Replacements

Withdrawn participants will not be replaced. Participants who only stop treatment early have not stopped participation in the trial for the purpose of intention to treat. However, participants who withdraw consent will have their data analysed to the point of withdrawal. The PIS will make it clear that Participants may remain in the trial if they stop treatment early or are not eligible for the drug trial for any reason, for the purpose of data collection and intention to treat. No data will be removed from the analysis once collected.

## Cessation of Treatment and Withdrawal

Participants may be withdrawn from the trial or their treatment discontinued by the PI or after withdrawal of consent by the participant. It is always within the remit of the physician responsible for a patient to stop participation in a trial (or certain aspects of the trial) for appropriate medical reasons, be they individual adverse events or new information gained about a treatment.

The reasons for withdrawal should be documented on the CRFs as soon as it occurs, and alternative treatment plan should be provided and documented on the patient’s notes. The possible reasons for withdrawal of participants include:

1. On clinical grounds such as abnormal laboratory tests
2. Severe or intractable side effects deemed to be secondary to the trial IMP administration, that cannot be resolved without discontinuation of the study drug
3. Other significant adverse reactions
4. Patient choice to withdraw consent for treatment
5. Patient choice to withdraw consent for follow up
6. Patient’s choice to withdraw from all activities of the trial including data collection and retention.
7. Following safety review by the TSC/IDMC
8. Severe disease where palliative approach is adopted

All side effects should be reported initially to the treating physician and the site trial team responsible for their treatment as adverse events. The physician responsible for the patient may decide the side effects are severe enough to necessitate cessation of the trial drug immediately. For milder side effects, a discussion with the study participant should be held to ascertain if the participant is willing to continue. If the participant is unwilling, then all study medications should cease.

Cessation of drugs within the trial should occur in the following conditions; all reasons for cessation must be recorded in the appropriate CRF page, and reported to Lancashire CTU/CI/ Sponsor as appropriate:

1. Reported adverse effects, if deemed severe enough to necessitate early cessation of trial drug by the physician responsible for the patient.
2. A study participant is diagnosed with a new condition that necessitates cessation of the trial drug or modification of the dose.
3. A study participant is intolerant of the prescribed dose or reports significant adverse effects.
4. Where the trial drug is newly contraindicated by a new diagnosis.
5. Report of a new pregnancy in female participants.

A decision by a participant that they no longer wish to continue receiving the trial drug should **not** be considered to be a withdrawal of consent for follow-up or data collection unless the patient explicitly expresses that they no longer want their data collected or to be followed up. However, participants are free to withdraw consent for some or all aspects of the study at any time if they wish to do so. Ideally, they should understand the implications of this. In accordance with regulatory guidance, de-identified data that have already been collected and incorporated in the study database will continue to be used (and any identifiable data held by LCTU will be destroyed).

Participants who lose capacity during the study will be withdrawn from the trial and no further data will be collected or any trial investigation or assessments conducted.

The trial may be prematurely discontinued by the Sponsor, CI, or regulatory authority based on new safety information or for other reasons given by the Ethics Committee, MHRA or TSC on recommendations of the regulatory authority concerned.

If the trial is prematurely discontinued, active participants will be informed and no further participant data will be collected. The MHRA and Research Ethics Committee will be informed within 15 days of the early termination of the trial.

## Lost to Follow-up

If a participant moves from the area, every effort should be made for the participant to be followed up at another participating trial site and for this new site to take over the responsibility for the participant.

If a participant is lost to follow-up at a site every effort should be made to contact the participant’s GP to obtain information on the participant’s status. The PIL and ICF will include consent to contact the participant’s GP to obtain information about the participant in relation to the treatment or inclusion in the trial.

# SAFETY REPORTING / PHARMACOVIGILANCE

Collection, recording and reporting of adverse events to the sponsor will be completed according to the sponsor’s SOP for the Recording, Management and Reporting of Adverse Events by Investigators (JRO/INV/S05).

## Definitions

**Table 5: Definitions**

| Term | Definition |
| --- | --- |
| Adverse Event (AE) | Any untoward medical occurrence in a subject to whom a medicinal product is administered and which does not necessarily have a causal relationship with this treatment.  *Therefore an AE can be any unfavourable or unintended change in the structure (signs), function (symptoms) or chemistry (laboratory data) in a subject to whom an IMP has been administered, including occurrences which are not necessarily caused by or related to that product.* |
| Adverse Reaction (AR) | A response to a medicinal product which is noxious and unintended, and which occurs at doses normally used in man for the prophylaxis, diagnosis, or therapy of disease or for the restoration, correction, or modification of physiological function.  *This definition implies a reasonable possibility of a causal relationship between the event and the IMP. This means that there are facts (evidence) or arguments to suggest a causal relationship.*  *This definition also covers medication errors and uses outside what is foreseen in the protocol, including misuse and abuse of the product.* |
| Serious Adverse Event (SAE)  or  Serious Adverse Reaction (SAR) | Any adverse event or adverse reaction in a trial subject that:   - Requires inpatient hospitalisation or prolong action of existing hospitalisation   *Note: hospitalisation is defined as an inpatient admission, regardless of length of stay, even if the hospitalisation is a precautionary measure for continued observation. Therefore, participants do not need to be hospitalised overnight to meet the hospitalisation criteria. Hospitalisation (including hospitalisation for an elective procedure) for a pre-existing condition (prior to study entry) which has not worsened does not constitute a serious experience*   - Results in persistent or significant disability or incapacity:   *Note: substantial disruption of one’s ability to conduct normal life functions*   - Results in a congenital anomaly or birth defect   *Note: in offspring of subjects or their partners taking the IMP regardless of time of diagnosis*   - is life threatening; or   *Note: places the subject, in the view of the investigator, at immediate risk of death from the experience as it occurred, this does not include an adverse experience that, had it occurred in a more severe form, might have caused death;*   - Results in death   Some medical events may jeopardise the subject or may require an intervention to prevent one of the above characteristics/consequences. Such important medical events should also be considered as serious.  The term “severe” is often used to describe the intensity of an event or reaction (e.g. mild, moderate, or severe) and should not be confused or interchanged with the term “serious”. |
| Suspected Unexpected Serious Adverse Reaction (SUSAR) | A serious adverse reaction, the nature, severity, or outcome of which is not consistent with the Reference Safety Information (RSI). |
| Reference Safety Information (RSI) | A list of medical events that defines which reactions are expected for the IMP being administered to clinical trial subjects, and so do not require expedited reporting to the MHRA. It is contained in a specific section in the Summary of product characteristics (SmPC) or the Investigator Brochure (IB). |

## Recording and reporting of Adverse Events

All adverse events (AEs) will be recorded in the participant’s medical records in the first instance, regardless of the allocation of the participant in the nested drug trial or the cluster trial.

Adverse events will be recorded with clinical symptoms and accompanied with a simple, brief description of the event, including dates as appropriate.

AEs for participants on the data collection arm do not need to be recorded in the eCRF.

For participants enrolled in the nested drug trial all AEs will be recorded in the eCRF following consent. For participants randomised to standard of care drugs only, AEs will be recorded until 12 weeks post enrolment. For participants randomised to receive an IMP, AEs will be recorded until 28 days following last dose of an IMP .

Where possible, a diagnosis rather than a list of symptoms should be recorded. If a diagnosis has not been made, then each symptom should be listed individually. All AEs should be captured on the appropriate AE pages in the eCRF.

## Assessing Adverse Events

Each adverse event will be assessed for severity, causality and seriousness as described below:

### Severity

The intensity will be determined by using the following definitions:

**Table 6:** **Definitions of grades of severity of the Adverse Events**

| **Category** | **Definition** |
| --- | --- |
| **Mild** | The adverse event does not interfere with the participant’s daily routine and does not require intervention; it causes slight discomfort. |
| **Moderate** | The adverse event interferes with some aspects of the participant’s routine, or requires intervention, but is not damaging to health; it causes moderate discomfort. |
| **Severe** | The adverse event results in alteration, discomfort or disability which is clearly damaging to health. |

### Assessing Causality

The assessment of relationship of adverse events to the administration of IMP must be made by the investigator (or delegated medically qualified person). It is based on clinical judgement using all available information at the time of the completion of the CRF.

The following categories will be used to define the causality of the adverse event:

**Table 7:** **Definitions of relatedness of the SAEs to the trial drugs**

| **Category** | **Definition** |
| --- | --- |
| **Related** | A causal relationship between an IMP/investigational treatment and an adverse event is at least a **reasonable possibility**, i.e. the relationship cannot be ruled out. |
| **Not Related** | There is **no reasonable possibility** of a causal relationship between an IMP/investigational treatment and an adverse event. |

### Seriousness

All events are assessed for seriousness as defined for an SAE in the definitions table.

## Assessing Serious Adverse Events and Serious Adverse Reactions (nested drug trial participants only)

All Serious Adverse Reactions (SAEs) occurring from the time of written informed consent until 28 days after the last dose of the IMP, or the end of their assessments at 12 Weeks for participants on standard of care drugs only, must be recorded in the medical records, the eCRF and the trial specific SAE Reporting Form and reported to the Sponsor **within 24 hours** of the research staff becoming aware of the event.

The Investigator or designated individual will complete the Sponsor’s trial specific SAE Reporting Form and email it to the Sponsor at [**SAE@ucl.ac.uk**](mailto:SAE@ucl.ac.uk) **and** [**LCTUSTIMULATE@uclan.ac.uk**](mailto:LCTUSTIMULATE@uclan.ac.uk)**.**  The Investigator will respond to any SAE queries raised by the Sponsor as soon as possible.

Completed SAE Reporting Forms must be sent to the Sponsor within 24 hours of becoming aware of the event

**Email SAE Forms to:** [**SAE@ucl.ac.uk**](mailto:SAEreporting@uclan.ac.uk) **and LCTUSTIMULATE@uclan.ac.uk**

Any change of condition or other follow-up information should be emailed to the Sponsor, on an SAE Reporting Form (clearly marked as follow-up) as soon as it is available or at least within 24 hours of the information becoming available.

Events will be followed up until the event has resolved or a final outcome has been reached. SAE follow-up should continue after completion of protocol treatment and/or trial follow-up if necessary. Any SAR will need to be reported to the Sponsor, irrespective of how long after IMP administration the reaction has occurred until resolved.

The SAE listing will be reported from each site to the CI and Sponsor upon request.

## Serious Adverse Events Which Do Not Require Reporting to Sponsor

SAEs for participants on the data collection only arm of the trial do not need recording in the eCRF or reporting to Sponsor with the exception of the SAEs which are suggestive of suicidal ideation following review of participant completed questionnaire which will be recorded and reported to the Sponsor.

For participants enrolled in the nested drug trial, the following events do not require immediate reporting to the sponsor as SAEs, however they will still be recorded in the participant’s medical records.

Hospitalisation for:

- Routine treatment or monitoring of the studied indication not associated with any deterioration in condition.
- Any admission to hospital or other institution for general care where there was no deterioration in condition.
- Treatment on an emergency, outpatient basis for an event not fulfilling any of the definitions of serious as given above and not resulting in hospital admission.

## SUSAR Reporting

All SAEs assigned by the PI or delegate as suspected to be related to IMP-treatment (SARs) will be assessed for expectedness against the current approved RSI for the trial by the Sponsor.

The following categories will be used to define the expectedness of the SAR:

**Table 8:** **Definitions of expectedness of the SARs**

| Category | Definition |
| --- | --- |
| *Expected* | An adverse event which is consistent with the information about the IMP listed in the current approved Reference Safety Information (RSI) for the trial. |
| *Unexpected* | An adverse event which is not consistent with the information about the IMP listed in the current approved Reference Safety Information (RSI) for the trial. |

All SARs assessed as unexpected will be classified as SUSARs and will be subject to expedited reporting to the MHRA and REC.

The RSI to be used to assess expectedness against the IMPs are:

- SmPC Famotidine 40mg Tablets (Tillomed Laboratories Ltd), Section 4.8: Undesirable effects
- SmPC Clarityn Allergy (loratadine) 10mg Tablets (Bayer plc), Section 4.8: Undesirable effects
- SmPC Colchicine Tablets BP 500 mcg (Wockhardt UK Ltd), Section 4.8: Undesirable effects
- SmPC Xarelto (Rivaroxaban) 10 mg film-coated tablets (Bayer plc), Section 4.8: Undesirable effects

The sponsor will inform the MHRA and REC within the required expedited reporting timescales. SUSARs that are fatal or life-threatening must be notified to the MHRA and REC within 7 days after sponsor awareness. Other SUSARs must be reported to the REC and MHRA within 15 days after sponsor awareness.

## Adverse Event Recording and Processing Flow Chart

**Adverse Event Noted**

**Adverse Event (AE)**

**Serious Adverse Event (SAE)**

**Unrelated to IMP**

**Related to IMP**

**Unrelated to IMP**

**Related to IMP**

**Adverse Event (AE)**

**Adverse Reaction (AR)**

**Serious Adverse Event (SAE)**

**Serious Adverse Reaction (SAR)**

**Seriousness**

**Causality**

**Expectedness**

**Expected SAR**

**Unexpected SAR**

**SUSAR**

**Record in:**

- **Medical Record**
- **CRF***
- **SAE Reporting Form***

**Report to JRO:** [**sae@ucl.ac.uk**](mailto:sae@ucl.ac.uk) **and LCTUSTIMULATE@uclan.ac.uk**

**within 24 hrs**

**Record in:**

- **Medical Record**
- **CRF***

**UCL submit SUSAR to MHRA and REC**

**** AEs/SAEs recorded in CRF / SAE reporting Forms for nested drug trial participants only***

## Notification of deaths (nested drug trial participants only)

All deaths will be reported to the Sponsor within 24 hours of the Research Team becoming aware of it as an SAE.

- All deaths will be reported to the sponsor irrespective of whether the death is related to disease progression, the IMP, or an unrelated event.
- All deaths, including deaths deemed unrelated to the IMP, if they occur earlier than expected, will be reported to the sponsor.

## Pregnancy reporting (nested drug trial participants only)

If a female participant becomes pregnant (or partner of a male participant who has received the IMP colchicine) at any point during their treatment in the trial and up to 30 days from last dose (female participants) or 90 days from last dose (partner of male participant), a completed trial specific Pregnancy Reporting Form will be completed and emailed to the Sponsor at [SAE@ucl.ac.uk](mailto:SAE@ucl.ac.uk) and [**LCTUSTIMULATE@uclan.ac.uk**](mailto:LCTUSTIMULATE@uclan.ac.uk), within 24 hours of the Investigator becoming aware of the event. The Investigator will respond to any queries raised by the Sponsor as soon as possible.

Completed Pregnancy Reporting Forms must be sent to the Sponsor within 24 hours of becoming aware of the event

**Email Pregnancy Forms to:** [**SAE@ucl.ac.uk**](mailto:SAEreporting@uclan.ac.uk) **and LCTUSTIMULATE@uclan.ac.uk**

The Sponsor must be kept informed of any new developments involving the pregnancy through the completion of a follow-up Pregnancy Reporting Form. Any pregnancy that occurs in a female trial subject during a clinical trial should be followed to termination or to term.

Consent to report information regarding the pregnancy include follow-up of a child born if applicable must be obtained from the pregnant participant including the partner if applicable . A trial-specific pregnancy monitoring information sheet and informed consent form for trial participants including the Pregnant Partner of trial participants if applicable must be used for this purpose.

With consent additional information regarding the pregnancy will be collected and reported to the Sponsor, the Sponsor will advise on the length of follow up of the pregnancy / child on a case by case basis.

## Overdose Reporting

All incidences of overdose will be reported on eCRFs and reported as an SAE within 24 hours of the trial team becoming aware of it. The priority for the trial team is to assist with directing patients affected to be treated urgently through standard NHS emergency admission to the nearest Accident and Emergency department for immediate medical attention and treatment. Patient’s GPs will be notified of the incidence and the patient will be monitored under standard of care. The patient will be discontinued on the trial drug but remain in the trial for follow up and assessment at the usual 12 and 24-week assessment.

## Loratadine Overdose

Overdosage with loratadine increased the occurrence of anticholinergic symptoms. Somnolence, tachycardia, and headache have been reported with overdoses.

In the event of overdose, general symptomatic and supportive measures are to be instituted and maintained for as long as necessary. Administration of activated charcoal as a slurry with water may be attempted. Gastric lavage may be considered.

Loratadine is not removed by haemodialysis and it is not known if loratadine is removed by peritoneal dialysis.

Patients will be sign posted for urgent medical attention at the emergency department of the nearest hospital to the patient’s residence. Medical monitoring of the patient is to be continued after hospital treatment.

## Famotidine Overdose

The adverse reactions in overdose cases are similar to the adverse reactions encountered in normal clinical experience (see the adverse reactions table for Famotidine in Appendix I).

No incidence of overdose with Famotidine had been reported so far from clinics.

Patients who had received daily doses of up to 800 mg over a period of one year without exhibiting any significant undesirable effects.

In the event of overdose, the aim should be to remove any unabsorbed drug from the alimentary tract with the usual measures from the gastrointestinal tract, clinical monitoring, and supportive therapy should be employed.

## Colchicine Overdose

Procedures for colchicine overdose will follow those outlined in the SmPC for colchicine. The lethal dose varies widely (7 - 65 mg single dose) for adults but is generally about 20 mg. Therefore, overdose of study colchicine treatment is unlikely during the trial, given the low dose of colchicine (1mg daily)

In the unlikely event of colchicine overdose, all patients, even in the absence of early symptoms, should be referred for immediate medical assessment at an emergency department closest to the patient’s residence for urgent medical treatment.

Symptoms of acute overdosage may be delayed (3 hours on average): nausea, vomiting, abdominal pain, haemorrhagic gastroenteritis, volume depletion, electrolyte abnormalities, leucocytosis, hypotension in severe cases. The second phase with life threatening complications develops 24 to 72 hours after drug administration: multisystem organ dysfunction, acute renal failure, confusion, coma, ascending peripheral motor and sensory neuropathy, myocardial depression, pancytopenia, dysrhythmias, respiratory failure, consumption coagulopathy. Death may result from respiratory depression and cardiovascular collapse. If the patient survives, recovery may be accompanied by rebound leucocytosis and reversible alopecia starting about one week after the initial ingestion.

**Treatment:**

No antidote is available.

Treatment will focus on elimination of toxins by gastric lavage if treatment is begun early (within one hour of acute poisoning). Oral activated charcoal may be of benefit in adults who have ingested more than 0.1mg/kg bodyweight within 1 hour of presentation. Haemodialysis has no efficacy.

Close clinical and biological monitoring in hospital environment will be performed, plus symptomatic and supportive treatment (control of respiration, maintenance of blood pressure and circulation, correction of fluid and electrolytes imbalance.

## Rivaroxaban Overdose

Rare cases of overdose up to 600 mg have been reported without bleeding complications or other adverse reactions. Due to limited absorption a ceiling effect with no further increase in average plasma exposure is expected at supratherapeutic doses of 50 mg rivaroxaban or above.

A specific reversal agent (andexanet alfa) antagonising the pharmacodynamic effect of rivaroxaban is available (refer to the SmPC of andexanet alfa).

The use of activated charcoal to reduce absorption in case of rivaroxaban overdose may be considered.

**Management of bleeding**

In the event of overdose with Rivaroxaban treatment should be discontinued. Rivaroxaban has a half-life of approximately 5 to 13 hours.

Management should be individualised according to the severity and location of the haemorrhage. Appropriate symptomatic treatment could be used as needed, such as mechanical compression (e.g. for severe epistaxis), surgical haemostasis with bleeding control procedures, fluid replacement and haemodynamic support, blood products (packed red cells or fresh frozen plasma, depending on associated anaemia or coagulopathy) or platelets.

If bleeding cannot be controlled by the above measures, either the administration of a specific factor Xa inhibitor reversal agent (andexanet alfa), which antagonises the pharmacodynamic effect of rivaroxaban, or a specific procoagulant reversal agent, such as prothrombin complex concentrate (PCC), activated prothrombin complex concentrate (APCC) or recombinant factor VIIa (r-FVIIa), should be considered. However, there is currently very limited clinical experience with the use of these medicinal products in individuals receiving rivaroxaban. The recommendation is also based on limited non-clinical data. Re-dosing of recombinant factor VIIa shall be considered and titrated depending on improvement of bleeding. Depending on local availability, a consultation with a coagulation expert should be considered in case of major bleedings. Protamine sulphate and vitamin K are not expected to affect the anticoagulant activity of rivaroxaban. There is limited experience with tranexamic acid and no experience with aminocaproic acid and aprotinin in individuals receiving rivaroxaban. There is neither scientific rationale for benefit nor experience with the use of the systemic haemostatic desmopressin in individuals receiving rivaroxaban. Due to the high plasma protein binding rivaroxaban is not expected to be dialysable.

## New Safety Findings

If a new safety finding emerges (from sources such as IMP manufacturers, data analysis, IDMC findings), the CI reviews the finding for its impact on the subjects participating in the relevant trial(s). If there is a potential impact on trial participant’s safety, the Sponsor takes appropriate action in conjunction with the CTM, CI and research team. Appropriate reporting mechanisms are followed in the event of actions being taken.

## Urgent Safety Measures

Where an urgent safety measure (to prevent immediate hazard to trial subject’s health and safety) is necessary, prior authorisations from the MHRA and ethics are not required.

Where the PI takes urgent action that is not consistent with the protocol to prevent harm to a subject on a trial, the PI must immediately inform the CI and the CTM and give full details of the measures taken and the decision-making process surrounding the action(s) taken. The CTM will inform the CI, Sponsor, ethics and the MHRA of these measures immediately, but **no later than 3 days** of the actions being taken.

An amendment is formally submitted as soon as possible by the CI and CTM to the relevant bodies in conjunction with the sponsor.

## Development of Safety Update Reports

The Sponsor will provide the MHRA and REC with Development Safety Update Reports (DSUR) which will be written by the Sponsor’s office in conjunction with the trial team. The report will be submitted within 60 days of the Developmental International Birth Date (DIBD) of the trial each year until the trial is declared ended.

## Responsibilities

### Responsibilities of Principal Investigator / delegates

Checking for AEs and ARs when participants attend for treatment / follow-up.

1. Using medical judgement in assigning seriousness and causality.
2. Ensuring that all SAEs are recorded and reported to the sponsor within 24 hours of becoming aware of the event and provide further follow-up information as soon as available. Ensuring that SAEs are chased with Sponsor if a record of receipt is not received within 3 working days of initial reporting.
3. Ensuring that AEs and ARs are recorded and reported to the sponsor in line with the requirements of the protocol.

### Responsibilities of Chief Investigator / delegates

All of the above responsibilities of a PI, and in addition:

1. Clinical oversight of the safety of participants participating in the trial, including an ongoing review of the risk / benefit.
2. Using medical judgement in assigning the SAEs seriousness and causality where it has not been possible to obtain local medical assessment.
3. Review of all SUSARs.
4. Review of specific SAEs and SARs in accordance with the trial risk assessment and protocol.
5. Reporting safety information to the independent oversight committees identified for the trial including Data Monitoring Committee (DMC) and / or Trial Steering Committee (TSC).
6. Reviewing and contributing to the annual Development Safety Update Report (DSUR).

### Responsibilities of the Sponsor / delegates

1. Data collection and verification of SAEs, SARs and SUSARs according to the trial protocol onto a database.
2. Reporting safety information to the CI or delegate for the ongoing assessment of the risk / benefit.
3. Expedited reporting of SUSARs to the Competent Authority (MHRA in UK) and REC within required timelines.
4. Notifying Investigators of SUSARs that occur within the trial.
5. Checking for and notifying PIs of updates to the Reference Safety Information for the trial.
6. Preparing the DSUR in collaboration with the CI and ensuring timely submission to the MHRA and REC (within 60 calendar days).

# DATA MANAGEMENT AND QUALITY ASSURANCE

In accordance with the principles of Good Clinical Practice and the recommendations and guidelines issued by UK HRA and MHRA, the design, conduct and analysis of this trial is focussed on issues that might have a material impact on the wellbeing and safety of study participants with Long COVID and the reliability of the results that would inform the care for future patients.

Quality Control (QC) includes the operational techniques and activities done within the QA system to verify that the requirements for quality of the trial-related activities are fulfilled. A risk-adapted approach will be used for monitoring. All sites will be centrally monitored for recruitment, data completeness, quality and timeliness of data entry, number of data change requests. A minimum routine remote monitoring schedule of 3 monthly will be set. Any concerns not resolved or raised as a result of the remote monitoring will trigger a full site monitoring visit. To this end:

- Coordinated by LCTU Trial Manager and monitor, site initiation visits will be performed to enable training of local research personnel
- The Trial Manager at LCTU will document the completion of the initiation checklist for each site to verify appropriate approvals are in place prior to initiation of the site
- The Trial Manager will document, as part of the initiation checklist, that all relevant personnel have undergone trial specific training
- Data will be centrally monitored by the LCTU Monitor and Trial Manager to check:
  - Adverse Event reporting rates between centres
  - Screening, recruitment, and dropout rates between centres
  - Data entry consistency. The Data Manager and Trial Manager, with the Monitor, will follow-up on the data queries; Central monitoring reports will thus be generated for the TMG, who will oversee the activity and in accordance with the monitoring plan, will identify when additional intervention, e.g. site visits, should be undertaken.
  - Independent oversight of the trial will be provided by IDMC and independent members of the TSC.

Among the most important factors influencing the delivery of these quality objectives are:

- Minimising the burden on the clinicians working in overstretched Long COVID clinics.
- Ensuring suitability of the participants and having access to the trial treatment without impacting on their other medical needs.
- Ensuring information given to the participants and the PIs in a timely and readily digestible fashion without adversely impacting on the patient care.
- To allow the treating physician to use their clinical judgement to decide whether any of the treatment arms are not suitable for the patient under their care.
- To collect comprehensive information on the mortality as well as morbidity of the Long COVID status.
- In all aspects of the trial, any risks to the patients and well-being will by a key principle in that of proportionality. Risks associated with participation in the trial must be considered in the context of usual care.

# DATA COLLECTION AND SOURCE DOCUMENT IDENTIFICATION

Data will be collected from sites on trial-specific case report forms or data collection tools such as electronic CRFs and electronic documents.

Source data are contained in source documents and must be accurately transcribed on to the paper CRF (pCRF). Examples of source documents are medical records which include laboratory and other clinical reports etc.

A source document list will be implemented and recorded in the Data Management Plan and Monitoring Plan, prior to the start of the trial, to identify:

1. which data is to be recorded directly onto the pCRF
2. which data is recorded firstly into source documents, such as medical notes, laboratory reports or scan reports and then transcribed into the pCRF for entry into the trial database.
3. which data is not to be recorded in the pCRF but only recorded in source documents.
4. Data entry into the eCRF, completing and closing a record
5. Data change requests

The Case Report Forms (CRFs) will not bear the participant’s name or other personal identifiable data.

## Data Collection from Coverscan™

This trial aims to measure the prevalence of organ volume changes and damages in lungs, heart, kidney, liver, pancreas, spleen as assessed by the MRI scanner among those having recovered, or recovering, from the SARS-CoV-2 infection in the cluster randomised trial. Patients who are allocated by PCN deprivation score to receive the Coverscan™, will be referred for the scan 4 weeks prior to their Long COVID clinic appointment. The scan results will be assessed and reported on by a specialist radiologist group supported by Perspectum. Reports will be returned to clinic site and become part of the clinical record. Reports should be available to clinicians at the time of the clinic appointment to inform care decision making. Data will be directly transferred from Perspectum to LCTU for analysis within the trial.

## Data Collection from Long COVID Clinics

Routine standard of care clinical data from patients undergoing investigations at the 6 participating Long COVID clinics will be captured through the patient facing questionnaire pCRF and research staff completed pCRF (clinical data and investigations collected from the clinical record and physical assessment of participant) by a member of the trial team at the site.

## Linked National Data Resource (Healthcare Systems Data)

Participants will be consented for use of NHS data regarding medical history (including hospital and outpatient attendances, prescriptions, and other relevant information) from January 2020 and up to 1 year following enrolment in the study. This data capture will be via NHS Digital using the patient’s NHS number.

## Completing Case Report Forms

All eCRFs and pCRFs must be completed and signed by the research team staff that are listed on the site staff delegation log and authorised by the PI to perform this duty. The PI is responsible for the accuracy of all data reported in the pCRF. The information from pCRF will be entered into an eCRF within the electronic trial database by the site team directly for the baseline and 12 -week assessment (where the participant attends in person). All site team members will have individualised encrypted access to the database allowing for all entries and changes to be seen within the database audit and change log

For virtual clinics and where the patients are not returning to the clinic in person, participants will be given the option of completion via postal, elink (directly into database for self-completion) or over the telephone for participants requiring support.

All the CRFs will be entered into the trial database within 15 days of data received by the site staff or CTU staff as applicable.

## Data Handling and Analysis

Lancashire CTU data management SOP (DM-03 and DM-05) will be followed and a trial specific Data Management Plan will be in place for the trial before opening to data collection. This will contain details of the software to be used for the database, the process of database design, data validation plan, data entry, data quality checks, data queries, data security, database lock.

Where data are transferred electronically this will be in accordance with the UK Data Protection Act 2018 as well as UCL, Lancashire CTU and UCLan Information Security Policy and Trust Information Governance Policy.

There will be a documented record of data transfer and measures in place for the recovery of original information after transfer.

## Access to the final trial dataset

The CI, Sponsor and trial statisticians will have access to the full dataset prior to database lock and data analysis at the end of the trial. Collaborators and the PIs have access to pseudonymised data.

A copy of the data dictionary will be deposited with the Sponsor and a copy archived at LCTU to comply with the regulatory requirement for archiving clinical trials data.

# STATISTICAL CONSIDERATIONS

## Sample Size Calculation – Main study

Motivation: The main study takes the form of a 2x2 factorial trial where the unit of randomisation is the PCN. The sample size calculation was based on the power to detect an interaction effect of clinically important size of 3 points (40) on the FAS scale (41) rather than on the size of the main effects themselves. Therefore, the sizes of the main effects are not specified below. For context, the difference in means of FAS between people who say they have or have not recovered from Long COVID was 9 points in an observational study (41).

The sample size was calculated using PASS v21.0.1(2021) for a 2x2 cluster factorial trial. The study was powered to detect an interaction on the FAS scale between Coverscan™ and Living with COVID Recovery™. The sample size does not depend on the size of main effects of both individual interventions. Based on published data the standard deviation on the FAS scale for patients with Long COVID is estimated at 6 units (see Figure 12 of reference (41). An interaction effect of 3 points (40) on the FAS can be detected with just over 90% power and (two-sided) significance level of 0.05 with 960 participants in 48 PCN clusters of 20 participants, assuming a conservative intra-cluster correlation coefficient (ICC) of 0.02. If there is dropout of 15% (to be conservative) and assuming missingness is roughly equal across the arms, then the number of participants must be inflated by a factor of 100/85 to 1130 (approximately 56.5 centres of 20 participants).

Only if there is no possibility of an interaction effect between the drugs of the platform trial and the main and interaction effects in the 2x2 factorial trial, can the trials be considered independent and data from all arms of the adaptive trial can be combined to assess the effects within the 2x2 factorial trial. Assuming non-independence of the two trials, the total number of participants required for the interaction effect within the factorial trial to be detected with 90% power is 1130 receiving usual care (i.e. not entering the drug trial or randomised to usual care in the drug trial).

Experimental estimates of the prevalence of symptoms that remain 12-weeks after COVID infection range from 3.0% based on tracking specific symptoms, to 11.7% based on self-classification of Long COVID, using data to 1 August 2021 (3). One Long COVID clinic has ~25 new patients per week(16), suggesting there might be around 1300 new patients annually per clinic. Most are expected to give consent to data collection even if not willing to consent to randomisation into a drug study. Long COVID referral rates range from 0-2.5/1000 per PCN. Assuming this rate will not change over the foreseeable future, this will ensure that our 6-10 Long COVID clinics will provide sufficient patients for this study. Moreover, there is a backlog of people waiting for their first Long COVID clinic appointment (from private communication).

## Sample Size Calculation – Nested Drug Trial

The total number of individual drugs tested on the platform is currently unknown.

The total sample size required in an adaptive platform drug trial, with uncertainty in the timing of the introduction of further drugs, cannot be set in advance, but would need to be updated in consultation with the IDMC and funder throughout the trial at set interim points in data collection.

The treatment effect is unknown at this time for the drugs. The plan is to perform interim analysis after 1200 participants in total are recruited and after every 600 participants thereafter. With 200 participants per active arm and corresponding concurrent controls the study has 85% power to detect a Cohen’s D of 0.3 on FAS (difference between means in two treatment arms divided by the standard deviation of the data) at the 0.05 alpha level. If this number is increased to 300 for the same effect size the power is 95%.

## Planned Recruitment Rate

An estimate of the recruitment period for the trial (calculated based on the expected number of eligible and recruited participants available per month/year) with justification that the [required sample size](http://www.sgul.ac.uk/depts/chs/chs_research/stat_guide/trials.cfm#achieving) will be attainable in practice within 24 months. To plan and cost the trial a nominal maximum of 4520 recruited participants was proposed based on up to 4 (drug and usual care) parallel arms of up to 1130 evaluable participants. The nested drug trial aims to recruit up to 4520 patients from a possible pool of 30 000 patients from 6-10 PCN areas of England. The trial will continue to recruit participants at least until the recruitment target of 1130 participants for each of the initial arms of the adaptive trial has been reached. If the adaptive trial requires the full 4520 participants, it is estimated it will take approximately 10 to 12 months for the recruitment to complete and by the 16^th^ month from the start of the trial the last patient last visit would be completed in first quarter of 2023. The projected recruitment rate is 600 participants per month and approximately 100 participants per month per clinic will need to be consented for data collection into the cluster randomised trial.

## Randomisation Methods

Randomisation to the cluster trial will be at the PCN level. Block randomisation for the 226 PCNs will be used with blocks of size 4. There will be two factors in the randomisation, and within each clinic : PCN size (categorised into 2 levels) and PCN deprivation (categorised into 2 levels) (defined in Section 8). Within each clinic, the strata of PCNs will be arranged as larger and more deprived; larger and less deprived; smaller and less deprived; and smaller and more deprived, before randomised allocation into the 4 cluster allocations. The randomisation ratio is 1:1:1:1 between the 4 cluster allocations.

Randomisation to the drug trial will be at the individual level. Block randomisation will be used with varying block sizes. There will be two factors in the randomisation within each clinic: gender (male vs female) and treatment pathway. The randomisation ratio will be equal between all of the drug treatment arms (including usual care) open to recruitment at that point in the adaptive trial.

## Statistical Analysis Plan

The trial will be analysed and reported using the “Consolidated Standard of Reporting Trials‟ (“CONSORT”) and the International Conference on Harmonisation E9 guidelines. The main features of the statistical analysis plan are included here.

## Summary of Baseline Data and Flow of Participants

A CONSORT diagram will be produced to report of the flow of participants in the cluster trial.

There will be a description of screening log data for eligible participants. There will be a further summary of baseline characteristics of all participants that consent to data collection, by frequency and percentage for categorical variables, and for continuous variables by mean and standard deviation (or median and inter-quartile range for non-normally distributed data).

A CONSORT diagram will be produced to report of the flow of participants in all arms of the trial.

Summary of baseline characteristics, by study arms, will be by frequency and percentage for categorical variables, and for continuous variables by mean and standard deviation (or median and inter-quartile range for severely skewed data).

## Primary Outcome Analysis

Primary analysis of the trial will be intention-to-treat (ITT). The primary outcome, FAS at 12 Weeks, is expected to be approximately normally distributed (40). A multi-level model analysis will be used to evaluate effects of Coverscan™, digitally enabled community rehabilitation, and their interaction, on FAS at 12 weeks adjusting for baseline FAS, with clinic and PCN as random effects.

The principal analysis population for these estimates of the effects of Coverscan™, digitally enabled community rehabilitation, and their interaction, will be those participants (up to 1130) who consented to data collection for the cluster RCT and were allocated to usual care (either by randomisation to usual care in the drug trial or being willing for data collection, but not randomised into the drug trial). This analysis will provide the most straightforward answer to the primary objective in the broadest group of participants.

A similar and more pragmatic multi-level model analysis of the effects of Coverscan™, digitally enabled community rehabilitation, and their interaction, will be applied to all participants who consent to data collection in the cluster trial regardless of participation in the drug trial. This analysis will provide an answer to the primary objective in the presence of other treatments including drug treatments and will allow comparison to the results from the principal analysis set.

The principal analysis population for drug vs. usual care comparisons within the drugs trial will be the participants randomised to the control treatment pathway, i.e. no Coverscan + usual care rehabilitation. A multi-level model for FAS, adjusting for baseline, with gender as a fixed effect and clinic and PCN as random effects, will be applied to the drug trial data. The total population of participants who are randomised into the drugs trial will be used to study the effects of the drugs in combination/interaction with the pathways. A multi-level model for FAS, adjusting for baseline, with gender as a fixed effect and clinic and PCN as random effects, will be applied to data from all participants of both the cluster trial and the drug trial to evaluate effects of Coverscan™, digitally enabled community rehabilitation, and their interaction, in combination with the participants’ allocated drug treatment. For all drug treatments, particularly any introduced after the start of the trial, comparisons with participants randomised to usual care will include only those participants who could have been randomised to the relevant drug treatment at the time of their randomisation. This will reduce the potential for bias and have the effect of ensuring that only contemporaneous participants allocated to usual care are compared to participants randomised to drug treatments.

Patterns of missingness will be summarised. In modelling, complete case analysis will be the main analysis. For missing outcome data, we will use a threshold approach as a sensitivity analysis. Imputation based on thresholds will be implemented such that missing values will be replaced by values from the lower quantiles of the distribution, e.g. 25^th^ quartile, 10^th^ quartile, and minimum. Further details will be provided in the Statistical Analysis Plan.

## Secondary Outcome Analysis

Secondary outcomes on an interval scale will be analysed similarly to the primary outcome, adjusting for baseline outcome measure, clinic and PCN as a random effect. The principal analysis set for estimates of effects of Coverscan™, digitally enabled community rehabilitation, and their interaction, on these secondary outcomes (EQ-5D-5L, GAD-7, PHQ-9, WSAS, PDQ-5, CFQ, SF-12, MRC Dyspnoea score) being participants who consented to data collection for the cluster RCT and were allocated to usual care (either by randomisation in the drug trial or being willing for data collection, but not randomisation into the drug trial). Where appropriate, alternative methods will be applied to outcomes with skewed distributions. Secondary outcomes on an interval scale will be compared between drug treatment arms using the same methods as for the primary outcome, using a multi-level model for the relevant outcome, adjusting for baseline, with gender as a fixed effect and clinic and PCN as random effects, within the control treatment pathway.

Patterns of missingness will be summarised. In modelling, complete case analysis will be done as the main analysis. For missing outcome data, we will use a threshold approach to imputation and sensitivity analyses.

Health outcomes and health utilisation data taken from medical records at 12 months will be described using summary statistics for all participants and for subgroups defined by pathophysiology at baseline.

## Sensitivity and other planned analysis

A sensitivity analysis of the effects of Coverscan™, digitally enabled community rehabilitation, and their interaction, will include only those who consent to data collection for the cluster trial and are randomly allocated to receive usual care.

If the Living with COVID Recovery™ application is found to be beneficial, further exploratory analyses will explore factors associated with greater benefit. Some participants allocated to this application will not be able to access it as they lack a suitable mobile phone or tablet. Other participants will have suitable access but may choose to interact with it on a more or less extensive basis. Amount of access will be available as a patient reported variable and as summary variable from within the application.

Subgroup analyses of clinical sub-phenotype, including (but not limited to) cardiorespiratory, neuropsychiatric and "mast-cell activation type" (including rashes, joint pain and gastrointestinal disturbances and serositis symptoms) will be included in the Statistical Analysis Plan. These subgroups are not mutually exclusive as some participants may belong to more than one subgroup depending on their symptoms.

## Interim Analysis

The first interim analysis point will be after 1200 participants are recruited to the cluster randomised trial and the remaining interim points will be after every 600 participants recruited. The interim analysis time points will apply to both the cluster and drug trials.

The interim analysis of the cluster interventions will take place among the participants receiving usual care only (by allocation in the drug trial or by refusing or being ineligible for the drug trial). A pathway (a combination of the two cluster interventions) could be found to be superior after the first 1200 participants and every 600 thereafter (5 interim analysis) participants in the study. A statistically significant p-value at the 0.005 two-sided level is required for a pathway to be deemed superior at interim 1 to 5. This is to account for multiple testing (Bonferroni type correction). The analysis at 4520 patients in total will be at the conventional 0.05 two-sided level. There are practical and operational reasons why a single pathway cannot graduate and become the standard pathway within the recruitment period of this trial.

For a drug to graduate early and become part of the backbone treatment of the trial, its two-sided p-value at each of the interim time points (interim 1 to 5) will need to be ≤ 0.005. The final analysis for each drug, including only those participants in the standard of care cluster pathway, will be with at least 200 participants in the active arm and 200 concurrent controls, and will use the conventional 2-sided 0.05 level. This analysis has 85% power if Cohen’s D is 0.3 and 97% power if Cohen’s D is 0.4.

# END OF TRIAL

The study is deemed to have ended following the last data collection point within the trial. This will be 12 months following enrolment of the last participant in the trial.

Thereafter, there will be a 6-month period for data analysis and reporting.

The CI and/or TSC have the right at any time to terminate the trial for clinical or administrative reasons. The funder may withdraw funding in the event of futility, study misconduct, or other unanticipated events. In such instances, the Sponsor will notify the MHRA within 15 days.

The end of the trial will be reported to the REC and Regulatory Authority within the required timeframe if the trial is terminated prematurely. Investigators will inform patients of any premature termination of the trial and ensure that the appropriate follow up is arranged for all involved.

Following the end of the trial a summary report of the trial will be provided to the REC and Regulatory Authority within the required timeframe. The Sponsor will notify MHRA at the end of the clinical trial within 90 days of its completion.

# RECORD KEEPING AND ARCHIVING

At the end of the trial, all essential documentation and the trial dataset will be prepared for archiving and transfer to the Sponsor organisation by Lancashire CTU. Participating sites will be asked to archive for a minimum of 25 years from the declaration of end of trial. Essential documents are those which enable both the conduct of the trial and the quality of the data produced to be evaluated and show whether the site complied with the principles of Good Clinical Practice and all applicable regulatory requirements.

The Sponsor will notify sites when trial documentation can be archived. All archived documents must continue to be available for inspection by appropriate authorities upon request.

# OVERSIGHT COMMITTEES

There are three main trial management groups which are involved in the set up and management of STIMULATE-ICP.

The roles and responsibilities for each of these committees are outlined in the terms of reference charter for each committee and are provided in separate documents. Membership of each committee is dependent on accepting the terms of reference and declaration of conflict of interest. Copies of the signed copies of the terms of reference, and the declaration of conflict of interest will be stored in the TMF.

## Trial Management Group (TMG)

The TMG will include the Chief Investigator and trial staff. The TMG will be responsible for overseeing the trial. Representatives from public and patient engagement program is a member of the TMG group.

The TMG will review Recruitment figures, SAEs and substantial amendments to the protocol prior to submission to the REC and/or MHRA. All PIs will be kept informed of substantial amendments through their nominated responsible individuals*.*

The Trial Management Group should meet regularly on a monthly basis, to ensure all practical details of the trial are progressing well and working well and everyone within the trial understands them and will send updates to the PIs.

Members of the TMG must abide by the TMG membership charter which is supplied to the members in a separate document and must also declare any conflict of interest before admission to the group.

## Trial Steering Committee (TSC)

The role of the TSC is to provide overall supervision of the trial. This includes members of the Trial Management Group, plus independent members not otherwise involved in the trial, including the chair of the TSC. The TSC will advise the TMG, particularly with a view to major trial issues, and will review the recommendations of the (Independent) Data Monitoring Committee and, on consideration of this information, recommend any appropriate amendments/actions for the trial as necessary. The TSC should consider the requirements of the Sponsor and funder(s) as well as the trial and its participants and potential future participants.

## Independent Data Monitoring Committee (IDMC)

The role of the IDMC is to provide independent advice on accumulating comparative data and safety aspects of the trial. The Committee will meet before the trial starts recruiting and then every 12 Weeks, until the end of data analysis, to review the trial conduct and data from interim analyses, or as necessary to address any issues. The IDMC is advisory to the TSC and Sponsor and can recommend premature closure of the trial to the TSC.

During the study, interim analyses of all study data will be supplied in strict confidence to the IDMC. The IDMC will schedule reviews/meetings according to the emerging data from this and other studies. The IDMC will independently evaluate these analyses and any other information considered relevant. The IDMC will determine if, in their view, the randomised comparisons in the study have provided evidence on mortality that is strong enough (with a range of uncertainty around the results that is narrow enough) to affect treatment strategies. In such a circumstance, the IDMC will inform the TSC who will make the results available to the Sponsor and, if deemed acceptable by the Sponsor and the funder, amend the trial arms accordingly

## Stopping Rules

The trial may be stopped before completion for the following reasons:

- On the recommendation of the TSC or IDMC (see section 13.3)
- On the recommendation of the Sponsor and CI

All safety data will be reviewed and a decision on continuation will be made by the IDMC with input from the Sponsor.

A single arm of the drug study may be stopped by the IDMC on the grounds of harm or futility at each stopping point.

Additionally, the IDMC may recommend a single arm of the drug study be halted in the event of the death of a single participant that is directly attributable to the study drug.

# MONITORING, AUDIT, AND INSPECTION

The Sponsor has delegated the responsibility for monitoring the STIMULATE-ICP trial to the Lancashire -CTU. An appropriate level of monitoring will be assigned to the trial in conjunction with the outcome of the trial Risk Assessment. Risk will be assessed on an ongoing basis and adjustments will be made accordingly.

The degree of monitoring will be proportionate to the objectives, purpose, phase, design, size, complexity, endpoints and risks associated with the trial. A Monitoring Plan will be prepared by the Lancashire CTU monitoring team, in which the frequency and extent and nature of the monitoring will be outlined, and the trial will be monitored in accordance with this agreed plan.

## Direct Access to Source Data

Each participating site should maintain appropriate medical and research records for this trial, in compliance with International Conference on Harmonisation – E6- Good Clinical Practice Guidelines Section 4.9 and regulatory and institutional requirements for the protection of confidentiality of participants. The investigator(s)/ institution(s) will permit trial-related monitoring, audits, REC review, and regulatory inspection(s), providing direct access to source data/documents. Trial participants are informed of this during the informed consent discussion. Participants will consent to provide access to their medical notes.

Source data will be identified and recorded in the trial Monitoring Plan.

# ETHICS AND REGULATORY REQUIREMENT REPORTING

Lancashire CTU will ensure that the trial protocol and associated documents, have been approved by the regulatory bodies (HRA and MHRA). Before the start of the trial, approval will be sought from a REC for the trial protocol and other relevant documents e.g. Participant Information Sheet, advertisements and GP information letters. The protocol, all other supporting documents including any agreed amendments, will be documented and submitted for ethical and regulatory approval as required. Amendments will not be implemented prior to receipt of the required approval(s).

Before the sites may be opened to recruit participants, Lancashire CTU must receive NHS permission, in writing, from the Trust Research & Development (R&D) to confirm capability and capacity. It is the responsibility of the Lancashire CTU to ensure that all subsequent amendments gain the necessary approvals that these are relayed to the sites and confirmation of update into SIF received. This does not affect the individual clinician’s responsibility to take immediate action if thought necessary to protect the health and interest of individual participants (see section for reporting urgent safety measures).

Within 90 days after the end of the trial, the CI/Sponsor will ensure that the main REC and the MHRA are notified that the trial has finished. If the trial is terminated prematurely, those reports will be made within 15 days after the end of the trial.

Substantial amendments that require review by REC will not be implemented until the REC and MHRA grants a favourable opinion for the trial and/or NHS R&D department before they can be implemented in practice at participating sites.

All correspondence with the REC /MHRA will be retained in the Trial Master File.

## Peer Review

This protocol has been reviewed by independent experts and the reviews were proportionate:

1. The Proposal underwent peer review as part of the NIHR funding application process.
2. At least two individual experts have reviewed the protocol and the funding application. The definition of independent used here is that the reviewers were external to the Chief Investigators’ host institution and not involved in the trial in any way. Reviewers were not anonymous.
3. The protocol has been reviewed by experts in statistical analysis and clinical trials methodology.
   1. **Public and Patient Involvement**

The research proposal has been enriched by existing robust patient and public involvement and engagement (PPIE) using multiple channels, including regular updates and webinars (Coverscan™ study), surveys (Long COVID SOS), social media (direct engagement and indirect analysis), and working with ethnic minorities. The proposal for this protocol has been informed by existing engagements with patients in research, clinical practice, policy such as RCGP PPI unit, lived experience or through organisations, such as Long COVID SOS and UK Doctors #Long COVID. Institutions and collaborators have established PPIE systems which will be involved throughout.

Patients have been central in setting research questions, designing, writing and organisation, including focus on care pathways, improved diagnostics, pragmatic trials, new treatments, health inequalities and mental health.

Members of the PPIE will be engaged with the trial conduct and review the trial documentations, assist with amendments and publications of the patient facing documents before submission for regulatory approval. They also assist with review and feedback on the trial literature, adverts and for the purpose of recruitment and enrolment of the participants in the trial. Public and patients will be involved in other stakeholder engagement and the ultimate policy recommendation on publication including the dissemination plans.

- 1. **Regulatory Compliance**

The trial will not commence until a Clinical Trial Authorisation (CTA) is obtained from the MHRA and favourable REC opinion. The protocol and trial conduct will comply with the Medicines for Human Use (Clinical Trials) Regulations 2004 (SI.2004/1031) and any relevant amendments as well as all applicable national and local laws and regulations.

### Protocol Compliance

Prospective, planned deviations or waivers to the protocol are not allowed under the UK regulations on Clinical Trials and must not be used e.g. it is not acceptable to enrol a participant if they do not meet the eligibility criteria or restrictions specified in the trial protocol.

Accidental protocol deviations can happen at any time. They will be adequately documented on the relevant forms and reported to the Chief Investigator and Sponsor immediately, as per Sponsor SOP for the Recording & Reporting of Deviations, Violations, Potential Serious breaches, Serious breaches and Urgent Safety Measures (SPON/S15).

Deviations from the protocol which are found to frequently recur are not acceptable, will require immediate action and could potentially be classified as a serious breach.

### Notification of Serious Breaches to GCP and Protocol

A “serious breach” is a breach which is likely to effect to a significant degree –

(a) the safety or physical or mental integrity of the participants of the trial; or

(b) the scientific value of the trial.

The Sponsor will be notified immediately of any case where the above definition applies during the trial conduct phase as per the Sponsor SOP for the Recording & Reporting of Deviations, Violations, Potential Serious breaches, Serious breaches, and Urgent Safety Measures. The Sponsor will notify the licensing authority in writing of any serious breach of:

1. the conditions and principles of GCP in connection with the trial; or
2. the protocol relating to the trial, as amended from time to time, within 7 days of becoming aware of that breach

### Safety Reporting

SAE and SUSAR reporting to the Sponsor, IDMC and regulatory authorities will be done according to the details outlined in Section 11.3 and 11.6 of this protocol.

### Annual Progress Report

An annual progress report (APR) will be submitted to the REC within 30 days of the anniversary date on which the favourable opinion was given, and annually until the trial is declared ended. APR is produced by the LCTU, but it is the Chief Investigator’s responsibility to review and authorise the release of the annual reports as required.

### End or Trial Report

At the end of the trial the CI will supply the Sponsor with a clinical trial report and upload the trial results to all applicable trial registries within 12 months of the end of trial declaration date. A copy of this report will also be submitted to the REC and MHRA.

- 1. **Data Protection and Participant Confidentiality**

All data will be handled in accordance with the Data Protection Act (2018). Participant confidentiality will be maintained, and the trial will be compliant with the requirements of the Act.

Electronic and paper Case Report Forms will be labelled with unique patient identifier allocated upon randomisation/registration. Medical information may be given to the participant’s medical team and all appropriate medical personnel responsible for the participant’s welfare. UCL and LCTU will preserve the confidentiality of participants taking part in the trial.

UCL as Sponsor is the Data Controller as defined under the Data Protection Act 2018. The University of Central Lancashire is acting as a data processor when handling participants data.

All investigators and trial site staff must comply with the requirements of the Data Protection Act with regards to the collection, storage, processing, and disclosure of personal information and will uphold the Act’s core principles.

Personal information collected by the participating site staff will be kept secure and maintained which involves:

- Each participant is given a coded number to replace the participant’s identifiable information.
- The information which links the patient identifiable information and the randomisation code will be kept secure and kept in separate locations using encrypted digital files within password protected folders and storage media.
- Access will be limited to the minimum number of individuals necessary for quality control, audit, and analysis.
- Data transmitted to the Sponsor in pseudonymised fashion, with all patient identifiable information removed prior to transfer.

The participant’s initials, date of birth and trial identification number will be used for identification and this will be clearly explained to the patient in the Patient Information Sheet. Patient consent for this will be sought.

Any breach of participants personal data (e.g. name or date of birth being sent to an unauthorised individual) must be reported to Lancashire CTU immediately or within 24 hours of the site become aware of the incidence. Lancashire CTU will notify the Sponsor within 24 hours of becoming aware of the incidence for follow-up and reporting (if applicable) as part of data controller responsibilities. The data protection office of both organisations will determine the outcome of the breach and necessary root cause analysis and actions points will be put in place to prevent such recurrence.

The Case Report Forms (CRFs) will not bear the participant’s name or other personal identifiable data.

The following personal identifiable information will be collected from patients and sites by LCTU to enable contact with the patient and follow-up of questionnaires and data where required, this will be specified within the participant information sheet and consent form:

Consent forms (containing patient name and signature)

NHS number

Address

Name

DOB

Telephone

Email

- Contacting patients

The blood samples gifted by the participants for research and storage will be sent to Perspectum (collaborator) without patient identifiable information, for analysis by external laboratories mentioned in section 9.6. The manner by which the CRFs and the blood samples are handled will be clearly described in the Participant Information Sheet and Consent Form.

- 1. **Financial and other competing interests for the Chief Investigator & PIs at each site**

The trial is funded by a grant from the National Institute for Health Research.

Professor Amitava Banerjee is Chief Investigator for the STIMULATE-ICP clinical trial and has no relevant conflicts of interest in relation to the conduct and analysis of data for this trial. He has received unrelated research grants from AstraZeneca Pharmaceuticals, NIHR, British Medical Association and other funding organisations. He is Trustee of the South Asian Health Foundation.

Dr Rajarshi Banerjee is CEO of Perspectum which developed Coverscan™. Perspectum is providing the Coverscan™ intervention in this trial, central laboratory services and sample analysis and subsequent reporting of study data to Sponsor and Sites. Perspectum will create and maintain a sample biobank for future research for which they will have the right to agree a licence to access samples for their own commercial and research use. Perspectum are receiving funding from the NIHR grant to part cover scanning services, but the provision of central laboratory services and some scanning services will be provided in kind.

Professor Elizabeth Murray leads an NIHR grant which is evaluating “Living with COVID Recovery™” (<https://www.ucl.ac.uk/healthcare-engineering/covid-19/covid-19-engineering-research/living-covid-recovery>), and is not involved in trial conduct, data collection or analysis.

### Payments to participants

The funding for the trial does not include reimbursement for participant time and travel to the Long COVID clinics. The appointment to Coverscan™ and at the Long COVID clinics are usual care, as such no funding is available to support participants in the trial. In areas where support to attend appointments is available e.g. volunteer patient services these will be accessed.

- 1. **Insurance and Indemnity**

The Sponsor (University College London) is an educational establishment and holds insurance against claims from participants for injury caused by their participation in the clinical trial*.* Participants may be able to claim compensation if they can prove that UCL has been negligent. However, as this clinical trial is being carried out at NHS sites, the NHS sites continue to have a duty of care to the participant of the clinical trial. University College London does not accept liability for any breach in the NHS duty of care, or any negligence on the part of NHS employees. This applies whether the hospital is an NHS Trust or otherwise.

Participants may also be able to claim compensation for injury caused by participation in this clinical trial without the need to prove negligence on the part of University College London or another party. Participants who sustain injury and wish to make a claim for compensation should do so in writing in the first instance to the Chief Investigator, who will pass the claim to the Sponsor’s Insurers, via the Sponsor’s office.

Hospitals and Long COVID clinics selected to participate in this clinical trial shall provide NHS standard indemnity against clinical negligence for harm caused by their employees and a copy of the relevant insurance policy or summary shall be provided to University College London, upon request.

# PUBLICATION & DISSEMINATION

## Publication Policy

CONSORT Guidelines and checklist will be reviewed prior to generating any publications for the trial to ensure they meet the standards required for submission to high quality peer reviewed journals etc. <http://www.consort-statement.org/>

Following completion of the study, the Chief Investigator is expected to publish the results of this research in a scientific journal. The International Committee of Medical Journal Editors (ICMJE) member journals have adopted a trials-registration policy as a condition for publication. This policy requires that all clinical trials be registered in a public trials registry. This trial has been registered with [ClinicalTrials.gov](http://www.clinicaltrials.gov), which is sponsored by the National Library of Medicine.

Trial investigators have the right and responsibility to separately communicate their findings to the scientific community and to the public. All such publications shall adhere to the publication policy detailed in Lancashire CTU SOP TM-05 (Trial Reporting), must not be before the reporting of the main results, and will be submitted, at least 30 days prior to submission, for review by the Sponsor, all appropriate Trial Committees and Funder.

The objectives of the publication policy are:

1. To assure and expedite orderly and timely presentations to the scientific community of all pertinent data resulting from the trial

2. To assure scientifically accurate presentation and papers from investigators

3. To assure that all investigators, particularly those of junior rank, have the opportunity to participate and be recognized in the trial-wide presentations and publications

4. To assure that press releases, interviews, presentations, and publications of trial materials are accurate and do not compromise the scientific integrity of this collaborative trial

5. To establish procedures that allows the Trial Steering Committee and the Independent Data and Safety Monitoring Board to review publications and presentations in a timely manner

6. To maintain a complete up-to-date list of presentations and publications, and to distribute such lists to all investigators.

7. To clarify and ensure proper acknowledgement of National Institutes for Health Research and non-National Institutes that supports the trial including all collaborator institutions.

## Open Access and Data Sharing

This trial is funded by NIHR and as a condition of funding, publications from the outcome of the trial must be made accessible through publications in peer reviewed and open access journals.

The trial will adhere to the NIHR Open Access Policy regarding accessibility and data sharing. Details of the policy is published and updated on the NIHR website; <https://www.nihr.ac.uk/documents/nihr-open-access-policy/28999>. The policy does not require that the data must be made open.

The findings from STIMULATE-ICP will be published in journals that makes the analysis available using Creative Commons Attribution (CCBY) licences and allow depositing of the final published version in public repositories without restriction on re-use. A copy of the final manuscript will also be deposited with Europe PMC (<https://europepmc.org/>) upon acceptance for publication which will be made freely available as soon as possible and in any event within 6 months of the Journal publisher’s official date of final publication.

Sharing of the trial data must protect the confidentiality and privacy of participants; respect the terms of consent by participants who are involved in the trial; be consistent with relevant legal, ethical and regulatory frameworks; and guard against unreasonable costs.

Release of the anonymized trial data will be subject to data sharing agreement between the Sponsor, CI and the third party requesting the data. The integrity of the data must always be preserved, and the agreement should be aligned with the Sponsor and Lancashire CTU SOPs on data sharing.

## Authorship Eligibility

The trial will follow the recommendations for the Conduct, Reporting, Editing, and Publication of Scholarly Work in Medical Journals by the International Committee of Medical Journal Editors (ICMJE) in relation to authorship credit that should be based only on:

1. Substantial contributions to the conception or design of the work; or the acquisition, analysis, or interpretation of data for the work; AND

2. Drafting the work or revising it critically for important intellectual content; AND

3. Final approval of the version to be published; AND

4. Agreement to be accountable for all aspects of the work in ensuring that questions related to the accuracy or integrity of any part of the work are appropriately investigated and resolved.

All those designated as authors should meet all four criteria for authorship and all those who meet the four criteria should be identified as authors. All those who do not meet all four criteria should be acknowledged.

Full details of the recommendation are available on (<http://www.icmje.org/>).

# REFERENCES

1. C1248-national-guidance-post-covid-syndrome-assessment-clinics-v2.pdf.

2. Campbell H et al. Integrated care pathways. BMJ. 1998.pdf.

3. Office of National Statistics. <Prevalence of ongoing symptoms following coronavirus (COVID-19) infection in the UK 5 August 2021.pdf>. 2021.

4. Al-Aly Z, Xie Y, Bowe B. High-dimensional characterization of post-acute sequelae of COVID-19. Nature. 2021;594(7862):259-64.

5. Mandal S, Barnett J, Brill SE, Brown JS, Denneny EK, Hare SS, et al. 'Long-COVID': a cross-sectional study of persisting symptoms, biomarker and imaging abnormalities following hospitalisation for COVID-19. Thorax. 2021;76(4):396-8.

6. Maxwell E. Living-with-Covid-Themed-Review-October-2020.pdf. 2020.

7. R H. <Offline COVID-19 is not a pandemic Lancet.pdf>. Lancet. 2020;396(874).

8. Banerjee A, Pasea L, Harris S, Gonzalez-Izquierdo A, Torralbo A, Shallcross L, et al. Estimating excess 1-year mortality associated with the COVID-19 pandemic according to underlying conditions and age: a population-based cohort study. The Lancet. 2020;395(10238):1715-25.

9. Greenhalgh T, Knight M, A'Court C, Buxton M, Husain L. Management of post-acute covid-19 in primary care. BMJ. 2020;370:m3026.

10. NICE. <covid19-rapid-guideline-managing-covid19>. 2021.

11. Jacobson TA, Smith LE, Hirschhorn LR, Huffman MD. Using implementation science to mitigate worsening health inequities in the United States during the COVID-19 pandemic. Int J Equity Health. 2020;19(1):170.

12. Glasgow. <The RE-AIM framework for evaluating interventions what canit tell us about approaches to chronic illness management.pdf>. 2001.

13. McNulty M, Smith JD, Villamar J, Burnett-Zeigler I, Vermeer W, Benbow N, et al. Implementation Research Methodologies for Achieving Scientific Equity and Health Equity. Ethn Dis. 2019;29(Suppl 1):83-92.

14. Moore GF, Audrey S, Barker M, Bond L, Bonell C, Hardeman W, et al. Process evaluation of complex interventions: Medical Research Council guidance. BMJ. 2015;350:h1258.

15. Dennis A, Wamil M, Alberts J, Oben J, Cuthbertson DJ, Wootton D, et al. Multiorgan impairment in low-risk individuals with post-COVID-19 syndrome: a prospective, community-based study. BMJ Open. 2021;11(3):e048391.

16. Heightman M, Prashar J, Hillman TE, Marks M, Livingston R, Ridsdale H, et al. Post-COVID assessment in a specialist clinical service: a 12-month, single-centre analysis of symptoms and healthcare needs in 1325 individuals. medRxiv. 2021:2021.05.25.21257730.

17. Carson G. Research priorities for Long Covid: refined through an international multi-stakeholder forum. BMC Med. 2021;19(1):84.

18. Mandal S, Barnett J, Brill SE, Brown JS, Denneny EK, Hare SS, et al. 'Long-COVID': a cross-sectional study of persisting symptoms, biomarker and imaging abnormalities following hospitalisation for COVID-19. Thorax. 2021;76(4):396-8.

19. Society R. Long Covid what is it and what is needed.pdf.

20. Ayoubkhani D, Khunti K, Nafilyan V, Maddox T, Humberstone B, Diamond I, et al. Post-covid syndrome in individuals admitted to hospital with covid-19: retrospective cohort study. BMJ. 2021;372:n693.

21. Davis HE, Assaf GS, McCorkell L, Wei H, Low RJ, Re'em Y, et al. Characterizing long COVID in an international cohort: 7 months of symptoms and their impact. EClinicalMedicine. 2021:101019.

22. Noor NM, Pett SL, Esmail H, Crook AM, Vale CL, Sydes MR, et al. Adaptive platform trials using multi-arm, multi-stage protocols: getting fast answers in pandemic settings. F1000Res. 2020;9:1109.

23. Parmar MKB, Carpenter J, Sydes MR. More multiarm randomised trials of superiority are needed. The Lancet. 2014;384(9940):283-4.

24. Parmar MK, Barthel FM, Sydes M, Langley R, Kaplan R, Eisenhauer E, et al. Speeding up the evaluation of new agents in cancer. J Natl Cancer Inst. 2008;100(17):1204-14.

25. Parmar MK, Sydes MR, Cafferty FH, Choodari-Oskooei B, Langley RE, Brown L, et al. Testing many treatments within a single protocol over 10 years at MRC Clinical Trials Unit at UCL: Multi-arm, multi-stage platform, umbrella and basket protocols. Clin Trials. 2017;14(5):451-61.

26. Heightman M, Prashar J, Hillman TE, Marks M, Livingston R, Ridsdale H, et al. Post-COVID assessment in a specialist clinical service: a 12-month, single-centre

analysis of symptoms and healthcare needs in 1325 individuals. 2021.

27. Mura C, Preissner S, Nahles S, Heiland M, Bourne PE, Preissner R. Real-world evidence for improved outcomes with histamine antagonists and aspirin in 22,560 COVID-19 patients. Signal Transduction and Targeted Therapy. 2021;6(1):267.

28. Glynne P, Tahmasebi N, Gant V, Gupta R. Long-COVID following mild SARS CoV-2 infection: characteristic T cell alterations and response to antihistamines. medRxiv. 2021:2021.06.06.21258272.

29. Malone RW, Tisdall P, Fremont-Smith P, Liu Y, Huang XP, White KM, et al. COVID-19: Famotidine, Histamine, Mast Cells, and Mechanisms. Res Sq. 2020.

30. Malone RW. More Than Just Heartburn: Does Famotidine Effectively Treat Patients with COVID-19? Dig Dis Sci. 2021;66(11):3672-3.

31. Terman JM, Awsumb JM, Cotler J, Jason LA. Confirmatory factor analysis of a myalgic encephalomyelitis and chronic fatigue syndrome stigma scale. J Health Psychol. 2020;25(13-14):2352-61.

32. Broz P, Dixit VM. Inflammasomes: mechanism of assembly, regulation and signalling. Nat Rev Immunol. 2016;16(7):407-20.

33. Dalbeth N, Lauterio TJ, Wolfe HR. Mechanism of action of colchicine in the treatment of gout. Clin Ther. 2014;36(10):1465-79.

34. Adler Y, Charron P, Imazio M, Badano L, Baron-Esquivias G, Bogaert J, et al. 2015 ESC Guidelines for the Diagnosis and Management of Pericardial Diseases. Rev Esp Cardiol (Engl Ed). 2015;68(12):1126.

35. Kotecha T, Knight DS, Razvi Y, Kumar K, Vimalesvaran K, Thornton G, et al. Patterns of myocardial injury in recovered troponin-positive COVID-19 patients assessed by cardiovascular magnetic resonance. Eur Heart J. 2021.

36. Malas MB, Naazie IN, Elsayed N, Mathlouthi A, Marmor R, Clary B. Thromboembolism risk of COVID-19 is high and associated with a higher risk of mortality: A systematic review and meta-analysis. EClinicalMedicine. 2020;29:100639.

37. Marco A, Marco P. Von Willebrand factor and ADAMTS13 activity as clinical severity markers in patients with COVID-19. J Thromb Thrombolysis. 2021.

38. Indraratna PL, Virk S, Gurram D, Day RO. Use of colchicine in pregnancy: a systematic review and meta-analysis. Rheumatology (Oxford). 2018;57(2):382-7.

39. UCL. New rehabilitation app to aid recovery of COVID-19 ‘long-haulers’. : UCL; 2020 [cited 2020. Available from: <https://www.ucl.ac.uk/news/2020/aug/new-rehabilitation-app-aid-recovery-covid-19-long-haulers>.

40. de Kleijn WP, De Vries J, Wijnen PA, Drent M. Minimal (clinically) important differences for the Fatigue Assessment Scale in sarcoidosis. Respir Med. 2011;105(9):1388-95.

41. Davis HE, Assaf GS, McCorkell L, Wei H, Low RJ, Re’em Y, et al. Characterizing Long COVID in an International Cohort: 7 Months of Symptoms and Their Impact. 2021.

42. <2020_09_HMA_CTFG_Contraception_guidance_Version_1.1_updated.pdf>.

43. Trussell J. Contraceptive failure in the United States. Contraception. 2011;83(5):397-404.

# APPENDICES

#

# Appendix I

**Table 10:** **List and frequency of important side effects from the trial IMPs and mitigating risk plans**

Very common > 1/10, Common > 1/100 and < 1/10, Uncommon > 1/1000 and < 1/100, Rare > 1/10 000 and < 1/1000, Very rare < 1/10 000, Not known (cannot be estimated from the available data)

**Sources:** SmPC Clarityn (loratadine) 10mg tablets (Bayer plc)

SmPC Famotidine 40mg tablets (Tillomed Laboratories Ltd)

SmPC Colchicine Tablets BP 500 mcg (Wockhardt UK Ltd)

SmPC Xarelto (rivaroxaban) 10 mg film-coated tablets (Bayer plc)

| System Organ Class | Adverse Reaction and Frequency | | Clinical risk | | Symptoms to report | | | Management |
| --- | --- | --- | --- | --- | --- | --- | --- | --- |
| Loratadine 10mg tablets | | | | | | | | |
| Immune system disorders | Very rare: Hypersensitivity reactions (including angioedema and anaphylaxis) | | Medically serious = anaphylaxis & angioedema | | Itch, swelling, shortness of breath, rash | | | Stop drugs, dial 999 in case of anaphylactic shock reaction & follow standard anaphylactic protocols. Refer to ED if evidence of oedema |
| Nervous system disorders | Very rare: Dizziness, convulsion | | Medically important (convulsion) | | Seizure, absence attack, associated with memory loss, incontinence, and possible injury | | | Stop drugs, refer to ED if acute evidence of seizure, refer to neurology if seizures suspected but not witnessed |
| Cardiac disorders | Very rare: Tachycardia, palpitation | | Minor | | Dizziness, palpitations | | | Consider stop drugs, GP review, check ECG & blood pressure |
| Gastrointestinal disorders | Very rare: Nausea, dry mouth, gastritis | | Minor | | Nausea, drug mouth, abdominal pain, acid reflux | | | Symptomatic management, stop drugs if intolerable |
| Hepatobiliary disorders | Very rare: Abnormal hepatic function | | Important | | Fatigue, fever, weakness, malaise | | | Check liver function, if ALT >5 times upper limit of normal stop drugs and refer to SDEC for review |
| Skin and subcutaneous tissue disorders | Very rare: Rash, alopecia | | Minor | | Rash, hair loss | | | Stop drugs, monitor, consider dermatology referral if persistent after stopping drugs |
| General disorders | Very rare: Fatigue Not known: Weight increased | | Minor | | Fatigue, weight gain | | | Symptomatic management, stop drugs if intolerable |
|  |  | |  | |  | | |  |
| Famotidine 40mg tablets | | | | | | | | |
| System Organ Class | | **Adverse Reaction and Frequency** | | **Clinical risk** | | **Symptoms to report** | **Management** | |
| Blood and lymphatic systems | Very rare: Thrombocytopenia, Leukopenia, Agranulocytosis, pancytopenia, neutropenia | | Medically serious | | Fatigue, pallor, weakness, postural hypotension, easy bruising, recurrent infections | | | Stop drugs, needs urgent blood tests (FBC, clotting), urgent/same day review by haematologist if abnormalities detected |
| Psychiatric Disorders | Very rare: Reversible psychic disturbances including Hallucinations, Disorientation, Confusion, Anxiety disorders, Agitation, Reduced libido, Insomnia | | Medically serious | | As per side effect list | | | Exclude alternative causes, consider stopping drug, GP to manage symptoms with support of a psychiatrist if required |
| Nervous systems | Common: Headache, Dizziness | | Minor (headache, dizziness, taste disorder) Medically serious (Epileptic seizures/convulsions) | | Headache, dizziness, abnormal taste. Seizure or convulsion, absence attack, associated with memory loss, incontinence, and possible injury | | | Minor symptoms nil action required, stop drugs if intolerable. If seizure suspected, stop drugs, refer to ED if acute evidence of seizure, refer to neurology if seizures suspected but not witnessed |
|  | Uncommon: Taste disorder | |  | |  | | |  |
|  | Very rare: Paraesthesia, Somnolence, Epileptic seizures, convulsions; grand mal seizures (particularly in patients with impaired renal function); | |  | |  | | |  |
| Gastrointestinal systems | Common: Constipation, Diarrhoea | | Minor | | As per side effect list | | | GP to manage symptomatically with OTC remedies/diet changes. Stop drugs if intolerable |
|  | Uncommon: Nausea, Vomiting, Abdominal discomfort or distension, Flatulence, dry mouth | |  | |  | | |  |
| Hepato-biliary disorders | Rare: Intrahepatic cholestasis (visible sign: jaundice), | | Medically serious | | Visible jaundice in skin or eyes, non-specific, commonly detected on blood tests done for alternative reason | | | Stop drugs, check liver function, clinical review by GP/SDEC/hepatology if ALT/bilirubin >5x upper limit of normal OR visibly jaundiced OR symptomatic OR patient at risk of liver disease e.g. increased alcohol consumption |
|  | Very rare: Hepatitis; cholestatic jaundice, Increase in liver enzyme abnormalities (transaminases, gamma GT, alkaline phosphatase, bilirubin) | |  | |  | | |  |
| Metabolism and nutrition | Uncommon: Loss of appetite (anorexia) | | Minor | | Loss of appetite | | | GP to manage symptomatically with OTC remedies/diet changes. Stop drugs if intolerable |
| Skin and subcutaneous tissue disorders | Uncommon: Rash, Pruritus, Urticaria | | Minor (Pruritus/rash/urticaria). Medically serious (SJS/TEN) | | Itch, rashes, bruising. SJS/TEN has a specific rash that resembles a target with a red ring. | | | Stop drugs (all), for suspected SJS/serious rash requires same day referral to dermatology (via SDEC/ED) |
|  | Very rare: Alopecia, Stevens Johnson syndrome/toxic epidermal necrolysis sometimes fatal | |  | |  | | |  |
| Immune System disorders | Very rare: Hypersensitivity reactions (angioneurotic oedema, anaphylaxis, bronchospasm) | | Medically serious | | itch, swelling, shortness of breath, rash | | | stop drugs, dial 999 in case of anaphylactic shock reaction & follow standard anaphylactic protocols. Refer to ED if evidence of oedema |
| Respiratory, thoracic, and mediastinal disorders | Very rare: Interstitial pneumonia sometimes fatal | | Medically serious | | cough, chest pain, haemoptysis | | | Manage as per pneumonia. Stop drugs if suspected, refer to ED/SDEC if patient requires oxygen or parenteral treatment for pneumonia |
| General disorders and administration site conditions: | Uncommon: fatigue | | Minor | | As per side effect list | | | For fatigue: symptomatic management, stop drugs if intolerable and alternative causes excluded. |
|  | Very rare: chest tightness | |  | |  | | | For chest tightness: exclude alternative causes, consider stopping drug, may need ECG |
| Reproductive system and breast disorders: | Very rare: impotence, rare cases of gynecomastia, have been reported, however, in controlled clinical trials the incidences were not greater than those seen with placebo. | | Minor | | impotence, increased breast tissue in males | | | symptomatic management, stop drugs if intolerable and alternative causes excluded |
| Musculoskeletal, connective tissue and bone disorders | Very rare: Muscle cramps, Arthralgia | | Minor | | cramping, pain in muscles or joints | | | symptomatic management, stop drugs if intolerable |

| Colchicine 500mcg tablets | | | | | |
| --- | --- | --- | --- | --- | --- |
| System Organ Class | **Adverse Reaction and *Frequency*** | **Clinical risk** | | **Symptoms to report** | **Management** |
| Gastrointestinal system disorders | *Common:* Abdominal pain, nausea, vomiting and diarrhoea.  *Not known:* Gastrointestinal Bleeding | GI bleeding medically serious | | Vomiting blood/coffee grounds, dark stools, Fatigue, pallor, postural dizziness, fresh blood PR | Cessation of drug, same-day hospital assessment (SDEC), likely to need endoscopy +/- colonoscopy & prescription of Drugs to stop bleeding (PPI) |
| Skin and subcutaneous tissue disorders | *Not known:* Alopecia and rash | Minor | | Rash | Stop drugs, dermatology referral if persists |
| Renal and urinary disorders | *Not known:* Renal damage | Medically serious | | Non-specific, commonly detected on blood tests done for alternative reason | Stop drugs, check renal function, clinical review by GP/SDEC |
| Hepatobiliary disorders | *Not known:* Hepatotoxicity | Medically serious | | Non-specific, commonly detected on blood tests done for alternative reason | Stop drugs, check liver function, clinical review by GP/SDEC |
| (Female) Reproductive system disorders | *Not known:* Menstrual cycle irregularities; amenorrhoea, dysmenorrhoea | Minor | | Heavy periods | Stop drugs, check FBC, may need iron replacement if anaemic |
| Musculoskeletal system | *Not known:* Myopathy and rhabdomyolysis | Medically serious | | Muscle pain & weakness | Stop drugs, check creatine kinase & renal function, clinical review by GP/SDEC, may need IV fluids & careful monitoring of renal function |
| Nervous System | *Not known:* Peripheral neuritis, neuropathy. | Important | | Nerve pain or weakness | Stop drugs, clinical review by GP/SDEC, may need neurology referral |
| (Male) Reproductive system disorders | *Not known:* oligospermia, azoospermia. | Important | | Primary infertility | Stop drugs, clinical review by andrologist |
| Blood and lymphatic system disorders | *Not known:* bone marrow depression with agranulocytosis, aplastic anaemia, and thrombocytopenia. | Medically serious | | Fatigue, pallor, weakness, postural hypotension, easy bruising, recurrent infections | Stop drugs, needs urgent blood tests (FBC, clotting), urgent/same day review by haematologist if abnormalities detected |
| Rivaroxaban 10mg tablets | | | | | |
| System Organ Class | **Adverse Reaction and *Frequency*** | | **Clinical risk** | **Symptoms to report** | **Management** |
| Blood and lymphatic system disorders | *Common:* Anaemia (incl. respective laboratory parameters)  *Uncommon:* Thrombocytosis (incl. platelet count increased), thrombocytopenia | | Minor | Fatigue, pallor, weakness, postural hypotension, easy bruising | Stop drugs, check FBC, may need iron replacement if anaemic |
| Immune system disorders | *Uncommon:* Allergic reaction, dermatitis allergic, angioedema and allergic oedema  *Very rare:* Anaphylactic reactions including anaphylactic shock | | Medically serious | Itch, swelling, shortness of breath, rash | Stop drugs, dial 999 in case of anaphylactic shock reaction & follow standard anaphylactic protocols. Refer to ED if evidence of oedema |
| Nervous system disorders | *Common:* Dizziness, headache  *Uncommon:* Cerebral and intracranial haemorrhage, syncope | | Medically serious (intracranial haemorrhage) | severe headache, weakness of limbs, loss of speech or rapid loss of consciousness | Dial 999 report suspected stroke. Requires urgent assessment in a stroke centre |
| Eye disorders | *Common:* Eye haemorrhage (incl. conjunctival haemorrhage) | | Medically serious | visible blood in the eye, sudden loss of vision, eye pain (unilateral) | stop drugs, refer to same day ophthalmology services |
| Cardiovascular disorders | *Common:* Hypotension, haematoma  *Uncommon:* Tachycardia | | Minor | dizziness, palpitations, bruising | stop drugs, GP review, check ECG, FBC & blood pressure |
| Respiratory, thoracic, and mediastinal disorders | *Common:* Epistaxis, haemoptysis | | Minor | visible blood either on coughing or from the nose - difficult or slow to stop with compression | stop drugs, refer to ED if bleeding not stopping for ENT review |
| Gastrointestinal disorders | *Common:* Gingival bleeding, gastrointestinal tract haemorrhage (incl. rectal haemorrhage), gastrointestinal and abdominal pains, dyspepsia, nausea, constipation, diarrhoea, vomiting  *Uncommon:* Dry mouth | | GI bleeding medically serous | Vomiting blood/coffee grounds, dark stools, fatigue, pallor, postural dizziness, fresh blood PR | cessation of drug, same-day hospital assessment (SDEC), likely to need endoscopy +/- colonoscopy & prescription of drugs to stop bleeding (PPI) |
| Hepatobiliary disorders | *Common:* Increase in transaminases  *Uncommon:* Hepatic impairment, increased bilirubin, increased blood alkaline phosphatase, increased GGT  *Rare:* Jaundice, bilirubin conjugated increased (with or without concomitant increase of ALT), cholestasis, hepatitis (incl. hepatocellular injury) | | Medically serious | non-specific, commonly detected on blood tests done for alternative reason | stop drugs, check liver function, clinical review by GP/SDEC/hepatology if ALT/bilirubin >5x upper limit of normal OR visibly jaundiced OR symptomatic OR patient at risk of liver disease e.g. increased alcohol consumption |
| Skin disorders | *Common:* Pruritus (incl. uncommon cases of generalised pruritus), rash, ecchymosis, cutaneous and subcutaneous haemorrhage  *Uncommon:* Urticaria  *Very rare:* Stevens-Johnson syndrome/Toxic Epidermal Necrolysis, DRESS syndrome | | Minor (Priuritis) Medically serious (SJS/TEN) | Itch, rashes, bruising. SJS/TEN has a specific rash that resembles a target with a red ring. | stop drugs (all), for suspected SJS/serious rash requires same day referral to dermatology (via SDEC/ED) |
| Musculoskeletal and connective tissue disorders | *Common:* Pain in extremity  *Uncommon:* Haemarthrosis  *Rare:* Muscle haemorrhage  *Not known:* Compartment syndrome secondary to a bleeding | | Medically serious | Pain in joint or leg, disproportionate pain to visible changes in limb | Stop drugs, analgesia, GP review. Requires urgent referral to SDEC/ED if haemarthrosis/haemorrhage/comparetment syndrome suspected by GP |
| Renal and urinary disorders | *Common:* Urogenital tract haemorrhage (incl. haematuria and menorrhagia), renal impairment (incl. blood creatinine increased, blood urea increased)  *Not known:* Renal failure/acute renal failure secondary to a bleeding sufficient to cause hypoperfusion | | Minor (urogenital tract haemorrhage). Medically serious (renal failure/AKI) | Visible blood in urine, heavy periods, fatigue, weakness, muscle pain, itch. | Stop drugs, check renal function, clinical review by GP/SDEC if evidence of AKI. May need renal ultrasound and review by nephrologist |
| General disorders and administration site conditions | *Common:* Fever, peripheral oedema, decreased general strength and energy (incl. fatigue and asthenia)  *Uncommon:* Feeling unwell (incl. malaise)  *Rare:* Localised oedema | | Minor | Fatigue, fever, weakness, malaise | Exclude bleeding via medical history and FBC. Exclude other causes of fever and malaise. Stop drug |
| Investigations | *Uncommon:* Increased LDH, increased lipase, increased amylase | | Medically serious | Fatigue, fever, weakness, malaise, abdominal pain | Stop drugs, if symptomatic refer to ED/SDEC. If asymptomatic GP to exclude alternative causes of abnormal enzymes including alcohol and gallstones via history and upper abdominal ultrasound. Monitor enzymes every 48 hours, if increasing refer to ED/SDEC |
| Injury, poisoning and procedural complications | *Common:* Postprocedural haemorrhage (incl. postoperative anaemia, and wound haemorrhage), contusion, wound secretion  *Rare:* Vascular pseudoaneurysm | | Medically important | pain, weakness, bleeding, oozing from wound | stop drugs, check FBC, may need iron replacement if anaemic. If pseudoaneurysm suspected requires imaging of the appropriate area and input of a vascular surgeon. |

# Appendix II

**Definition of Acceptable Effective Contraception for this Trial adapted from the Recommendations related to contraception and pregnancy testing in clinical trials** (42)

• combined (estrogen and progestogen containing) hormonal contraception associated with inhibition of ovulation ^1^:

o oral

o intravaginal

o transdermal

• progestogen-only hormonal contraception associated with inhibition of ovulation ^1^:

o oral o injectable

o implantable ^2^

• intrauterine device (IUD) ^2^

• intrauterine hormone-releasing system ( IUS) ^2^

• bilateral tubal occlusion ^2^

• vasectomised partner ^2,3^

• sexual abstinence ^4^

- progestogen-only oral hormonal contraception, where inhibition of ovulation is not the primary mode of action

- male or female condom with spermicide ^a^

- cap, diaphragm or sponge with spermicide^a^

^a^A combination of male condom with either cap, diaphragm or sponge with spermicide (double barrier methods) are also considered acceptable birth control methods

^1^ Hormonal contraception may be susceptible to interaction with the IMP, which may reduce the efficacy of the contraception method.

^2^ Contraception methods that in the context of this guidance are considered to have low user dependency.

^3^ Vasectomised partner is a highly effective birth control method provided that partner is the sole sexual partner of the WOCBP trial participant and that the vasectomised partner has received medical assessment of the surgical success.
