# Supplementary material for: STIMULATE-ICP: A pragmatic, multi-centre, cluster randomised trial of an integrated care pathway with a nested, Phase III, open label, adaptive platform randomised drug trial in individuals with Long COVID: A structured protocol
Source: PLoS One. 2023 Feb 15;18(2):e0272472. doi: 10.1371/journal.pone.0272472 (PMC9931100; doi:10.1371/journal.pone.0272472)
Supplement: S1 Appendix — (DOCX) [file pone.0272472.s002.docx]

***Appendix 1:***

***Rationale for initial drugs in drug platform***

### Loratadine and Famotidine (H_1_ + H_2_ Receptor Blockade)

Loratadine and Famotidine are both histamine receptor antagonists. In combination they inhibit both the H_1_ and H_2_ receptors. Famotidine is an effective competitive H_2_ receptor antagonist. It reduces the concentration and amount of acid and pepsin of the gastric juices. The effect of oral administration is rapid, long lasting when used at the recommended dosage and it is effective with relatively low concentration in the blood. The duration of its effect, plasma concentration and secretion in the urine are dose-dependent. Famotidine is licensed as an over the counter (OTC) treatment for dyspepsia/gastric ulceration. Loratadine is also an OTC treatment licensed for treatment of mild allergic symptoms including seasonal rhinitis.

Individuals with Long COVID (LC) are hypothesised to have a persistent inflammatory process that may drive the symptoms of fatigue and myalgia. In a small minority of individuals, more specific symptoms suggestive of mast-cell activation, including rashes, diarrhoea and flushing may be present. Histamine receptor antagonists are commonly used to treat mast-cell activation in other conditions.

Mixed reports of efficacy of histamine receptor antagonists in acute COVID-19, particularly reduction in oxygen requirements and possibly mortality, have led to ongoing clinical trials of these treatments in acute COVID-19 (27). The suggested mechanisms of action in acute COVID-19 are reduction in inflammation due to suppression of T-cell mediated cytokine release, however, available data are very limited. In an observational cohort study, 49 patients with LC were compared to matched asymptomatic fully recovered COVID-19 individuals without LC. Individuals with LC were offered treatment with combined histamine H1/H2 blockade, using H1 (Loratadine 10 mg once daily or Fexofenadine 180 mg twice daily (not being used in this study) and H_2_ (Famotidine 40 mg once daily or Nizatidine 300 mg once daily (not being used in this study)) for a minimum of 4 weeks (28), based on data from acute COVID-19 suggesting histamine receptor antagonist therapy improved symptoms (29, 30). In this preliminary observational study, individuals with physician-diagnosed LC reported a 48% reduction in symptom burden after 4 weeks of combined histamine receptor antagonist therapy, compared to baseline. When compared to recovered non-hospitalised individuals, individuals with LC had significantly lower circulating CD4 but not CD8 effector memory cells, suggesting that this T cell subset may be involved in LC pathology (28). These data, however, have significant limitations, and the findings require testing in a formal clinical trial.

In this study we will be using famotidine 40mg once daily in combination with loratadine 10mg OD, ensuring combined histamine receptor antagonist activity across the H_1_ and H_2_ receptors. Both drugs are being used within their licensed dose and are safe to be co-administered.

### Colchicine

Colchicine inhibits cellular transport and mitosis by binding to tubulin and preventing its polymerisation as part of the cytoskeleton transport system. Colchicine has a short half-life of 9-12 hours and is prescribed as a BD (twice daily) dosing regimen. Standard doses for acute gout range from 500mcg BD to 2mg BD, depending on the dose response. Colchicine has a wide range of anti-inflammatory effects, including inhibition of certain inflammasomes (cytosolic pattern recognition receptor systems that are activated in response to detection of pathogens in the cytosol) (31), (32). Evidence shows that inflammasomes are activated in COVID-19, and the degree of activation is correlated with disease severity ( (33)).

Colchicine has been shown to have cardiovascular benefit in individuals with coronary artery disease and pericarditis at a dose of 500mcg BD. Its primary mechanism of action is reduction of serositis, inflammation of membranes around joints and viscera. Individuals with LC frequently complain of symptoms suggestive of serositis, either atypical chest pain that may indicate pericarditis, costochondritis or pleural inflammation, or joint pain in the absence of clinically evident inflammation. Disease severity in LC correlates with myocardial damage on Coverscan™ (15). Of the non-hospitalised individuals with LC at UCLH, 20% report chest pain and or palpitations, 60% report shortness of breath. Standard investigations are frequently normal: prolonged rhythm monitoring typically shows resting sinus tachycardia, chest pain is generally atypical with predominantly normal echocardiogram, Troponin T and ECG. In a prospective cohort of over 50 individuals (median age 43, 69% female) reporting persistent chest pain underwent cardiac MRI (CMR). 26% had evidence of myocarditis-pattern late gadolinium enhancement and/or evidence of abnormalities, (meeting diagnostic criteria for myocarditis (34). T1/T2 abnormalities were seen in 29%. Individuals with LC with myocarditis contrasted to individuals admitted to hospital with moderate/severe respiratory COVID (mean age 64, 66% male). However, the prevalence of myocarditis is strikingly similar (35). To date, we have treated in excess of 200 LC individuals with abnormal CMR empirically with colchicine 500mcg BD, 9/10 reported significant symptomatic improvement within 3 months.

### Rivaroxaban (Low Dose Anticoagulation)

Rivaroxaban is an oral factor Xa inhibitor, inhibiting the clotting cascade. Individuals with LC who complain of marked exertional fatigue with abnormalities on the 6-minute walk test, have evidence of microvascular anaerobic respiration, despite normal oxygenation in peripheral blood. This suggests that while adequate haemoglobin-bound oxygen is present in blood, the dissociated oxygen is unavailable to large muscles on increased aerobic demand during exercise. Abnormalities of the clotting cascade are a marked feature of acute Covid-19, including microvascular thrombi (36). Thrombi in acute Covid-19 are strongly associated with abnormally elevated von-Willebrand Factor (VWF): ag/ADAMTS 13 ratio (37). Recent data are showing longer term risk of VTE with LC. We extended the measurement of VWF Ag/ADAMTS 13 levels to the UCLH community LC cohort (Scully and Heightman, 2021. Unpublished data.) Of 272 patients in LC clinic describing extreme lethargy, headaches and poor exercise tolerance, 81/272 (30%) had an abnormal VWF Ag/ADAMTS 13 ratio of >1.5. Elevated VWF Ag/ADAMTS 13 ratio strongly associated with impaired exercise capacity on a 6-minute walk test: 1.5 compared to 1.1 in patients with normal exercise capacity (p<0.001).

A further 28 patients had blood analysed by flow chamber assay, which measures VWF, platelet binding and in-vitro thrombus formation in real time. 8/28 (29%) had clot formation by 5 minutes. In a small feasibility project, 5 patients were initiated on low dose aspirin (LDA) 75mg daily, selected based on symptom severity and elevated VWF(Ag):ADAMTS13 ratio. In 3/5 patients, improvement in symptoms and VWF(Ag):ADAMTS13 ratio was observed. The remaining two stopped aspirin due to upcoming procedure and bruising. Patients B and C were analysed on the flow chamber pre- and post-aspirin usage with marked improvement in surface coverage from 100% to 29% and 9% respectively on LDA, reporting corresponding symptom improvement.

Evidence of microvascular thrombi in some patients has led to the hypothesis that that endothelial dysfunction due to multiple microvascular thrombi in large muscles may significantly contribute to reduced aerobic capacity and symptoms of fatigue. Low-dose anticoagulation is a safe approach to test this hypothesis, by measuring fatigue as the primary outcome, we will determine if this approach indicates the presence of microvascular thrombi in LC patients. Aspirin was poorly tolerated when tested in our pilot study due to gastro-intestinal side effects. We have included rivaroxaban 10mg once daily in the STIMULATE-ICP trial instead of aspirin due to improved safety profile, easy comparability with other clinical trials using factor Xa inhibitors such as apixaban (HEAL-COVID) and proven efficacy data on prophylaxis of intravascular thrombi. The rivaroxaban regime of 10mg OD proposed in STIMULATE-ICP is a prophylaxis dose, which aims to effectively prevent generation of further microvascular thrombi, while minimising bleeding side effects. This prophylaxis dose is therapeutically equivalent to low-dose aspirin tested in our preliminary cohort.
