# Supplementary material for: STIMULATE-ICP: A pragmatic, multi-centre, cluster randomised trial of an integrated care pathway with a nested, Phase III, open label, adaptive platform randomised drug trial in individuals with Long COVID: A structured protocol
Source: PLoS One. 2023 Feb 15;18(2):e0272472. doi: 10.1371/journal.pone.0272472 (PMC9931100; doi:10.1371/journal.pone.0272472)
Supplement: S2 Appendix — (DOCX) [file pone.0272472.s003.docx]

***Appendix 2:***

**Additional Inclusion Criteria for the nested, platform randomised drug trial**

*N.B: Potential participants with drug-specific contraindications for any arm, including interactions of pre-prescribed essential medication will be consented for data collection but will be excluded from the drug study.*

1. Females of childbearing potential (see definition below) must be willing to use at least an acceptable effective method of contraception during the treatment with investigational medical product (IMP) and for a further 30 days after the last dose. (34).

Such methods include:

a. combined (oestrogen and progestogen containing) hormonal contraception:

- 1. oral
  2. intravaginal
  3. transdermal

b. progestogen-only hormonal contraception

1. oral
2. injectable
3. implantable

c. intrauterine device (IUD)

d. intrauterine hormone-releasing system (IUS)

e. bilateral tubal occlusion

f. vasectomised partner

g. male or female condom with spermicide

h. cap, diaphragm or sponge with spermicide

i. sexual abstinence; only true abstinence is acceptable i.e. when this is in line with the preferred and usual lifestyle of the participant). (Periodic abstinence, declaration of abstinence during exposure to IMP and withdrawal are not accepted methods of contraception).

Definition of females of childbearing potential:

For the purpose of this trial, a female is considered of childbearing potential i.e. fertile following menarche and until becoming post-menopausal unless permanently sterile. Permanent sterilisation methods include hysterectomy, bilateral salpingectomy, and bilateral oophorectomy.

A post-menopausal state is defined as no menses for 12 months without alternative medical cause.

2. Male Participants must be willing to use condom during IMP treatment to protect their female partner becoming pregnant and for a further 90 days after the last dose.

3. Patients on pre-existing treatments for the same drug classes MUST undergo a 7-day washout period before being randomised.

*(Patients will be assessed, and if safe to do so, exclude that medication for 7 days, asked if they would be willing to undergo a washout period of at least 7 days before being randomised.)*

*Exclusion Criteria for ALL Participants*

1. Previously hospitalised for COVID-19 infection.

2. Previously referred to a LC clinic.

*Exclusion criteria for nested, adaptive randomised drug trial*

3. Females who are pregnant, planning pregnancy or breastfeeding

4. Known hypersensitivity to any of the study drugs or their excipients

5. Currently taking any of the following drugs:

Probenecid, Sucrafate, Isocarboxazid, Phenylzine, Tranylcypromine of any other CNS depressant (such as diphenhydramine, dextromethorphan, or pseudoephedrine) *(Contraindications to famotidine/loratadine)*

Amiodarone, Aprepitant, Atanazavir, Atorvostatin, Azithromycin, Bezafibrate, Ciclosporin, Ciprofibrate, Clarithromycin, Cobicistat, Croztibib, Darunavir, Diltiazem, Dronedarone, Eliglustat, Erythromycin, Fenobibrate, Fluconazole, Fluvastatin, Fosamprenavir, Gemfibrozil, Idelalisib, Imatibib, Isavuconazole, Itraconazole, Ketoconazole, Letermovir, Lopinavir, Netupitant, Nilotinib, Posaconazole, Pravastatin, Ranolazine, Ritonavir, Rosuvastatin, Simvastatin, Tipranavir, Velpatasvir, Vemurafenib, Venetoclax, Verapamil, Voriconazole *(Contraindications to colchicine)*

Acalabrutinib, Aceclofenac, Acenocoumarol, Alprostadil, Alteplase, Argatroban, Aspirin, Axitinib, Beniparin, Benzydamine, Bevacizumab, Bismuth, Bivalirudin, Bosutinib, Bromfenac, Cabozantinib, Cangrelor, Caplacizumab, Celecoxib, Cilostazol, Clopidogrel, Cobimetinib, Dabigatran, Dalteparin, Danaparoid, Dasatinib, Dexkeptorofen, Diclofenac, Dipyridamole, Enoxaparin, Epoprostenol, Eptifibatide, Etodolac, Etoricoxib, Flurbiprofen, Heparin, Ibrutanib, Ibuprofen, Iloprost, Imatinib, Indomethacin, Inotersen, Ketoprofen, Ketorolac, Levatinib, Mefenamic acid, Meloxicam, Nabumetone, Naproxen, Nicotinic acid, Nintenanib, Parecoxib, Pazopanib, Phenazone, Phenindione, Piroxicam, Ponatinib, Prasugrel, Regorafenib, Ruxolitinib, Sorafenib, Streptokinase, Sulindac, Sunitinib, Tenecteplase, Tenoxicam, Tiaprofenic acid, Ticagrelor, Tinzaparin, Tirofiban, Tolfenamic acid, Trametinib, Traztuzumab emtansine, Trprostinil, Urokinase, Volanesorsen, Warfarin *(Contraindications to Rivaroxaban)*

6. Renal failure/insufficiency (eGFR<30ml/minute) on the basis of blood investigations (eGFR) within the last 6 months and clinical assessment

7. Severe liver dysfunction on the basis of blood investigations within the last 6 months (liver function and coagulation) and clinical assessment

**Assessment of eligibility, recruitment and withdrawal of consent**

Site-specific research staff are embedded within LC clinics and will identify potential participants from new clinic referrals. All new referrals will be sent the trial information sheet and research team contact details prior to attendance at their first appointment, allowing time to consider taking part prior to their appointment. In instances where the potential participant has not received information, then information will be provided at time of approach or clinic visit. The individual will be given as much time as necessary to decide regarding participation and may consent to the drug trial on that day, if they wish, or return within 7-10 days post-clinic appointment. Only consent for data collection and blood collection can be taken prior to clinic. Some individuals interested in participating in the trial may have “brain fog” and, therefore, may require more time to consider taking part.

Randomisation into the drug study can only occur after confirmation of eligibility by clinic and trial teams. Potential participants will also be given study information by their treating physician at the LC clinic appointment. Drug trial eligibility must be confirmed by the principal investigator or suitably delegated person as per delegation log, following results of clinical assessments, to confirm the participant meets all inclusion criteria and none of the exclusion criteria. Individuals who are ineligible or opt not to participate in the drug trial will be asked to consent for data collection or data collection plus blood samples for investigation in diagnostic and pathogenesis sub-studies.

The following must be available to confirm eligibility for the nested drug trial:

- Participant full medical history (at LC clinic visit)
- Concomitant medication review (at LC clinic visit)
- Physical Examination (at LC clinic visit)
- Urine pregnancy test within 7 days prior to the first dose of drug (in women of childbearing potential)
- Estimated glomerular filtration rate (eGFR) (within last 6 months)
- Liver function test (within last 6 months)
- Full blood count (FBC) (within last 6 months)
- Coagulation screening (within last 6 months)

Site-specific research practitioners embedded in LC Clinics will identify new referrals and, if from within a primary care network with Coverscan™ as usual care, will arrange a scan appointment 4 weeks prior to first LC clinic appointment (usual care within this primary care network, following the same pathway as other clinical appointments). Similarly, individuals referred from primary care networks allocated to “Living with COVID recovery™” app will be identified and referred to the physiotherapist/s assigned to delivery of this app, who will contact participants directly (as usual care). An appointment with the participant may be required prior to LC clinic to discuss the App, wherever possible or within 7 -10 days of the first LC clinic appointment. For individuals where English may not be their first language, the information will contain details of how they can take part assisted by an English-speaking relative/friend.

Participants are free to withdraw at any time from the trial without giving reasons and without prejudicing further treatment and must be provided with a contact point where further information about the trial may be obtained. Data and samples collected up to the point of withdrawal will be used with consent after withdrawal. Any intention to utilise such data shall be included and outlined in the consent form. It will be made clear that, if data has been anonymised and aggregated, it will not be possible to identify individual data for withdrawal. It will also not be possible to identify any one person from such data.

Participants will be notified that their identifiable data (Name, date of birth, NHS Number, and contact details) contact be shared with Lancashire Clinical Trials Unit to enable contact for follow-up within the study, and for well-being reporting if needed. This trial will only recruit participants who are able to consent. If a participant later becomes incapacitated, the participant shall be withdrawn from the trial and no further data will be collected or any trial investigation or assessments conducted. It is not expected that this would be disease progression within this group. Assessments relating to safety issues arising from participation will continue until the trial ends but will be the remit of the patient`s clinician.

Participants allocated to the cluster with the Living with COVID Recovery™ digital app will be informed that data related to their symptoms, physical and mental health, heart rate and physical activities, through the use of the app, will be used to inform their care plan by the Multidisciplinary Team at the LC clinics. The App forms part of their usual care and is part of the clinical record: data from consented individuals will be used by the trial team to assess use of the app (level of engagement) and use of content-specific areas within the app.
