# Supplementary material for: STIMULATE-ICP: A pragmatic, multi-centre, cluster randomised trial of an integrated care pathway with a nested, Phase III, open label, adaptive platform randomised drug trial in individuals with Long COVID: A structured protocol
Source: PLoS One. 2023 Feb 15;18(2):e0272472. doi: 10.1371/journal.pone.0272472 (PMC9931100; doi:10.1371/journal.pone.0272472)
Supplement: S3 Appendix — (DOCX) [file pone.0272472.s004.docx]

***Appendix 3:***

***Samples for Future Research and Biobanking Storage***

Approximately 5mL of the research blood taken will be for biobanking at Perspectum and use in future ethically approved research studies research regarding the pathophysiology and mechanism of long COVID, where separate ethical approval may be required. This blood will be stored in an HTA registered biobank at Perspectum’s central laboratory and UCL will remain the custodian.

***Samples for Sub-Study Analysis at Central Laboratories***

Approximately 60 mls of the blood sample will be used for the analyses set out below and relate to the secondary endpoint of the trial. The frozen components of the blood samples will be stored at Perspectum’s central laboratory and sent on to sub-contracted laboratories for testing. In the case of UCLH patient samples for Functional T-cell and live-virus neutralisation antibodies assay, they will be sent direct from the UCLH site to the Francis Crick Institute for analysis.

Guided by clinical practice, patient lived experience and latest scientific hypotheses, the following analyses will be performed in blood samples of some participants, extending to the whole cohort only if there is a clear scientific rationale. Any samples not used within these sub-studies will be retained within the biobank at Perspectum for future research use. All samples will be sent to Perspectum and stored until analysis by third party laboratories:

I. Genomics analysis: one sample per patient will be taken for initial genome-wide and focused gene (using a long list of immune-regulated genes) analyses. They will be performed using standard protocols adjusting for any population structure. Models will incorporate clinical and environmental determinants of disease severity.

II. Proteomics analysis one sample per patient. Proteomics will be assessed by proximity extension assay enabling over 1400 proteins to be rapidly analysed. The assay uses oligonucleotide-labelled antibody pairs allowing for pair-wise binding to target proteins.

III. Metabolomics and Lipidomics; one sample per patient : A combination of Liquid Chromatography with tandem mass spectrometry (LC-MS/MS) based metabolomics and lipidomics will be performed based on a targeted analysis of over 200 metabolites of core metabolism, including acyl-carnitines, acyl-CoAs, amino acids, glycolysis and TCA intermediates and nucleotides using a Thermo Quantiva triple quadrupole mass spectrometer and lipidomics by open-profiling UHPLC-MS/MS using a Thermo Elite Orbitrap interfaced with an Advion Nanomate to allow direct nanoinfusion to detect over 600 annotated lipids.

IV. Functional T-cell and live-virus neutralisation antibodies in participants recruited to the London trial site. Two samples per patient will be collected (approximately 30mL). Serum and peripheral blood mono-nuclear cells (PBMCs) will be isolated on arrival at the FCI. Cells will be stained and analysed using mass-cytometry and neutralising antibodies quantified in the live-virus neutralisation assay.

V. Endocrine investigation (thyroid, hypothalamo-pituitary- gonadal and hypothalamo-pituitary- adrenal axes) will be included for those with suggestive symptoms associated with thyroiditis, autoimmune hypothyroidism and adrenal impairment for detailed phenotyping to define potential pathophysiology involved in ongoing organ-specific or physiological abnormalities for example endocrine disturbances explaining diverse nonspecific symptoms, including fatigue, hypothermia and dysmenorrhoea.
