# Supplementary material for: STIMULATE-ICP: A pragmatic, multi-centre, cluster randomised trial of an integrated care pathway with a nested, Phase III, open label, adaptive platform randomised drug trial in individuals with Long COVID: A structured protocol
Source: PLoS One. 2023 Feb 15;18(2):e0272472. doi: 10.1371/journal.pone.0272472 (PMC9931100; doi:10.1371/journal.pone.0272472)
Supplement: S4 Appendix — (DOCX) [file pone.0272472.s005.docx]

***Appendix 4:***

**Functional tests in some Long Covid clinics**

1. Chest X Ray
2. High Resolution Computerised Tomography (CT) scan of the Chest
3. CT Pulmonary Angiogram
4. Pulmonary Function Test
5. 6-minute walk test
6. 1-minute sit to stand test
7. Functional Exhaled Nitric Oxide (FeNO) test
8. Echocardiogram (ECHO)
9. Electrocardiogram (ECG) if the patient had cardiac symptoms
10. Holter monitor of the heart
11. Cardiovascular Magnetic Resonance Scan (CMR)
12. Stress Electrocardiogram
13. Magnetic Resonance Imaging (MRI) scan of Brain
14. Tilt Table Test
15. Coverscan™
