# Supplementary material for: STIMULATE-ICP: A pragmatic, multi-centre, cluster randomised trial of an integrated care pathway with a nested, Phase III, open label, adaptive platform randomised drug trial in individuals with Long COVID: A structured protocol
Source: PLoS One. 2023 Feb 15;18(2):e0272472. doi: 10.1371/journal.pone.0272472 (PMC9931100; doi:10.1371/journal.pone.0272472)
Supplement: S5 Appendix — (DOCX) [file pone.0272472.s006.docx]

***Appendix 5:***

**Functional and Patient Reported Outcome Assessments at Baseline**

All participants must have given consent before the following Patient Reported Outcome Questionnaires and functional tests are carried out:

1. Fatigue Assessment Score(FAS)
2. EuroQol Research Foundation Health related quality of life (EQ-5D-5L)
3. Mental health (GAD-7)
4. Medical Research Council Dyspnoea Score
5. Public Health Questionnaire - Depression (PHQ-9)
6. Perceived Deficit Questionnaire (PDQ-5)
7. Work and Social Adjustment Scale (WSAS) [ Question 4 from Productivity Cost Questionnaire (iPCQ) for absenteeism and Question 8 from iPCQ for presenteeism added]
8. Short Form Questionnaire (SF-12)
9. Cognitive Failure Questionnaire (CFQ) if a patient scores 3 or more on PDQ5 (patients receive an email to complete this questionnaire online via a secure password and patient ID number)
10. Functional abilities and physical function using pedometer monitoring/wearables data
11. Organ impairment and healthcare utilisation
12. Cost-effectiveness of ICP
13. Process outcomes for different ICP components
