# Supplementary material for: STIMULATE-ICP: A pragmatic, multi-centre, cluster randomised trial of an integrated care pathway with a nested, Phase III, open label, adaptive platform randomised drug trial in individuals with Long COVID: A structured protocol
Source: PLoS One. 2023 Feb 15;18(2):e0272472. doi: 10.1371/journal.pone.0272472 (PMC9931100; doi:10.1371/journal.pone.0272472)
Supplement: S6 Appendix — (DOCX) [file pone.0272472.s007.docx]

***Appendix 6:***

**12-Week Assessment Visit**

1. Fatigue Assessment Score
2. IMP accountability (over the phone or in person at the clinic)
3. 6-minute walk test (if performed at baseline visit and where possible undertaken at follow-up)
4. 1-minute Sit to Stand test (if performed at baseline visit and where possible undertaken at follow-up)
5. Medical Research Council (MRC) dyspnoea score
6. Modified Work and Social Adjustment Scale (WSAS) [Q4 from iPCQ for absenteeism and Q8 from iPCQ for presenteeism added]
7. General Anxiety Disorder Questionnaire- 7 (GAD-7)
8. The Primary Care Evaluation of Mental Disorders Patient Health Questionnaire (PHQ-9)
9. EQ-5D
10. Perceived Deficit Questionnaire (PDQ-5)
11. 12-item Short Form Survey (SF12)
12. Cognitive Failure Questionnaire (CFQ) if a patient scores 3 or more on PDQ5 (patients receive an email to complete this questionnaire online via a secure password and patient ID number)
13. Adverse Event review (over the phone or in person in clinic) – Patient completed eCRFs or paper questionnaires will be reported back to site PIs by Lancashire CTU, for review and follow-up of any potential AEs reported by patients
14. Concomitant medication review (over the phone or in person at the clinic)
