# Supplementary material for: STIMULATE-ICP: A pragmatic, multi-centre, cluster randomised trial of an integrated care pathway with a nested, Phase III, open label, adaptive platform randomised drug trial in individuals with Long COVID: A structured protocol
Source: PLoS One. 2023 Feb 15;18(2):e0272472. doi: 10.1371/journal.pone.0272472 (PMC9931100; doi:10.1371/journal.pone.0272472)
Supplement: S7 Appendix — (DOCX) [file pone.0272472.s008.docx]

***Appendix 7:***

**24-Week Assessment Visit**

1. Fatigue Assessment Score
2. IMP accountability (over the phone or in person at the clinic)
3. 6-minute walk test (if performed at baseline visit and where possible undertaken at follow-up)
4. 1-minute Sit to Stand test (if performed at baseline visit and where possible undertaken at follow-up)
5. MRC dyspnoea score
6. Modified Work and Social Adjustment Scale (WSAS) [Q4 from iPCQ for absenteeism and Q8 from iPCQ for presenteeism added]
7. General Anxiety Disorder Questionnaire- 7 (GAD-7)
8. The Primary Care Evaluation of Mental Disorders Patient Health Questionnaire (PHQ-9)
9. EQ-5D-5L
10. Perceived Deficit Questionnaire (PDQ-5)
11. 12-item Short Form Survey (SF12)
12. Cognitive Failure Questionnaire (CFQ), if a patient scores 3 or more on PDQ5 (patients receive an email to complete this questionnaire online via a secure password and patient ID number)
13. Functional ability and Fidelity of delivery of Treatment as Usual and Living with COVID Recovery ^TM^
14. Adverse Event review (over the phone or in person in clinic) for participants on the nested drug trial and participants expressing suicidal ideation on the patient reported outcome questionnaires. Patient completed eCRFs or paper questionnaires will be reported back to site PIs by Lancashire CTU, for review and follow-up of any potential AEs reported by patients. AEs will be reported up to 28 days following the last dose of the trial drugs.
15. Concomitant medication review (over the phone or in person at the clinic) for participants on the nested drug trial only. Concomitant medications will be reported up to 28 days following the last dose of the trial drugs.
