# Supplementary material for: STIMULATE-ICP: A pragmatic, multi-centre, cluster randomised trial of an integrated care pathway with a nested, Phase III, open label, adaptive platform randomised drug trial in individuals with Long COVID: A structured protocol
Source: PLoS One. 2023 Feb 15;18(2):e0272472. doi: 10.1371/journal.pone.0272472 (PMC9931100; doi:10.1371/journal.pone.0272472)
Supplement: S8 Appendix — (DOCX) [file pone.0272472.s009.docx]

***Appendix 8:***

**Data Collection (further details)**

Individuals referred from a primary care network who are allocated to Coverscan™, will be sent an appointment for the scan 4 weeks prior to their LC appointment. The scan results will be assessed and reported by a specialist radiologist group supported by Perspectum. Reports will be returned to clinic site and become part of the clinical record, and available to clinicians at time of the clinic visit to inform clinical decision-making. Uptake of CoverscanTM will be entered into the trial database for analysis.

Routine clinical data from patients undergoing investigations at participating LC clinics will be captured through patient-facing questionnaire and research staff-completed CRF.

Participants will be consented for use of NHS data regarding medical history (e.g. hospital and outpatient attendances, prescriptions, and other relevant information) from January 2020 up to 1 year following study enrolment. Data capture will be via NHS Digital using NHS number.

The chief investigator, Sponsor and trial statisticians have access to the full pseudonymised dataset prior to database lock and data analysis at the end of the trial. Collaborators and the principal investigators have access to anonymised data extracts at the end of the study

**Data management plan**

Quality Control (QC) includes the operational techniques and activities done within the QA system to verify that the requirements for quality of the trial-related activities are fulfilled. A risk-adapted approach will be used for monitoring. All sites will be centrally monitored for recruitment, data completeness, quality and timeliness of data entry, number of data change requests. A minimum routine remote monitoring schedule of 3 monthly will be set. Any concerns not resolved or raised as a result of the remote monitoring will trigger a full site monitoring visit. To this end:

- Coordinated by LCTU Trial Manager and monitor, site initiation visits will be performed to enable training of local research personnel
- The Trial Manager at LCTU will document the completion of the initiation checklist for each site to verify appropriate approvals are in place prior to initiation of the site
- The Trial Manager will document, as part of the initiation checklist, that all relevant personnel have undergone trial specific training
- Data will be centrally monitored by the LCTU Monitor and Trial Manager to check:
  - Adverse Event reporting rates between centres
  - Screening, recruitment, and dropout rates between centres
  - Data entry consistency. The Data Manager and Trial Manager, with the Monitor, will follow-up on the data queries; Central monitoring reports will thus be generated for the TMG, who will oversee the activity and in accordance with the monitoring plan, will identify when additional intervention, e.g. site visits, should be undertaken.
  - Independent oversight of the trial will be provided by IDMC and independent members of the TSC.

Among the most important factors influencing the delivery of these quality objectives are:

- Minimising the burden on the clinicians working in overstretched LC clinics.
- Ensuring suitability of the participants and having access to the trial treatment without impacting on their other medical needs.
- Ensuring information given to the participants and the PIs in a timely and readily digestible fashion without adversely impacting on the patient care.
- To allow the treating physician to use their clinical judgement to decide whether any of the treatment arms are not suitable for the patient under their care.
- To collect comprehensive information on the mortality as well as morbidity of the LC status.
- In all aspects of the trial, any risks to the patients and well-being will by a key principle in that of proportionality. Risks associated with participation in the trial must be considered in the context of usual care.
