# Supplementary material for: STIMULATE-ICP: A pragmatic, multi-centre, cluster randomised trial of an integrated care pathway with a nested, Phase III, open label, adaptive platform randomised drug trial in individuals with Long COVID: A structured protocol
Source: PLoS One. 2023 Feb 15;18(2):e0272472. doi: 10.1371/journal.pone.0272472 (PMC9931100; doi:10.1371/journal.pone.0272472)
Supplement: Attachment — Submitted filename: Response to reviewers_PLOS One.docx [file pone.0272472.s011.docx]

**RESPONSE TO REVIEWERS**

PONE-D-22-20207

STIMULATE-ICP: A pragmatic, multi-centre, cluster randomised trial of an integrated care pathway with a nested, Phase III, open label, adaptive platform randomised drug trial in individuals with Long COVID: a structured protocol

PLOS ONE

Dear Dr. Banerjee,

Thank you for submitting your manuscript to PLOS ONE. After careful consideration, we feel that it has merit but does not fully meet PLOS ONE’s publication criteria as it currently stands. Therefore, we invite you to submit a revised version of the manuscript that addresses the points raised during the review process.

The manuscript has been evaluated by one reviewer, and their comments are available below.

The reviewer has raised concerns regarding the reporting and conceptualization of the study.

Could you please revise the manuscript to carefully address the concerns raised?

Please submit your revised manuscript by Oct 24 2022 11:59PM. If you will need more time than this to complete your revisions, please reply to this message or contact the journal office at [plosone@plos.org](mailto:plosone@plos.org). When you're ready to submit your revision, log on to <https://www.editorialmanager.com/pone/> and select the 'Submissions Needing Revision' folder to locate your manuscript file.

Please include the following items when submitting your revised manuscript:

- A rebuttal letter that responds to each point raised by the academic editor and reviewer(s). You should upload this letter as a separate file labeled 'Response to Reviewers'.
- A marked-up copy of your manuscript that highlights changes made to the original version. You should upload this as a separate file labeled 'Revised Manuscript with Track Changes'.
- An unmarked version of your revised paper without tracked changes. You should upload this as a separate file labeled 'Manuscript'.

**Thanks-we have provided these documents as requested.**

If you would like to make changes to your financial disclosure, please include your updated statement in your cover letter. Guidelines for resubmitting your figure files are available below the reviewer comments at the end of this letter.

If applicable, we recommend that you deposit your laboratory protocols in protocols.io to enhance the reproducibility of your results. Protocols.io assigns your protocol its own identifier (DOI) so that it can be cited independently in the future. For instructions see: [https://journals.plos.org/plosone/s/submission-guidelines#loc-laboratory-protocols](https://eur01.safelinks.protection.outlook.com/?url=https%3A%2F%2Fjournals.plos.org%2Fplosone%2Fs%2Fsubmission-guidelines%23loc-laboratory-protocols&data=05%7C01%7Cami.banerjee%40ucl.ac.uk%7C8766dccf3cdf41309ba308da946e5e11%7C1faf88fea9984c5b93c9210a11d9a5c2%7C0%7C0%7C637985499579938530%7CUnknown%7CTWFpbGZsb3d8eyJWIjoiMC4wLjAwMDAiLCJQIjoiV2luMzIiLCJBTiI6Ik1haWwiLCJXVCI6Mn0%3D%7C3000%7C%7C%7C&sdata=1FsCGqxoZaYY6xuN5WFo4GB%2BLPVW3CBl0kQ4HQZTlxY%3D&reserved=0). Additionally, PLOS ONE offers an option for publishing peer-reviewed Lab Protocol articles, which describe protocols hosted on protocols.io. Read more information on sharing protocols at [https://plos.org/protocols?utm_medium=editorial-email&utm_source=authorletters&utm_campaign=protocols](https://eur01.safelinks.protection.outlook.com/?url=https%3A%2F%2Fplos.org%2Fprotocols%3Futm_medium%3Deditorial-email%26utm_source%3Dauthorletters%26utm_campaign%3Dprotocols&data=05%7C01%7Cami.banerjee%40ucl.ac.uk%7C8766dccf3cdf41309ba308da946e5e11%7C1faf88fea9984c5b93c9210a11d9a5c2%7C0%7C0%7C637985499580094093%7CUnknown%7CTWFpbGZsb3d8eyJWIjoiMC4wLjAwMDAiLCJQIjoiV2luMzIiLCJBTiI6Ik1haWwiLCJXVCI6Mn0%3D%7C3000%7C%7C%7C&sdata=59Jl%2Bx1vXlCG0crPNYTCH5X%2FFbr6Ywvx%2FTS9jKLoHx4%3D&reserved=0).

We look forward to receiving your revised manuscript.

Kind regards,

Johannes Stortz
Staff Editor
PLOS ONE

Journal Requirements:

When submitting your revision, we need you to address these additional requirements.

1. Please ensure that your manuscript meets PLOS ONE's style requirements, including those for file naming. The PLOS ONE style templates can be found at
   <https://journals.plos.org/plosone/s/file?id=wjVg/PLOSOne_formatting_sample_main_body.pdf> and 
   <https://journals.plos.org/plosone/s/file?id=ba62/PLOSOne_formatting_sample_title_authors_affiliations.pdf>

**Thank you. We have now ensured that we meet the naming requirements for PLOS One.**

2. Please amend your current ethics statement to address the following concerns:
   a) Did participants provide their written or verbal informed consent to participate in this study?

**All participants provide written consent and this has been amended in the revised manuscript.**

b) If consent was verbal, please explain i) why written consent was not obtained, ii) how you documented participant consent, and iii) whether the ethics committees/IRB approved this consent procedure.

**Not applicable. All consent procedures were approved by the ethics committee and IRB.**

3. We note that the grant information you provided in the ‘Funding Information’ and ‘Financial Disclosure’ sections do not match.

When you resubmit, please ensure that you provide the correct grant numbers for the awards you received for your study in the ‘Funding Information’ section.

**Thank you- the financial disclosure section is the “competing interests” which includes all the additional sources of funding of individual coauthors which are not all relevant to this trial/study. Therefore the sections do not match. The grant award number if the NIHR number. No other grant award numbers are relevant.**

4. Thank you for stating the following in the Funding Section of your manuscript:

"This trial is part of a National Institute for Health Research (NIHR: COV-LT2-0043)-funded programme (Figure 2)(17), with epidemiologic and mixed methods studies(18), including care inequalities and transferability to other LTCs (IRAS 303958) and this complex intervention trial (IRAS 1004698). Funding for the transport, storage, processing and biobanking of blood and MRI is supported by Perspectum Ltd. The funders had and will not have a role in study design, data collection and analysis, decision to publish, or preparation of the manuscript. The views and opinions expressed therein are those of the authors and do not necessarily reflect those of the NIHR, NHS, the Department of Health and Social Care, or the sponsor."

We note that you have provided funding information that is not currently declared in your Funding Statement. However, funding information should not appear in the Acknowledgments section or other areas of your manuscript. We will only publish funding information present in the Funding Statement section of the online submission form. 
Please remove any funding-related text from the manuscript and let us know how you would like to update your Funding Statement.

**We confirm that we have removed funding information from the manuscript. The funding statement above is the correct statement:**

**"This trial is part of a National Institute for Health Research (NIHR: COV-LT2-0043)-funded programme (Figure 2)(17), with epidemiologic and mixed methods studies(18), including care inequalities and transferability to other LTCs (IRAS 303958) and this complex intervention trial (IRAS 1004698). Funding for the transport, storage, processing and biobanking of blood and MRI is supported by Perspectum Ltd. The funders had and will not have a role in study design, data collection and analysis, decision to publish, or preparation of the manuscript. The views and opinions expressed therein are those of the authors and do not necessarily reflect those of the NIHR, NHS, the Department of Health and Social Care, or the sponsor."**

Currently, your Funding Statement reads as follows:

"This trial is part of a National Institute for Health Research-funded programme  (NIHR: COV-LT2-0043). The CI is AB.

Funding for the transport, storage, processing and biobanking of blood and MRI is supported by Perspectum Ltd (CI-AB).

The funders had and will not have a role in study design, data collection and analysis, decision to publish, or preparation of the manuscript."

Please include your amended statements within your cover letter; we will change the online submission form on your behalf.

**The amended statement is in the cover letter as requested.**

5. Thank you for stating the following in the Competing Interests section:

**"****We have read the journal's policy and the authors of this manuscript have the following competing interests:**

**WDS holds research grants from Bayer, Novo Nordisk and Novartis and has received speaker honoraria from AstraZeneca, Bayer, Bristol-Myers Squibb, Merck, Napp, Novartis, Novo Nordisk and Takeda, outside the submitted work. WDS is supported by the NIHR Exeter Clinical Research Facility and the NIHR Collaboration for Leadership in Applied Health Research and Care (CLAHRC) for the South West Peninsula. MGC has received honoraria, fees for advisory boards, and non-financial support from AstraZeneca, BI, Chiesi, GSK, Novartis, and Pfizer and grants from AstraZeneca, BI, Chiesi, and Pfizer. CR is director of Living With, which developed Living With COVID RecoveryTM and other digital health interventions used in healthcare systems including the NHS. RB is CEO of Perspectum, which developed CoverscanTM. AB has received research funding from NIHR, British Medical Association, Astra Zeneca, National Institute of Aging and European Union. AB is a Trustee of Long COVID SoS. All other authors report no competing interests."**

Please confirm that this does not alter your adherence to all PLOS ONE policies on sharing data and materials, by including the following statement: "This does not alter our adherence to  PLOS ONE policies on sharing data and materials.” (as detailed online in our guide for authors <http://journals.plos.org/plosone/s/competing-interests>).  If there are restrictions on sharing of data and/or materials, please state these. Please note that we cannot proceed with consideration of your article until this information has been declared.

**We confirm this is the case and have included this statement in the revised manuscript.**

Please include your updated Competing Interests statement in your cover letter; we will change the online submission form on your behalf.

6. One of the noted authors is a group or consortium STIMULATE-ICP trial team. In addition to naming the author group, please list the individual authors and affiliations within this group in the acknowledgments section of your manuscript. Please also indicate clearly a lead author for this group along with a contact email address.

[Note: HTML markup is below. Please do not edit.]

**Thanks. This group has now been amended. The lead person for this group is the corresponding author (Amitava Banerjee, CI of the STIMULATE-ICP study). We have listed all the STIMULATE-ICP team members under each of their affiliations.**

Reviewers' comments:

Reviewer's Responses to Questions

**Comments to the Author**

1. Does the manuscript provide a valid rationale for the proposed study, with clearly identified and justified research questions?

The research question outlined is expected to address a valid academic problem or topic and contribute to the base of knowledge in the field.

Reviewer #1: Yes

2. Is the protocol technically sound and planned in a manner that will lead to a meaningful outcome and allow testing the stated hypotheses?

The manuscript should describe the methods in sufficient detail to prevent undisclosed flexibility in the experimental procedure or analysis pipeline, including sufficient outcome-neutral conditions (e.g. necessary controls, absence of floor or ceiling effects) to test the proposed hypotheses and a statistical power analysis where applicable. As there may be aspects of the methodology and analysis which can only be refined once the work is undertaken, authors should outline potential assumptions and explicitly describe what aspects of the proposed analyses, if any, are exploratory.

Reviewer #1: Yes

3. Is the methodology feasible and described in sufficient detail to allow the work to be replicable?

Descriptions of methods and materials in the protocol should be reported in sufficient detail for another researcher to reproduce all experiments and analyses. The protocol should describe the appropriate controls, sample size calculations, and replication needed to ensure that the data are robust and reproducible.

Reviewer #1: Yes

4. Have the authors described where all data underlying the findings will be made available when the study is complete?

The [PLOS Data policy](https://eur01.safelinks.protection.outlook.com/?url=https%3A%2F%2Fjournals.plos.org%2Fplosone%2Fs%2Fmaterials-and-software-sharing&data=05%7C01%7Cami.banerjee%40ucl.ac.uk%7C8766dccf3cdf41309ba308da946e5e11%7C1faf88fea9984c5b93c9210a11d9a5c2%7C0%7C0%7C637985499580094093%7CUnknown%7CTWFpbGZsb3d8eyJWIjoiMC4wLjAwMDAiLCJQIjoiV2luMzIiLCJBTiI6Ik1haWwiLCJXVCI6Mn0%3D%7C3000%7C%7C%7C&sdata=tarUBGeyGqtBRVSD%2Fepz6ECONdxPY2FzLQ9xe3acdb4%3D&reserved=0) requires authors to make all data underlying the findings described in their manuscript fully available without restriction, with rare exception, at the time of publication. The data should be provided as part of the manuscript or its supporting information, or deposited to a public repository. For example, in addition to summary statistics, the data points behind means, medians and variance measures should be available. If there are restrictions on publicly sharing data—e.g. participant privacy or use of data from a third party—those must be specified.

Reviewer #1: Yes

5. Is the manuscript presented in an intelligible fashion and written in standard English?

PLOS ONE does not copyedit accepted manuscripts, so the language in submitted articles must be clear, correct, and unambiguous. Any typographical or grammatical errors should be corrected at revision, so please note any specific errors here.

Reviewer #1: Yes

6. Review Comments to the Author

Please use the space provided to explain your answers to the questions above and, if applicable, provide comments about issues authors must address before this protocol can be accepted for publication. You may also include additional comments for the author, including concerns about research or publication ethics.

You may also provide optional suggestions and comments to authors that they might find helpful in planning their study.

(Please upload your review as an attachment if it exceeds 20,000 characters)

Reviewer #1: This felt long (at about 6,500 words) and almost a full protocol. My main suggestions are to:
Abbreviate for the journal manuscript to focus on the main design features. The regulatory and logistical aspects could be left out and the current manuscript provided more as an appendix.
In line with this, there were many abbreviations that may be familiar to those in the NIHR context but are not to international readers. I suggest the authors look through the manuscript and spell out most of these abbreviations for readability. An example is what does WP mean in Figure 2?

**We thank the reviewer for this helpful remark. We have abbreviated the manuscript as requested. We have removed most of the abbreviations from the text. We have also removed a lot of the logistic and regulatory detail for brevity and readability, as suggested, and put in Appendix 2 and Appendix 8. Also, we have provided a key for abbreviations in the figure. We have reduced the text to less than 5500 words.**

I was also left with little insight into the actual interventions and the background to those. I would like more details about each intervention - description of what they are exactly, brief background literature review, rationale for choosing these. For example, what is the Coverscan and how is it thought that a multi-organ MRI will help patients?

**Thanks for these comments. We have significantly changed the text into the introduction to make the rationale for the study clearer. The literature review suggested is already part of the introduction. A more systematic literature review is beyond scope of this study protocol. There was already rationale in the original manuscript for the trial of the integrated care pathway approach, the drug platform trial and the choice of the drugs (in Appendix 1), which we have retained in the revised version.**

**There is now a sentence with a clearer description of Coverscan and its potential role:**

**“Moreover, where up to 70% of individuals have mild organ impairment using CoverscanTM, a multi-organ MRI protocol(15), an early supported investigation strategy with multi-organ MRI may direct management, as well as better defining the syndrome, with relevance to other long-term conditions.”**

**We have also added justification of the Living with Covid Recovery digital rehabilitation intervention:**

**“Although digital rehabilitation platforms (e.g. Living with COVID RecoveryTM) may be scalable, particularly with strained rehabilitation services, and already being used in some centres, they are not yet evaluated in trials(16).”**

In the analysis section, it would be helpful if a figure could be provided that illustrates the various different populations for analyses. Either as a stand-alone figure, or indicated on Figure 3.

**Thanks for this suggestion. We have now annotated Figure 2 (which was the figure 3 in the previous manuscript) to show the populations for analysis and we have referred to these in the text.**

7. PLOS authors have the option to publish the peer review history of their article ([what does this mean?](https://eur01.safelinks.protection.outlook.com/?url=https%3A%2F%2Fjournals.plos.org%2Fplosone%2Fs%2Feditorial-and-peer-review-process%23loc-peer-review-history&data=05%7C01%7Cami.banerjee%40ucl.ac.uk%7C8766dccf3cdf41309ba308da946e5e11%7C1faf88fea9984c5b93c9210a11d9a5c2%7C0%7C0%7C637985499580094093%7CUnknown%7CTWFpbGZsb3d8eyJWIjoiMC4wLjAwMDAiLCJQIjoiV2luMzIiLCJBTiI6Ik1haWwiLCJXVCI6Mn0%3D%7C3000%7C%7C%7C&sdata=WM0l%2FeOj2alWf%2Fy8L43iKx%2FV2rYy2e1APbZJkKzI12U%3D&reserved=0)). If published, this will include your full peer review and any attached files.


If you choose “no”, your identity will remain anonymous but your review may still be made public.


**Do you want your identity to be public for this peer review?** For information about this choice, including consent withdrawal, please see our [Privacy Policy](https://eur01.safelinks.protection.outlook.com/?url=https%3A%2F%2Fwww.plos.org%2Fprivacy-policy&data=05%7C01%7Cami.banerjee%40ucl.ac.uk%7C8766dccf3cdf41309ba308da946e5e11%7C1faf88fea9984c5b93c9210a11d9a5c2%7C0%7C0%7C637985499580094093%7CUnknown%7CTWFpbGZsb3d8eyJWIjoiMC4wLjAwMDAiLCJQIjoiV2luMzIiLCJBTiI6Ik1haWwiLCJXVCI6Mn0%3D%7C3000%7C%7C%7C&sdata=ZVYwH5iY7sSsx5nkMMT1c%2BdcB9f8SKRgOwWB%2F%2B%2BdpxM%3D&reserved=0).

Reviewer #1: No

[NOTE: If reviewer comments were submitted as an attachment file, they will be attached to this email and accessible via the submission site. Please log into your account, locate the manuscript record, and check for the action link "View Attachments". If this link does not appear, there are no attachment files.]

While revising your submission, please upload your figure files to the Preflight Analysis and Conversion Engine (PACE) digital diagnostic tool, [https://pacev2.apexcovantage.com/](https://eur01.safelinks.protection.outlook.com/?url=https%3A%2F%2Fpacev2.apexcovantage.com%2F&data=05%7C01%7Cami.banerjee%40ucl.ac.uk%7C8766dccf3cdf41309ba308da946e5e11%7C1faf88fea9984c5b93c9210a11d9a5c2%7C0%7C0%7C637985499580094093%7CUnknown%7CTWFpbGZsb3d8eyJWIjoiMC4wLjAwMDAiLCJQIjoiV2luMzIiLCJBTiI6Ik1haWwiLCJXVCI6Mn0%3D%7C3000%7C%7C%7C&sdata=gMbQwd9qTmzPEaex%2FHTdOi4qW7ThtPqhKk73yap8RN4%3D&reserved=0). PACE helps ensure that figures meet PLOS requirements. To use PACE, you must first register as a user. Registration is free. Then, login and navigate to the UPLOAD tab, where you will find detailed instructions on how to use the tool. If you encounter any issues or have any questions when using PACE, please email PLOS at [figures@plos.org](mailto:figures@plos.org). Please note that Supporting Information files do not need this step.
