# Supplementary material for: STIMULATE-ICP: A pragmatic, multi-centre, cluster randomised trial of an integrated care pathway with a nested, Phase III, open label, adaptive platform randomised drug trial in individuals with Long COVID: A structured protocol
Source: PLoS One. 2023 Feb 15;18(2):e0272472. doi: 10.1371/journal.pone.0272472 (PMC9931100; doi:10.1371/journal.pone.0272472)
Supplement: Attachment — Submitted filename: Response to reviewers December 2022.docx [file pone.0272472.s012.docx]

**RESPONSE TO REVIEWERS**

PONE-D-22-20207R1

STIMULATE-ICP: A pragmatic, multi-centre, cluster randomised trial of an integrated care pathway with a nested, Phase III, open label, adaptive platform randomised drug trial in individuals with Long COVID: a structured protocol

PLOS ONE

Dear Dr. Amitava,

Thank you for submitting your manuscript to PLOS ONE. After careful consideration, we feel that it has merit but does not fully meet PLOS ONE’s publication criteria as it currently stands. Therefore, we invite you to submit a revised version of the manuscript that addresses the points raised during the review process.

Please submit your revised manuscript by Jan 15 2023 11:59PM. If you will need more time than this to complete your revisions, please reply to this message or contact the journal office at [plosone@plos.org](mailto:plosone@plos.org). When you're ready to submit your revision, log on to <https://www.editorialmanager.com/pone/> and select the 'Submissions Needing Revision' folder to locate your manuscript file.

Please include the following items when submitting your revised manuscript:

- A rebuttal letter that responds to each point raised by the academic editor and reviewer(s). You should upload this letter as a separate file labeled 'Response to Reviewers'.
- A marked-up copy of your manuscript that highlights changes made to the original version. You should upload this as a separate file labeled 'Revised Manuscript with Track Changes'.
- An unmarked version of your revised paper without tracked changes. You should upload this as a separate file labeled 'Manuscript'.

**Thanks- we have included these documents in the revised submission.**

If you would like to make changes to your financial disclosure, please include your updated statement in your cover letter. Guidelines for resubmitting your figure files are available below the reviewer comments at the end of this letter.

If applicable, we recommend that you deposit your laboratory protocols in protocols.io to enhance the reproducibility of your results. Protocols.io assigns your protocol its own identifier (DOI) so that it can be cited independently in the future. For instructions see: [https://journals.plos.org/plosone/s/submission-guidelines#loc-laboratory-protocols](https://eur01.safelinks.protection.outlook.com/?url=https%3A%2F%2Fjournals.plos.org%2Fplosone%2Fs%2Fsubmission-guidelines%23loc-laboratory-protocols&data=05%7C01%7Cami.banerjee%40ucl.ac.uk%7C2494728db1594d2bd02908dad3aa9230%7C1faf88fea9984c5b93c9210a11d9a5c2%7C0%7C0%7C638055026799853754%7CUnknown%7CTWFpbGZsb3d8eyJWIjoiMC4wLjAwMDAiLCJQIjoiV2luMzIiLCJBTiI6Ik1haWwiLCJXVCI6Mn0%3D%7C3000%7C%7C%7C&sdata=vq7VEzkuaCwynCfiRMXjFdA1M3hpwop90bCmnz4hciQ%3D&reserved=0). Additionally, PLOS ONE offers an option for publishing peer-reviewed Lab Protocol articles, which describe protocols hosted on protocols.io. Read more information on sharing protocols at [https://plos.org/protocols?utm_medium=editorial-email&utm_source=authorletters&utm_campaign=protocols](https://eur01.safelinks.protection.outlook.com/?url=https%3A%2F%2Fplos.org%2Fprotocols%3Futm_medium%3Deditorial-email%26utm_source%3Dauthorletters%26utm_campaign%3Dprotocols&data=05%7C01%7Cami.banerjee%40ucl.ac.uk%7C2494728db1594d2bd02908dad3aa9230%7C1faf88fea9984c5b93c9210a11d9a5c2%7C0%7C0%7C638055026799853754%7CUnknown%7CTWFpbGZsb3d8eyJWIjoiMC4wLjAwMDAiLCJQIjoiV2luMzIiLCJBTiI6Ik1haWwiLCJXVCI6Mn0%3D%7C3000%7C%7C%7C&sdata=N7FSIg93BOyIRWsmXvrR3yuQWFJliDF8XJfO98v7MC4%3D&reserved=0).
**Our protocol is already in the preprint repository (Medrxiv):** [**https://www.medrxiv.org/content/10.1101/2022.07.21.22277893v1**](https://www.medrxiv.org/content/10.1101/2022.07.21.22277893v1) **. Our trial is also registered at ISRCTN https://www.isrctn.com/ISRCTN10665760**

We look forward to receiving your revised manuscript.

Kind regards,

Hideo Kato
Academic Editor
PLOS ONE

Journal Requirements:

Additional Editor Comments:

The authors should take seriously in account the comments of the reviewers.


[Note: HTML markup is below. Please do not edit.]

Reviewers' comments:

Reviewer's Responses to Questions

**Comments to the Author**

1. Does the manuscript provide a valid rationale for the proposed study, with clearly identified and justified research questions?

The research question outlined is expected to address a valid academic problem or topic and contribute to the base of knowledge in the field.

Reviewer #1: Yes

Reviewer #2: Yes

**Thanks**

2. Is the protocol technically sound and planned in a manner that will lead to a meaningful outcome and allow testing the stated hypotheses?

The manuscript should describe the methods in sufficient detail to prevent undisclosed flexibility in the experimental procedure or analysis pipeline, including sufficient outcome-neutral conditions (e.g. necessary controls, absence of floor or ceiling effects) to test the proposed hypotheses and a statistical power analysis where applicable. As there may be aspects of the methodology and analysis which can only be refined once the work is undertaken, authors should outline potential assumptions and explicitly describe what aspects of the proposed analyses, if any, are exploratory.

Reviewer #1: Yes

Reviewer #2: Yes

**Thanks**

3. Is the methodology feasible and described in sufficient detail to allow the work to be replicable?


Descriptions of methods and materials in the protocol should be reported in sufficient detail for another researcher to reproduce all experiments and analyses. The protocol should describe the appropriate controls, sample size calculations, and replication needed to ensure that the data are robust and reproducible.

Reviewer #1: Yes

Reviewer #2: Yes

**Thanks**

4. Have the authors described where all data underlying the findings will be made available when the study is complete?

The [PLOS Data policy](https://eur01.safelinks.protection.outlook.com/?url=https%3A%2F%2Fjournals.plos.org%2Fplosone%2Fs%2Fmaterials-and-software-sharing&data=05%7C01%7Cami.banerjee%40ucl.ac.uk%7C2494728db1594d2bd02908dad3aa9230%7C1faf88fea9984c5b93c9210a11d9a5c2%7C0%7C0%7C638055026799853754%7CUnknown%7CTWFpbGZsb3d8eyJWIjoiMC4wLjAwMDAiLCJQIjoiV2luMzIiLCJBTiI6Ik1haWwiLCJXVCI6Mn0%3D%7C3000%7C%7C%7C&sdata=IbKHQTYhkwrGev4tNPNnhxASvAgXpAn8%2Fjbvu2tu45I%3D&reserved=0) requires authors to make all data underlying the findings described in their manuscript fully available without restriction, with rare exception, at the time of publication. The data should be provided as part of the manuscript or its supporting information, or deposited to a public repository. For example, in addition to summary statistics, the data points behind means, medians and variance measures should be available. If there are restrictions on publicly sharing data—e.g. participant privacy or use of data from a third party—those must be specified.

Reviewer #1: Yes

Reviewer #2: No

**Thanks- it is unclear why Reviewer 2 has written “No” as we do not provide results/findings in this protocol (since the trial is currently recruiting). We have provided data supporting the choice of drugs as supporting (supplementary) information. See response below.**

5. Is the manuscript presented in an intelligible fashion and written in standard English?

PLOS ONE does not copyedit accepted manuscripts, so the language in submitted articles must be clear, correct, and unambiguous. Any typographical or grammatical errors should be corrected at revision, so please note any specific errors here.

Reviewer #1: Yes

Reviewer #2: Yes

**Thanks**

6. Review Comments to the Author

Please use the space provided to explain your answers to the questions above and, if applicable, provide comments about issues authors must address before this protocol can be accepted for publication. You may also include additional comments for the author, including concerns about research or publication ethics.

You may also provide optional suggestions and comments to authors that they might find helpful in planning their study.


(Please upload your review as an attachment if it exceeds 20,000 characters)

Reviewer #1: Thank you, the manuscript is much improved.

I still think there needs to be additional rationale provided for use of Coverscan. The authors have still not addressed the previous question sufficiently. It is not clear to me how a multi-organ MRI will result in improved outcomes. Will there be an algorithmic management approach based on the findings? For example, what is proposed if there is pancreatic or renal involvement in the absence of other markers of organ dysfunction? I appreciate the work showing that 70% of a previous cohort had evidence of at least single organ involvement, what is not clear is whether this has any clinical implications and how it might result in changes in management that would ultimately benefit the patient.

**Thanks for this remark and apologies if the previous response was not clear. Part of the trial is to understand how the Coverscan may inform or influence care (or not), and it came on the backdrop of high numbers of patients seeking investigation for possible Long Covid with** **variable waiting times, variable resources for scanning and variable practices with frequent repeating of investigations and referrals to multiple specialists (see reference 3. Heightman M et al. 2021). A multi-organ MRI may improve outcomes in two ways, both by reducing time to treatment****. First, a negative finding by ruling out organ involvement and other disease early could reassure the clinician and the patient, rather than organising multiple investigations with multiple specialists via multiple referrals. Second, by flagging organ or multi-organ dysfunction, the MRI may trigger appropriate specialist referral earlier and lead to earlier appropriate management. Of course, the trial may show no effect of Coverscan.**

**The reviewer is right that we do not know yet the clinical implications of the single or multiple organ involvement post-COVID and the rate of resolution- these are being investigated in the trial.** **Therefore, we did not have or develop an algorithmic management approach (i.e. clinical decision support) or “manual” because the organ dysfunction in Long Covid is still being defined and a manual would involve approaches to any possible finding in lungs, heart, liver, pancreas and kidneys, which is not feasible. In answer to the reviewer’s question, “…what is proposed if there is pancreatic or renal involvement in the absence of other markers of organ dysfunction?”, there would be specialist referral to assess the potential relevance or importance of such a finding, which may be incidental and not clinically relevant.**

**The Coverscan has a standardised report. For the first 100 scans across the 6 sites, there is report by an independent radiologist, and a virtual, weekly multidisciplinary meeting to discuss any cases and potential implications of findings, management and/or referral to specialists. To summarise, part of the trial is to find out whether the Coverscan changes and/or improves management of people with Long Covid.**

**We have added text to the revised manuscript to cover some of these complex issues.**

Reviewer #2: No mentioning of data safety monitoring board.

**We do mention the Independent Data Monitoring Committee which is the data safety monitoring board (we have now explained this in the new revised text).**

Futility analysis was not considered in the interim analysis.

**Thank you for this important remark. We actually had considered futility analysis in the interim analysis and our study but both our team and the Independent Data Monitoring Committee felt that futility analysis should not be included because:**

1. **Our focus is on drugs and integrated pathway components which work**
2. **Due to the complex design, a futility analysis plan across different drug arms and different part of the cluster randomised trial was likely to be complicated and very difficult to implement.**
3. **In a trial with several moving parts and a moving target in terms of the disease itself, futility analyses may lead to underpowering of arms of the study.**

**We and our Independent Data Monitoring Committee (who we consulted again) feel that the plans for interim analysis are robust.**

**We have added a sentence regarding futility analysis to reflect these issues in the revised manuscript.**

P value <0.001 is not clearly justified in the interim analysis.

**We do have a sentence in the manuscript already to justify the p value<0.001:**

**“A statistically significant p-value at the 0.001 two-sided level is required for a pathway to be deemed superior at interim analysis. This is to account for multiple testing (Bonferroni type correction). The analysis at 4520 individuals in total will be at the conventional 0.05 two-sided level. There are practical and operational reasons why a single pathway cannot graduate and become the standard pathway within the recruitment period of this trial.”**

7. PLOS authors have the option to publish the peer review history of their article ([what does this mean?](https://eur01.safelinks.protection.outlook.com/?url=https%3A%2F%2Fjournals.plos.org%2Fplosone%2Fs%2Feditorial-and-peer-review-process%23loc-peer-review-history&data=05%7C01%7Cami.banerjee%40ucl.ac.uk%7C2494728db1594d2bd02908dad3aa9230%7C1faf88fea9984c5b93c9210a11d9a5c2%7C0%7C0%7C638055026799853754%7CUnknown%7CTWFpbGZsb3d8eyJWIjoiMC4wLjAwMDAiLCJQIjoiV2luMzIiLCJBTiI6Ik1haWwiLCJXVCI6Mn0%3D%7C3000%7C%7C%7C&sdata=jivExzJxD%2Bi2G1aRFjPg3QdVMh%2FBfWICT1Qht%2B%2BcKJg%3D&reserved=0)). If published, this will include your full peer review and any attached files.


If you choose “no”, your identity will remain anonymous but your review may still be made public.


**Do you want your identity to be public for this peer review?** For information about this choice, including consent withdrawal, please see our [Privacy Policy](https://eur01.safelinks.protection.outlook.com/?url=https%3A%2F%2Fwww.plos.org%2Fprivacy-policy&data=05%7C01%7Cami.banerjee%40ucl.ac.uk%7C2494728db1594d2bd02908dad3aa9230%7C1faf88fea9984c5b93c9210a11d9a5c2%7C0%7C0%7C638055026799853754%7CUnknown%7CTWFpbGZsb3d8eyJWIjoiMC4wLjAwMDAiLCJQIjoiV2luMzIiLCJBTiI6Ik1haWwiLCJXVCI6Mn0%3D%7C3000%7C%7C%7C&sdata=HJLq44z366PR7hi1tdOUXKrcXwtcK69Cd0FXU5BtUek%3D&reserved=0).

Reviewer #1: No

Reviewer #2: No

[NOTE: If reviewer comments were submitted as an attachment file, they will be attached to this email and accessible via the submission site. Please log into your account, locate the manuscript record, and check for the action link "View Attachments". If this link does not appear, there are no attachment files.]

While revising your submission, please upload your figure files to the Preflight Analysis and Conversion Engine (PACE) digital diagnostic tool, [https://pacev2.apexcovantage.com/](https://eur01.safelinks.protection.outlook.com/?url=https%3A%2F%2Fpacev2.apexcovantage.com%2F&data=05%7C01%7Cami.banerjee%40ucl.ac.uk%7C2494728db1594d2bd02908dad3aa9230%7C1faf88fea9984c5b93c9210a11d9a5c2%7C0%7C0%7C638055026799853754%7CUnknown%7CTWFpbGZsb3d8eyJWIjoiMC4wLjAwMDAiLCJQIjoiV2luMzIiLCJBTiI6Ik1haWwiLCJXVCI6Mn0%3D%7C3000%7C%7C%7C&sdata=FfAwf11XCICO%2FJUWfo0u2o9qzvVlLQKVbJ%2FUudb1iBA%3D&reserved=0). PACE helps ensure that figures meet PLOS requirements. To use PACE, you must first register as a user. Registration is free. Then, login and navigate to the UPLOAD tab, where you will find detailed instructions on how to use the tool. If you encounter any issues or have any questions when using PACE, please email PLOS at [figures@plos.org](mailto:figures@plos.org). Please note that Supporting Information files do not need this step.

 Thanks.

*In compliance with data protection regulations, you may request that we remove your personal registration details at any time.* [*(Remove my information/details)*](https://eur01.safelinks.protection.outlook.com/?url=https%3A%2F%2Fwww.editorialmanager.com%2Fpone%2Flogin.asp%3Fa%3Dr&data=05%7C01%7Cami.banerjee%40ucl.ac.uk%7C2494728db1594d2bd02908dad3aa9230%7C1faf88fea9984c5b93c9210a11d9a5c2%7C0%7C0%7C638055026799853754%7CUnknown%7CTWFpbGZsb3d8eyJWIjoiMC4wLjAwMDAiLCJQIjoiV2luMzIiLCJBTiI6Ik1haWwiLCJXVCI6Mn0%3D%7C3000%7C%7C%7C&sdata=eRtx5X4nnEhH%2FzRom6m539Pj3aO5iEHzQyb%2FpmRfqJk%3D&reserved=0)*. Please contact the publication office if you have any questions.*
